# Acidogenesis‐Propelled Coordination Transition in Light‐Triggered Fe‐Polyphenol Polymer for Reactive Oxygen Species‐Augmented Antitumor Therapy

**DOI:** 10.1002/advs.202513361

**Published:** 2025-09-27

**Authors:** Ying Wan, Hui Liu, Lin Gao, Guanyu Tan, Kailin Li, Qiwei Tian, Shiping Yang, Lu An

**Affiliations:** ^1^ The Education Ministry Key Lab of Resource Chemistry Shanghai Municipal Education Committee Key Laboratory of Molecular Imaging Probes and Sensors Shanghai Key Laboratory of Rare Earth Functional Materials Shanghai Normal University Shanghai 200234 China; ^2^ Shanghai Key Laboratory of Molecular Imaging Jiading District Central Hospital Affiliated Shanghai University of Medicine and Health Sciences Shanghai University of Medicine and Health Sciences Shanghai 201318 China

**Keywords:** coordination transition, iron‐polyphenol, photoacid, reactive oxygen species, theranostics

## Abstract

Iron‐based Fenton agents have emerged as promising candidates for tumor therapy due to its excellent selectivity, yet their therapeutic potential is substantially constrained by inefficient Fe^3+^/Fe^2+^ conversion in the tumor microenvironment. Herein, based on a coordination engineering strategy, a light‐responsive Fe‐polyphenol coordination polymer (FeBPs), integrating Fe centers, Bodipy‐based photoacid generators, and PEG‐stabilized polyphenol ligands, is designed to explore how to accelerate the transformation efficiency of Fe^3+^ to Fe^2+^ by incorporating both internal and external factors. In terms of internal factors, upon irradiation at 630 nm, the FeBPs trigger the exposure of catalytic sites derived from the coordination transition nature of iron and polyphenol, induced by light‐triggered acidification. Additionally, the Fe^2+^ regeneration efficiency is also enhanced by changes in the external environment, such as a decrease in pH. Both the light triggered internal and external factors can amplify reactive oxygen species (ROS) fluxes, which disrupt mitochondrial function and induce cell apoptosis, achieving tumor‐specific homeostasis perturbation. In melanoma‐bearing mouse models, FeBPs exhibit complete tumor regression. The findings establish a paradigm for iron‐based therapeutics by harnessing acid‐triggered metal‐ligand cooperativity, overcoming critical limitations of pH dependency and inefficient Fe^3+^/Fe^2+^ conversion, and will provide a foundational framework for adaptive metallopolymeric theranostics.

## Graphical abstract

A novel iron‐polyphenol‐photoacid coordination polymer (FeBPs) is designed to achieve red light‐triggered external acidification and internal coordination regulation, where this light‐controlled acidification induces coordination changes that enhance Fe active site exposure and provide H^+^ for beneficial Fe^3+^/Fe^2+^ conversion, thereby accelerating ROS generation and significantly improving tumor therapy efficacy.

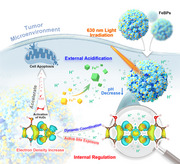

## 1 Introduction

Reactive oxygen species (ROS)‐based tumor therapy strategies, which utilize exogenous or endogenous substances to catalyze oxidative reactions, inducing intracellular oxidative stress that causes irreversible damage to biomolecules and triggers cell apoptosis, have emerged as promising approaches for cancer treatment.^[^ ^1^, ^2^ ^]^ These strategies mainly involve nanozymes with enzymatic‐like activities, photodynamic therapy using light‐activated photosensitizers and energy transfer to generate singlet oxygen (^1^O_2_), chemodynamic therapy based on Fenton or Fenton‐like reactions to produce highly reactive hydroxyl radical (•OH), and sonodynamic therapy employing ultrasound‐activated sonosensitizers.^[^ ^3^, ^4^, ^5^, ^6^, ^7^ ^]^ Notably, the Fe^3+^/Fe^2+^ redox cycle, which activates endogenous H_2_O_2_ in tumors to produce highly reactive •OH (*k* = 10^9^–10^10^ M^−1^ s^−1^), has attracted significant attention due to its tumor specificity.^[^ ^8^, ^9^, ^10^ ^]^ However, the inherent limitation of the Fenton reaction lies in the slow reduction rate of Fe^3+^ to Fe^2+^ (*k* = 0.001–0.01 M^−1^ s^−1^), reducing H_2_O_2_ cleavage and •OH generation rates, and thus hindering its application in tumor treatment.^[^ ^11^, ^12^ ^]^ Therefore, the key issue at present is how to accelerate this transformation by regulating both the internal and external environments.

The normally used strategy to regulate the rate of Fe^3+^ to Fe^2+^ is limited by the dual constraints of Fe^3+^ speciation dynamics and electron transfer limitations^[^ ^13^, ^14^, ^15^ ^]^ is external environments, such as pH or energy sources. Compared to physiological conditions, where Fe^3+^ predominantly exists as redox‐inherently sluggish polymeric species (such as [Fe_2_(OH)_2_]_4_^+^ and Fe(OH)_3_ colloids), a lower pH (below 3) favors the formation of active [Fe(OH)]^2+^ monomers, which enhances Fenton activity.^[^ ^16^, ^17^ ^]^ However, the acidic microenvironment within the tumor is difficult to facilitate the formation of this active monomer.^[^ ^18^ ^]^ High‐energy ultraviolet light has also been widely used to alter the oxidation state of Fe. For example, Youn et al. employed high‐energy ultraviolet light to enhance the ligand‐to‐metal charge transfer (LMCT) band of Fe^3+^ hydrates, thereby boosting Fe^2+^ recycling and •OH generation.^[^ ^19^ ^]^ However, its limited tissue penetration restricts its bioapplication. Directly altering the valence state of Fe is a common internal mechanism adjustment strategy, typically using reducing agents such as hydrogen sulfide (H_2_S) and glutathione (GSH).^[^ ^20^ ^]^ Our previous work demonstrated that H_2_S can reduce Fe^3+^ to Fe^2+^, establishing an active iron oxidation state that is beneficial for •OH generation.^[^ ^21^ ^]^ However, the strong bond strength of rigid covalent frameworks reduces the exposure of active sites, thereby limiting performance improvement.^[^ ^22^, ^23^ ^]^ Therefore, finding a way to ingeniously integrate internal and external factors to accelerate the transformation remains a significant challenge.

**Scheme 1:**
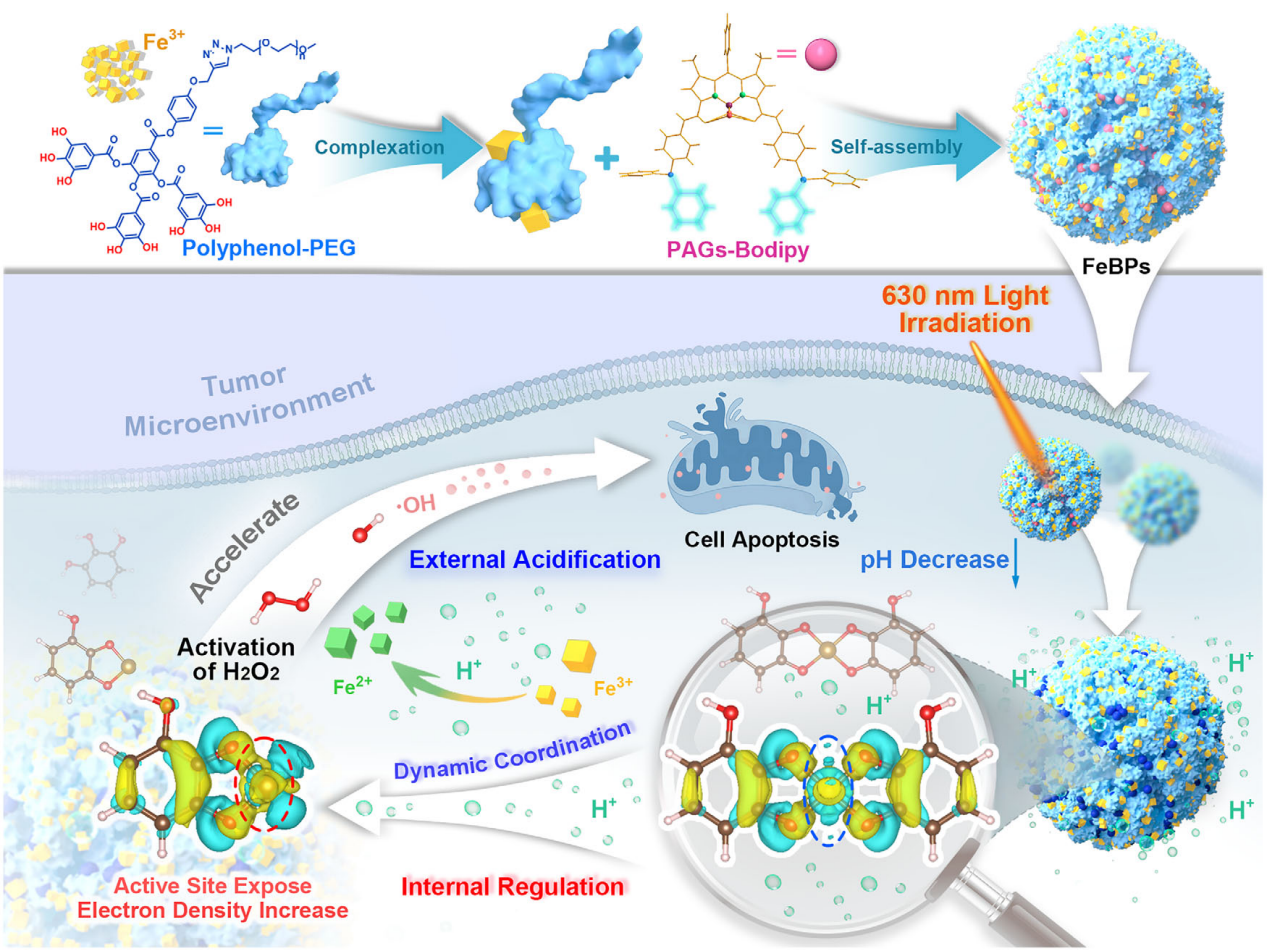
The mechanism of light‐triggered acidogenesis‐propelled coordination transition for amplified ROS in tumor therapy.

To achieve this, leveraging coordination chemistry, which has recently proven to enhance the cycling capacity of active sites,^[^ ^24^, ^25^, ^26^ ^]^ we designed a light‐responsive Fe‐polyphenol coordination polymer (FeBPs) to explore the Fe^3+^ to Fe^2+^ transformation by integrating both internal and external factors. Unlike rigid covalent frameworks, coordination compounds, with their tunable characteristics (e.g., bond strength, coordination number, and overall coordination environment), inherently respond to stimuli such as pH and redox potential, governing the kinetics of Fenton reactions.^[^ ^27^, ^28^ ^]^ Thus, a photoacid generators (PAGs‐Bodipy) with high H^+^ yield was introduced in this coordination polymer. Upon irradiation with 630 nm light, localized H^+^ release weakens the iron‐phenol coordination. This process exposes active Fe^2+^ sites, enhancing H_2_O_2_ activation for •OH generation. Simultaneously, the light‐triggered acidification prevents the formation of inactive Fe^3+^ hydrolysis intermediates, ensuring sustained Fe^2+^ availability and establishing a ROS amplification for effective anti‐tumor effect (**Scheme** **1**). This approach represents the first application of light‐controlled acidification‐induced coordination transition for sustainable Fe^2+^ generation and ROS amplification in anti‐tumor therapy, and offers an effective strategy for developing new multifunctional therapeutic formulations based on coordination polymers.

## 2 Synthesis and Characterization of FeBPs Coordination Polymer

**Figure 1:**
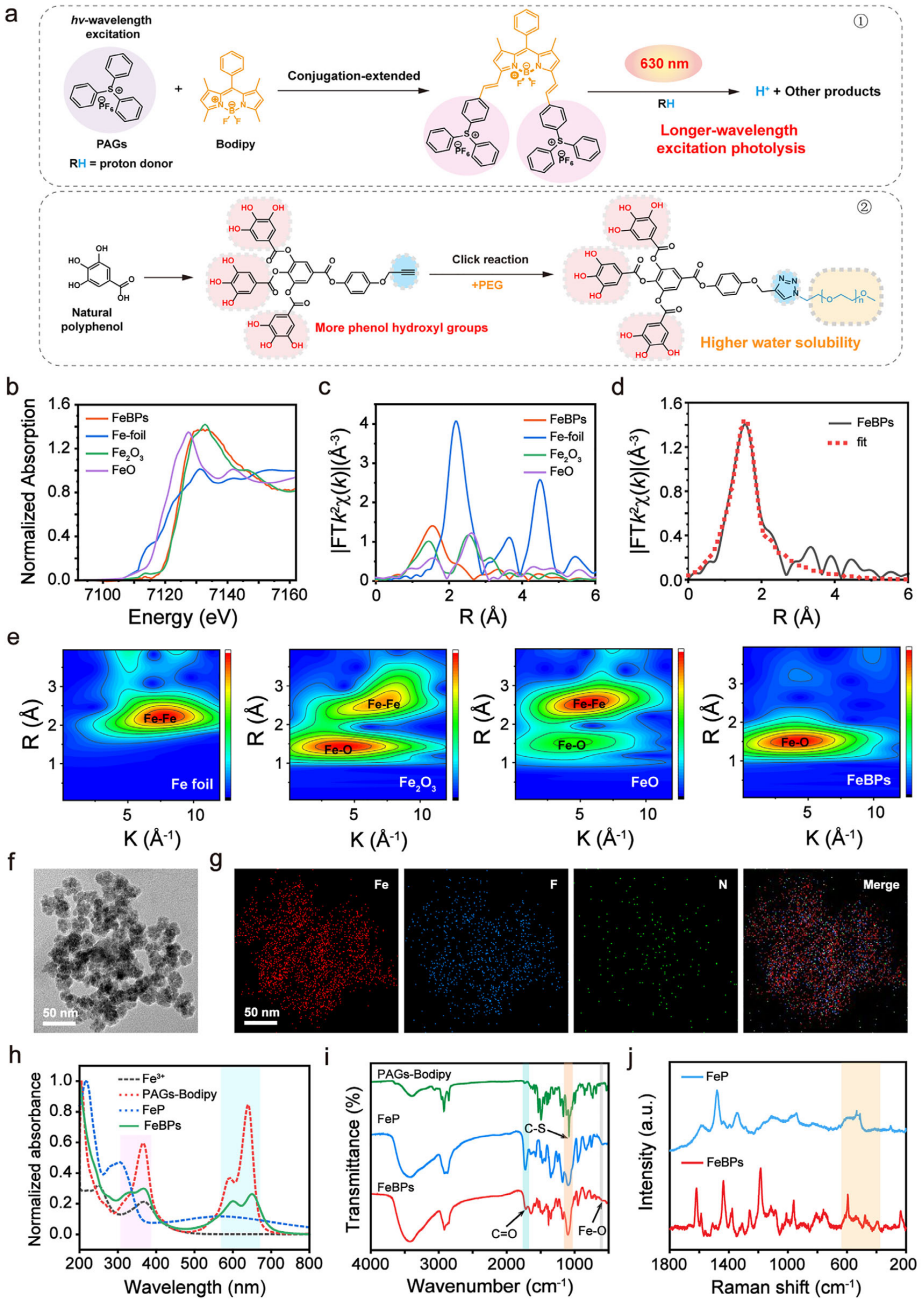
The characterization of FeBPs. a) Synthesis pathway of PAGs‐Bodipy and hydrophilic polyphenol‐PEG. b) Normalized Fe K‐edge X‐ray absorption near‐edge structure (XANES) spectra of Fe foil, FeO, Fe_2_O_3_, and FeBPs, and c) corresponding Fourier transform curves at R space. d) Nonlinear fitting of the EXAFS spectra for FeBPs (R‐space radial distributions). e) WT contour plots of k^2^‐weighted χ(k) signals from Fe foil, FeO, Fe_2_O_3_, and FeBPs, respectively. f) TEM image of FeBPs (scale bar: 50 nm). g) Elemental mapping of FeBPs confirming homogeneous distribution of Fe (red), F (blue), and N (green) (scale bar: 50 nm). h) UV–vis spectra of FeBPs, FeP, PAGs‐Bodipy, and Fe^3+^. i) FTIR spectra of FeBPs, FeP, and PAGs‐Bodipy. j) Raman spectra of FeBPs and FeP.

Photoacid generators are critical for controlled H^+^ release in physiological environments. To achieve high H^+^ yield and deep tissue‐penetrating activation, we designed a novel PAGs‐Bodipy system through Knoevenagel condensation of 3‐ and 5‐methyl‐substituted boron dipyrromethene (Bodipy) cores with aromatic aldehydes (**Figure** **1**a; Figures S1 and S2, Supporting Information). This strategy red‐shifted the absorption peak of PAGs‐Bodipy to 636 nm, closing to the near‐infrared (NIR) window for enhanced tissue penetration (Figure S3, Supporting Information). The resulting PAGs‐Bodipy exhibited a long photoluminescence lifetime, ensuring efficient light‐to‐acid conversion under 630 nm irradiation (Figure S4 and Table S1, Supporting Information). Hydrophilic polyphenol‐PEG was synthesized via click chemistry. Highly ordered dendritic polyphenol architectures enhanced complex‐water interactions via controlled phenolic hydroxyl amplification and addressed natural polyphenol coordination complexities.^[^ ^29^, ^30^ ^]^ The conjugation between alkynyl‐functionalized polyphenolic compounds and azide‐terminated PEG was confirmed by ^1^H NMR resonances (characteristic chemical shifts of the 1,2,3‐triazole ring appearing ≈8 ppm) and Fourier transform infrared (FTIR) vibrational modes (the telescopic vibration of the azide disappears at 2100 cm^−1^, Figures S5–S12, Supporting Information).

Subsequently, FeBPs coordination polymers were fabricated by coordinating Fe^3+^ with polyphenol‐PEG to create a primary Fe‐polyphenol‐PEG complex, followed by further assembly with PAGs‐Bodipy. To clarify the coordination environment and valence state of Fe in FeBPs, X‐ray absorption fine structure (XAFS) analysis was performed. As shown in Figure 1b, the absorption edge of Fe in FeBPs was located between those of standard Fe_2_O_3_ and FeO, directly confirming that Fe in FeBPs exists in a mixed valence state (Fe^2+^/Fe^3+^). Fourier transform of extended XAFS (FT‐EXAFS) spectra exhibited a dominant characteristic peak corresponding to Fe─O bonds, with an average coordination number of 4.4 ± 0.3 (Figure 1c; Table S2, Supporting Information)—indicating the coordination mode where one Fe binds to four phenolic hydroxyl groups.^[^ ^31^ ^]^ *R*‐Fit nonlinear fitting of the EXAFS spectra further verified the presence of Fe─O coordination (Figure 1d; Figures S13 and S14, Supporting Information), while wavelet transform (WT) contour plots of the k^2^‐weighted χ(k) signals clearly showed the vibrational features of Fe─O bonds (Figure 1e), providing additional evidence for the Fe─O‐dominated coordination environment. Transmission electron microscopy (TEM) and scanning electron microscopy revealed monodisperse spherical FeBPs with an average diameter of ≈25 nm (Figure 1f; Figure S15, Supporting Information). Energy‐dispersive X‐ray spectroscopy and elemental mapping confirmed homogeneous distribution of Fe, F, and N within the FeBPs (Figure 1g; Figure S16, Supporting Information), verifying the successful incorporation of PAGs‐Bodipy into the Fe‐polyphenol‐PEG coordination network. Inductively coupled plasma mass spectrometry analysis of Fe and B (B is a signature element of PAGs‐Bodipy) in FeBPs indicated a molar ratio of Fe to B of 6:1, further validating the effective integration of the core functional components (Table S3, Supporting Information).

To further verify the assembly and FeBPs’ structural‐functional integrity, the surface properties, spectral signatures of coordination, photophysical behavior, and stability were characterized. FeBPs exhibited a positive surface charge (+11.6 mV) compared to the negatively charged Fe‐polyphenol‐PEG (FeP, −20.1 mV), confirming successful PAGs‐Bodipy integration (Figure S17, Supporting Information). The UV–vis spectrum of FeBPs exhibited characteristic peaks at 600 and 650 nm, which were ascribed to PAGs‐Bodipy, and a broad absorption band from 300–350 nm was attributed to the aromatic *π–π* ^*^ transitions in polyphenol‐PEG (Figure 1h). Additionally, the fluorescence emission spectrum of FeBPs was consistent with that of free PAGs‐Bodipy, indicating that the photophysical properties of PAGs‐Bodipy were well preserved post‐assembly (Figure S18, Supporting Information). FTIR and Raman spectroscopy provided direct evidence of Fe^3+^‐polyphenol coordination, as well as the integration of each component in FeBPs. Peaks at 1720 cm^−1^ (C═O stretching of polyphenol‐PEG ester) and 1034 cm^−1^ (C─S vibration from PAGs‐Bodipy) confirmed component integration into FeBPs (Figure 1i).^[^ ^32^ ^]^ A distinct Fe─O stretching band at 600 cm^−1^ in FTIR spectrum and symmetric/asymmetric Fe─O vibrations in the Raman spectrum (400–600 cm^−1^) validated the Fe^3+^‐mediated coordination architecture (Figure 1j).^[^ ^33^ ^]^ In addition, FeBPs demonstrated excellent colloidal stability over 7 days, with minimal size variation and no aggregation (Figure S19, Supporting Information). This stability was further confirmed in biologically relevant conditions, showing negligible changes in particle size and absorbance in phosphate buffer solution (PBS) and 5% fetal bovine serum within 72 h, as well as unchanged coordination absorption in 5 mm GSH (Figures S20–S22, Supporting Information). All these experimental results collectively demonstrate the successful preparation of FeBPs coordination polymer with well‐defined structural integrity and photo‐responsive functionality.

## 3 Light‐Triggered Acidification and pH‐dependent Coordination Transition

**Figure 2:**
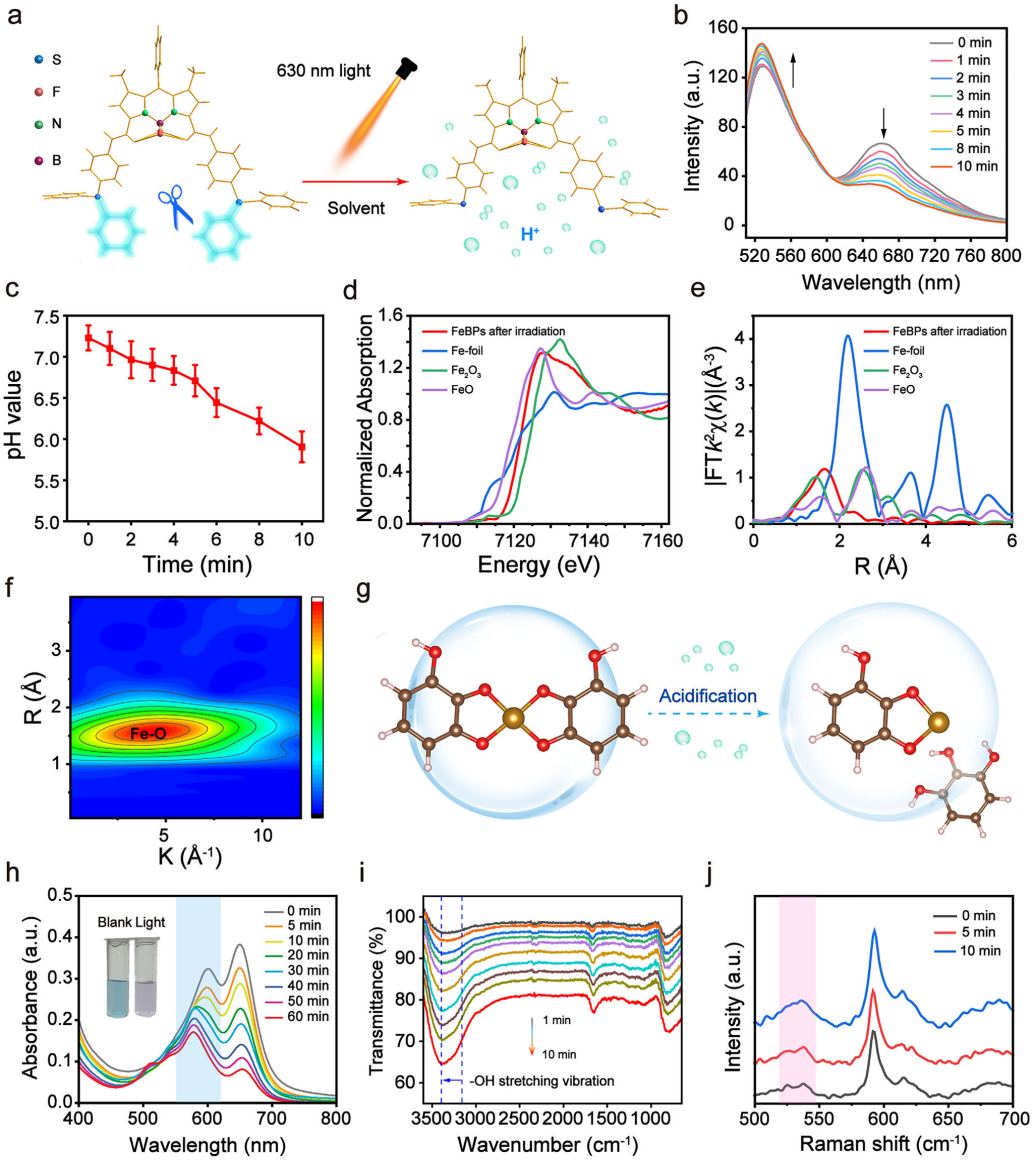
Light‐triggered acidification and coordination transition. a) Schematic diagram of the acid generation mechanism by PAGs‐Bodipy. b) Fluorescence spectra of SNARF‐1 mixed with FeBPs (dispersed in PBS) under 630 nm irradiation (0.33 W cm^─2^) at progressive time intervals. c) pH values calculated from (b) (n = 3). d) Normalized Fe K‐edge XANES of Fe foil, FeO, Fe_2_O_3_, and FeBPs post‐irradiation. e) Fourier transform (R space) of FT‐EXAFS for Fe foil, FeO, Fe_2_O_3_, and FeBPs post‐irradiation. f) WT contour plots of k^2^‐weighted χ(k) signals of FeBPs after irradiation. g) DFT‐calculated pH‐dependent coordination transition. h) UV–vis absorption spectra of FeBPs under 630 nm irradiation. Insets: Solution appearance pre‐ and post‐irradiation. i) In situ FTIR and j) in situ Raman spectra of FeBPs during 630 nm light exposure. Data are means ± SD.

Given the mechanism by which photoacids generate H^+^ upon light irradiation (**Figure** **2**a), the red light (630 nm)‐activated H^+^ generation capability of PAGs‐Bodipy was assessed using rhodamine B (RhB) base as a pH‐sensitive fluorescent probe (Figure S23, Supporting Information).^[^ ^34^ ^]^ Upon 630 nm light irradiation, the absorbance of RhB base at 555 nm increased significantly with exposure time in the prescence of PAGs‐Bodipy, demonstrating effective light‐triggered H^+^ release kinetics (Figure S24, Supporting Information). UV–vis titration further confirmed H^+^ yield gradually rose with extended irradiation time and PAGs‐Bodipy concentrations (Figures S25 and S26, Supporting Information). In addition, HPLC analysis verified this H^+^ release originated from PAGs‐Bodipy photolysis under irradiation (Figure S27, Supporting Information). Crucially, when encapsulated within FeBPs, light‐triggered acidification was further validated by the fluorescence ratiometric response of SNARF‐1 at 560 and 668 nm (Figure 2b; Figure S28, Supporting Information), with pH decrease in buffer solution after 10 min irradiation (Figure 2c).^[^ ^35^ ^]^ This localized pH reduction within FeBPs provided the critical microenvironment for Fe^3+^‐polyphenol coordination changes.

To validate this coordination switch, XAFS analysis was performed on FeBPs after 630 nm irradiation. The normalized Fe K‐edge XANES spectra (Figure 2d) showed a slight shift of Fe absorption edge compared to non‐irradiated FeBPs (Figure 1b), implying subtle valence adjustment driven by light‐triggered acidification. More importantly, FT‐EXAFS and corresponding k‐space EXAFS spectra (Figure 2e; Figure S29, Supporting Information) revealed a distinct change in Fe coordination environment. The average Fe─O coordination number of irradiated FeBPs decreased, indicating the dissociation of partial Fe─O bonds (Table S4, Supporting Information). WT contour plots of k^2^‐weighted χ(k) signals further intuitively confirmed this coordination shift (Figure 2f).^[^ ^36^ ^]^ These results verify that light‐induced H^+^ accumulation disrupts Fe^3+^‐polyphenol coordination. Density functional theory (DFT) calculations further revealed distinct Fe^3+^‐polyphenol coordination geometries across varying pH conditions (Figure 2g).^[^ ^37^ ^]^ Fe‐biscomplexes (FeO4 complexes) exhibit square‐planar configurations under neutral conditions, while increasing acidity drives a pronounced shift toward Fe‐monocomplexes (FeO2 complexes). This transition arises because light‐induced H^+^ accumulation protonates catechol‐OH groups, and this structural change triggers the dissociation of monodentate ligands.^[^ ^38^, ^39^ ^]^ This mechanism is consistent with XAFS‐observed coordination number reduction and clearly links light‐triggered acidification, Fe^3+^‐polyphenol coordination transition, and FeBPs’ microenvironmental regulation.

Multimodal spectroscopy validated this coordination transition.^[^ ^40^, ^41^, ^42^, ^43^ ^]^ A blue‐shifted LMCT band in UV–vis spectra indicated weakened Fe─O coordination (Figure 2h). In situ FTIR analysis revealed that under 630 nm light irradiation, the intensity of the ─OH stretching vibration absorption peak at 3400 cm^−1^ was significantly enhanced and accompanied by a blue shift (Figure 2i), attributed to the stepwise protonation of polyphenol hydroxyl groups triggered by light‐induced acidification.^[^ ^44^, ^45^ ^]^ The enhancement of ─OH signal is directly related to the cleavage of the Fe─O coordination bond, as the protonation of the hydroxyl group reduces the electron‐donating capacity of polyphenol ligands, thereby weakening their binding to Fe active sites.^[^ ^46^, ^47^ ^]^ This effect facilitates the accelerated release of Fe active sites, thereby enhancing the efficiency of the subsequent Fenton reaction. In situ Raman spectra (500–550 cm^−1^) reveal pH‐dependent peak merging. Two sharp bands at ≈525 and ≈540 cm^−1^ coalesce into a single broad feature under 630 nm irradiation, reflecting light‐triggered acidification‐driven weakening of iron‐phenolate coordination, which aligns with our DFT‐calculated mechanism (Figure 2j).^[^ ^39^, ^48^ ^]^ Collectively, the integration of light‐triggered acidification and pH‐driven coordination transition enables programmable metal‐ligand dissociation. This light‐triggered coordination destabilization establishes a structural pre‐activation state in FeBPs, wherein ligand dissociation primes Fe active centers for redox‐state modulation and coordination‐amplified Fenton reactivity.

## 4 Light‐Driven Coordination Transition Accelerates ROS Generation

**Figure 3:**
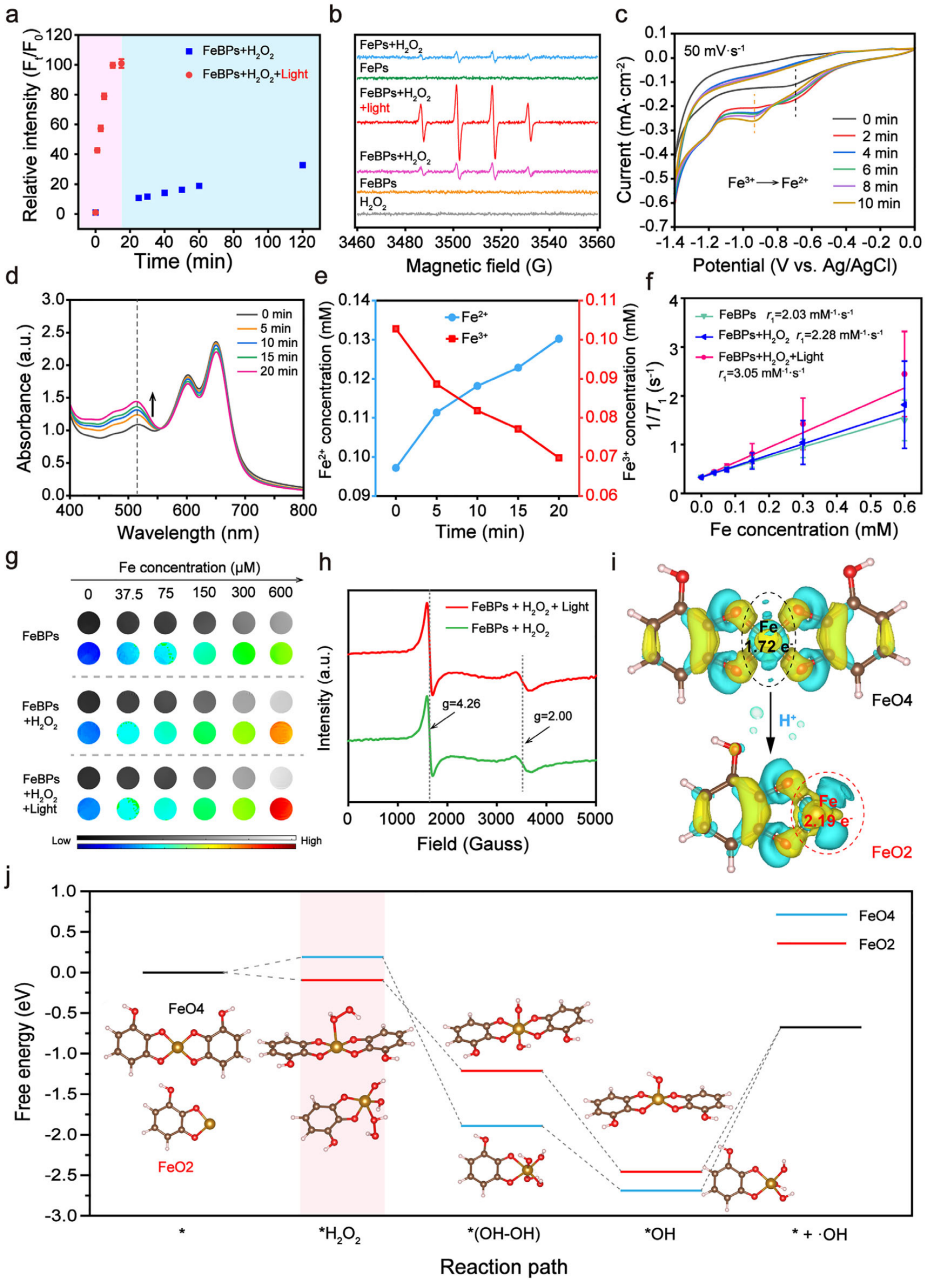
Light‐driven coordination transition accelerates ROS generation. a) Quantitative ROS detection via coumarin fluorescence under different treatments. The concentration of H_2_O_2_ is 100 µm. b) ESR spectra of DMPO‐•OH adduct with various treatments. c) Cyclic voltammetry of FeBPs with and without light irradiation. d) Time‐dependent iron speciation analysis through 1,10‐phenanthroline complexation (630 nm, 0.33 W cm^−2^). e) Quantification of Fe^2+^/Fe^3+^ changes during light irradiation (n = 3). f) *r*_1_ relaxivity and g) *T*_1_‐weighted MRI image of FeBPs under different conditions (n = 3). h) H_2_O_2_‐dependent ESR spectra of FeBPs with/without light irradiation. i) Isosurfaces (level 0.0035) of charge density differences on FeBPs with FeO4 and FeO2 configurations. Yellow indicates electron accumulation. j) DFT‐calculated activation energy landscape for H_2_O_2_ homolysis across coordination states. Data are means ± SD.

To validate the effect of light‐driven coordination transition on ROS generation, kinetic assays and spectroscopic characterizations were performed. Under 630 nm irradiation, FeBPs exhibited accelerated •OH production kinetics in the presence of H_2_O_2_, as evidenced by coumarin fluorescence reaching saturation within 10 min (**Figure** **3**a; Figure S30, Supporting Information).^[^ ^49^ ^]^ A concentration‐dependent relationship between fluorescence intensity and both H_2_O_2_/FeBPs levels further confirmed ROS amplification (Figure S31, Supporting Information). Notably, light‐triggered H^+^ release synergized with transient coordination fluctuations to accelerate •OH generation kinetics in the FeBPs + H_2_O_2_ + Light group by ≈10‐fold compared to FeBPs + H_2_O_2_ group. Electron spin resonance (ESR) spectra further validated this enhancement. The 5,5‐dimethyl‐1‐pyrroline N‐oxide (DMPO) •OH adduct intensity (characteristic 1:2:2:1 quartet) was prominently increased in the FeBPs + H_2_O_2_ + Light group relative to the non‐irradiated FeBPs + H_2_O_2_ group, while ^1^O_2_ signals were relatively low (Figure 3b; Figure S32, Supporting Information). These findings confirm that photoacid‐induced coordination transition boosts Fenton‐mediated •OH generation rather than ^1^O_2_ production under light irradiation.

In order to exhibit light‐modulated Fe^2+^ availability for sustained Fenton catalysis, a thorough mechanistic investigation was conducted. Cyclic voltammetry (CV) was performed following established protocols,^[^ ^50^ ^]^ with K_4_[Fe(CN)_6_]/K_3_[Fe(CN)_6_] as reference standards to calibrate redox potentials (Figure S33, Supporting Information). CV curves exhibited a significant cathodic shift of the reduction peak (≈250 mV) relative to the reference, corresponding to a more negative potential that indicates an increased overpotential required for Fe^3+^ reduction. This shift is attributed to photo‐induced H^+^ generation, which weakens Fe─O coordination bonds (Figure 3c). When irradiated with 630 nm light for varying durations, the reduction peak current of FeBPs increased notably after 10 min of exposure. This result confirms that prolonged irradiation elevates H^+^ concentration, which further promotes Fe─O bond dissociation and subsequent release of Fe^2+^. Additionally, for FeP (incapable of photo‐induced H^+^ generation), the CV curve of FeP with added exogenous H^+^ showed a distinct cathodic shift in the reduction peak, clearly demonstrating that H^+^ directly facilitates Fe^3+^→Fe^2+^ conversion by weakening Fe─O coordination.^[^ ^51^ ^]^ Notably, thermal control experiments confirmed no detectable heating effects under irradiation (ΔT < 0.5 °C; Figure S34, Supporting Information), excluding potential photothermal interference. UV–vis spectra with 1,10‐phenanthroline (a Fe^2+^‐specific chelator) quantification further confirmed rapid light‐driven Fe^2+^ accumulation, accompanied by proportional Fe^3+^ depletion in FeBPs under irradiation, which validates the CV‐derived conclusion that H^+^‐induces weakening of Fe─O coordination and subsequent Fe^2+^ release (Figure 3d,e; Figure S35, Supporting Information).

Magnetic resonance imaging (MRI) provided real‐time validation of Fe valence state change through longitudinal relaxivity (*r*_1_) enhancement mediated by the low‐spin d6 Fe^2+^ to high‐spin d5 Fe^3+^ transition.^[^ ^52^ ^]^ *T*_1_‐weighted signal attenuation post‐irradiation indicated high‐spin Fe^3+^ conversion to low‐spin Fe^2+^ conversion (Figure S36, Supporting Information). H_2_O_2_ addition increased *r*_1_ from 2.03 to 2.28 mm ^−1^ s^−1^, amplified to 3.05 mm ^−1^ s^−1^ upon light irradiation (Figure 3f), reflecting accelerated Fenton efficiency. In contrast, FeP controls (lacking photoacid moieties) showed negligible relaxivity changes with light, confirming acidification‐coordination coupling specificity (Figure S37, Supporting Information). Notably, pronounced MRI signal intensification directly correlated with localized Fe^3+^ accumulation, serving as a spatiotemporal visualization of Fenton efficiency (Figure 3g). ESR spectra further confirmed the evolution of iron spin state during the Fenton cycle (Figure 3h). Compared to FeBPs + H_2_O_2_, the FeBPs + H_2_O_2_ + Light system exhibited intensified Fe^3+^ signals at g = 4.26 and 2.02, indicating progressive activation of catalytic sites under irradiation.^[^ ^53^ ^]^ Notably, FeP showed no significant changes between light and dark conditions (Figure S38, Supporting Information). Collectively, these data establish the mechanism that light‐triggered acidification and coordination fluctuations promotes iron reduction in FeBPs while enhancing metal center accessibility for H_2_O_2_ activation, enabling ROS amplification.

To further investigate this mechanism, DFT calculations were performed to compare electron density and H_2_O_2_ activation energetics across distinct coordination states. Electron density difference analysis reveals that photoacidification‐induced ligand dissociation increases electron density at the iron center, enhancing H_2_O_2_ adsorption at exposed active sites (Figure 3i). FeO2 monocomplexes (ΔG = −0.10 eV) exhibits lower activation barrier for H_2_O_2_ adsorption than the FeO4 biscomplexes (ΔG = 0.19 eV), with its rate‐determining step requiring less energy than FeO4 (Figure 3j). This facilitates greater •OH generation within equivalent timeframes, consistent with accelerated ROS production under 630 nm irradiation. Collectively, these results demonstrate a photo‐proton‐coordination coupling mechanism for ROS amplification in FeBPs: Light‐induced pH reduction drives the FeO4→FeO2 transition via charge transfer. The weakened coordination and optimized electronic configuration reduce the Fenton reaction activation barrier. Mechanistically, Fe species undergo deprotonation in acidic environments to form the FeO_2_(OH)_2_^*^ intermediate, where subsequent charge transfer and Fe─O bond cleavage yield •OH. This synchronized photo‐proton‐coordination coupling enables efficient and sustained ROS generation.

## 5 Biocompatibility, Cellular Uptake, and Mitochondrial Localization

**Figure 4:**
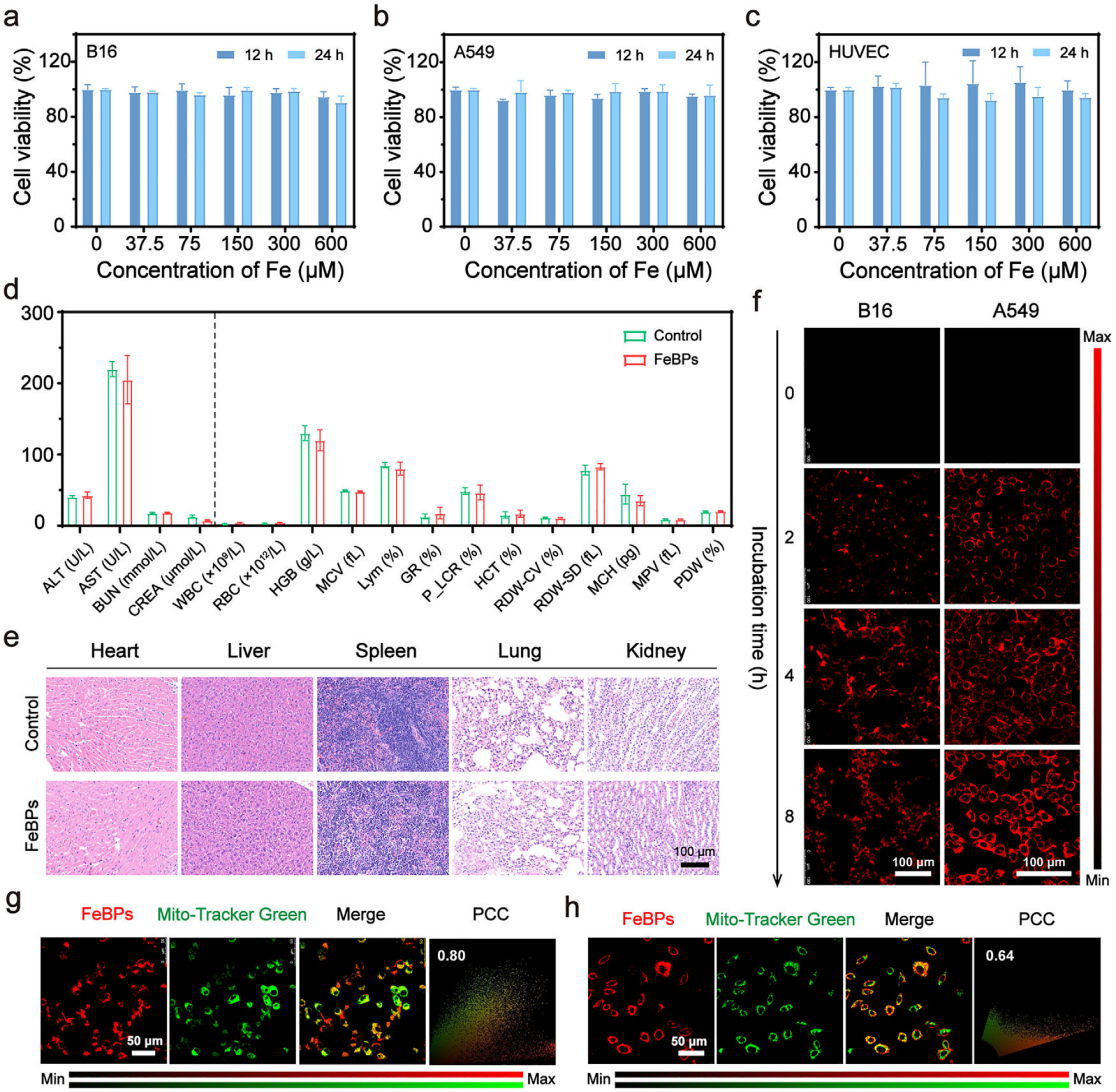
Biocompatibility, cellular uptake, and mitochondrial localization of FeBPs. a) Cell viability of B16‐F10 cells, b) A549 cells, and c) HUVEC cells after 12 and 24 h incubation with FeBPs (n = 5). d) Biochemical indexes and blood routine of mice (n = 3) and e) Representative H&E‐stained histopathological sections of major organs in mice 24 h post‐intravenous injection of FeBPs. Scale bar: 100 µm. f) CLSM images of time‐dependent cellular uptake of FeBPs in B16‐F10 and A549 cells. Scale bar: 100 µm. g) Colocalization analysis between FeBPs (red) and MitoTracker Green in B16‐F10 cells and h) A549 cells. Scale bar: 50 µm. Data are means ± SD.

Building upon the well‐defined physicochemical properties of FeBPs, we systematically evaluated their biosafety profiles as a prerequisite for biomedical applications. Cell Counting Kit‐8 assays demonstrated that FeBPs maintained over 85% cell viability across three representative cell lines: mouse melanoma cells (B16‐F10), human non‐small cell lung cancer cells (A549), and human umbilical vein endothelial cells (HUVECs) at concentrations up to 600 µm (**Figure** **4**a–c), indicating the negligible cytotoxicity of FeBPs. Systemic toxicity assessment in mice revealed no significant alterations in hepatic/renal biomarkers compared to controls, including alanine aminotransferase (ALT), aspartate aminotransferase (AST), blood urea nitrogen (BUN), creatinine (CREA), and hematological parameters (Figure 4d). Histopathological examination of major organs (heart, liver, spleen, lung, and kidney) via hematoxylin and eosin (H&E) staining further confirmed the absence of tissue damage in FeBPs‐treated mice (Figure 4e), while Prussian blue staining showed minimal iron accumulation in these organs at 24 h post‐injection (Figure S39, Supporting Information). These multiscale biocompatibility data support FeBPs’ potential for bio‐applications.

In addition, capitalizing on the intrinsic fluorescence of PAGs‐Bodipy components (λ_ex_ = 633 nm), the cellular internalization kinetics of FeBPs was monitored by confocal laser scanning microscopy (CLSM). Quantitative analysis revealed time‐dependent cellular uptake in both B16‐F10 and A549 cells (Figure 4f), a pattern corroborated by Prussian blue staining showing maximal intracellular iron deposition at 8 h incubation (Figure S40, Supporting Information). The tertiary sulfonium moiety in FeBPs’ PAGs‐Bodipy contributes to both cellular internalization and mitochondrial targeting, which confers a positive surface charge (Figure S17, Supporting Information) to promote cell membrane adsorption and endocytosis, and further enables selective mitochondrial accumulation post‐internalization.^[^ ^54^, ^55^ ^]^ This mitochondrial localization was validated by colocalization analysis with MitoTracker Green. High Pearson's coefficients (PCC) significantly exceeded random distribution thresholds (0.1–0.3),^[^ ^56^ ^]^ demonstrating spatial overlap between FeBPs and mitochondria (Figure 4g,h). These data collectively link FeBPs’ structural feature (tertiary sulfonium moiety) to both cellular internalization and mitochondrial‐specific localization, while maintaining biosafety critical for therapeutic applications.

## 6 Light‐Triggered Intracellular Acidification and ROS Amplification In Vivo

**Figure 5:**
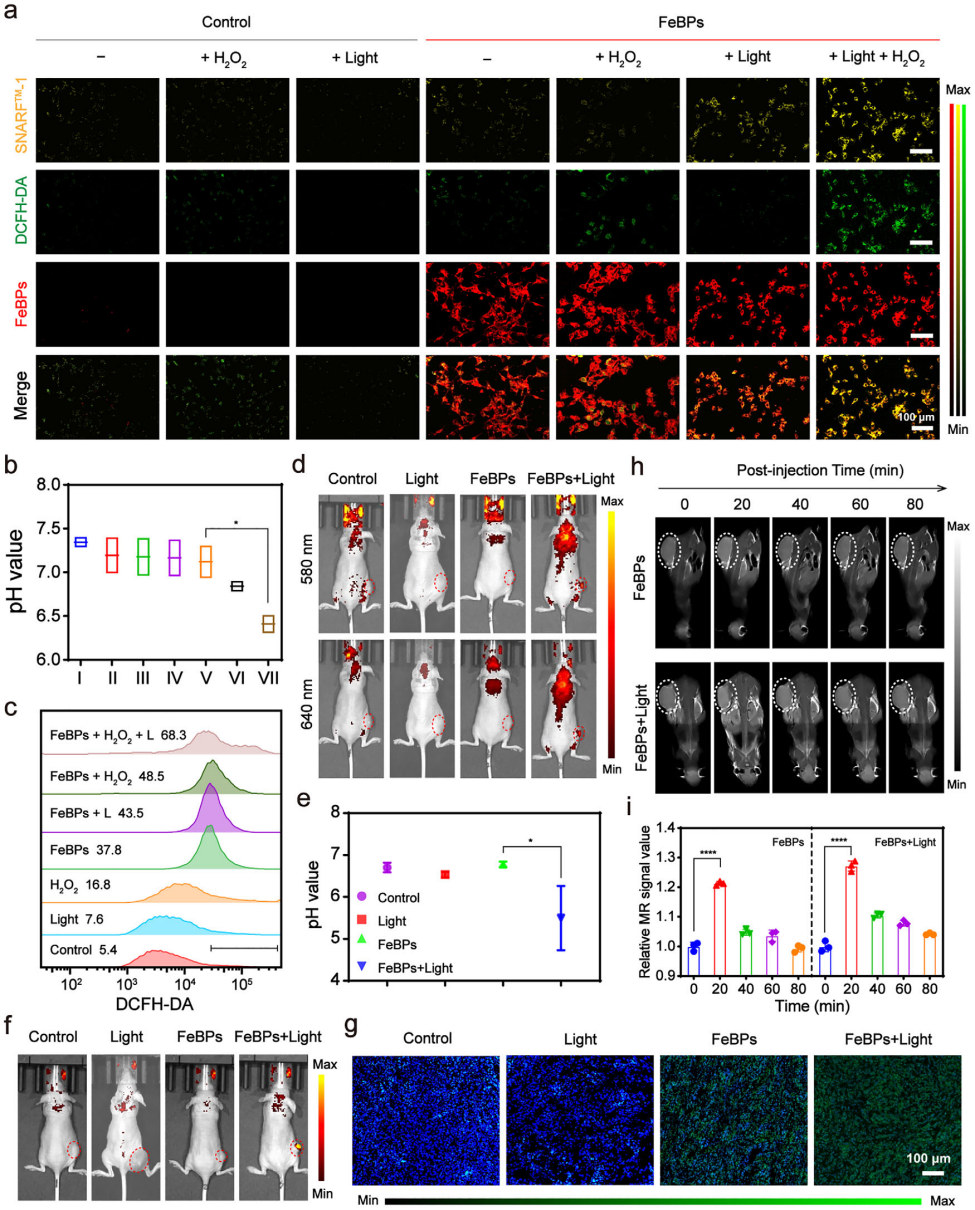
Light‐triggered intracellular acidification and ROS amplification. a) Confocal fluorescence micrographs of B16‐F10 cells stained with SNARF‐1 (λ_ex_/λ_em_: 488/580 nm) and DCFH‐DA (•OH probe, λ_ex_/λ_em_: 488/525 nm) after various treatments. The concentration of H_2_O_2_ is 100 µm. Scale bar: 100 µm. b) Quantitative pH of the cell suspension calculated from SNARF‐1 dual‐emission ratios (580 nm/640 nm). I: Control, II: H_2_O_2_, III: Light, IV: FeBPs, V: FeBPs + H_2_O_2_, VI: FeBPs + Light, VII: FeBPs + H_2_O_2_ + Light. (^*^ *p* < 0.05). c) Flow cytometry quantification of ROS expression levels (DCFH‐DA) in cells after different treatments. d) In vivo fluorescence imaging of tumor‐bearing mice labeled by SNARF‐1 ratiometric probe (tumor region circled with red dash). e) Tumor pH quantification derived from (d) by ratiometric 580/640 nm analysis (^*^ *p* < 0.05 vs FeBPs controls). f) In vivo DCFH‐DA fluorescence imaging of tumor‐bearing mice (tumor region circled with red dash, n = 3). g) Immunofluorescence staining of ex vivo DCFH‐DA‐stained tumor sections. Scale bar: 100 µm. h) *T*_1_‐weighted MR images (1.0 T) 24 h post‐injection of FeBPs (0.2 mg [Fe] kg^−1^) ± light irradiation (white dashed: tumor ROI). i) Relative MR signal intensity of (h). (^****^ *p* < 0.0001 vs initial time). Data are means ± SD (n = 3). Statistical analysis was performed using unpaired *t*‐test.

Inspired by the well performance of photoacids‐enhanced Fenton efficacy, the levels of photo‐generated H^+^ and the promotion of ROS by FeBPs were further investigated across cellular and tumor‐bearing mice. Initial assessment using the pH‐sensitive probe BCECF‐AM revealed significant fluorescence quenching in both B16‐F10 and A549 cells treated with FeBPs under 630 nm light irradiation, particularly in the FeBPs + H_2_O_2_ + Light groups (Figure S41, Supporting Information), confirming light‐triggered intracellular acidification. To establish the causal relationship between photo‐generated H^+^ and ROS amplification, dual‐probe imaging was implemented using SNARF‐1, a ratiometric pH probe exhibiting pH‐dependent wavelength shift, in conjunction with DCFH‐DA, a •OH‐responsive probe that generates green fluorescence at 525 nm emission wavelength upon oxidative activation. There is an obvious spatial‐temporal coupling between acidified regions (indicated by SNARF‐1 signal attenuation, yellow) and ROS hotspots (shown by DCFH‐DA fluorescence intensification, green) in cells treated with FeBPs + H_2_O_2_ + Light (Figure 5a; Figure S42, Supporting Information). Quantitative ratiometric analysis of SNARF‐1 fluorescence showed a notable localized pH decrease within B16‐F10 cells treated with FeBPs upon 630 nm irradiation (Figure 5b), paralleled by a ROS elevation via flow cytometry (DCFH‐DA positive cells: 5.4% to 68.3%, Figure 5c). These data mechanistically demonstrate that 630 nm light‐triggered H^+^ generation, resulting in localized intracellular acidification that exposes FeBPs’ active sites, thereby accelerating Fenton reaction kinetics and enhance intracellular ROS levels.

To validate light‐triggered H^+^ release from FeBPs in vivo, SNARF‐1 ratiometric imaging (580 nm/640 nm) was employed to monitor intratumoral pH dynamics in tumor‐bearing mice (Figure 5d). Upon 630 nm irradiation for 10 min, the fluorescence intensity ratio decreased, corresponding to rapid pH reduction from 6.8 to 5.5 via standard curve calibration (Figure 5e; Figure S43, Supporting Information). Concurrently, DCFH‐DA fluorescence imaging revealed a 6.8‐fold ROS elevation in light irradiated tumors (Figure 5f; Figure S44, Supporting Information). Ex vivo analyses of tumor sections also showed obvious DCFH‐DA fluorescence in FeBPs + Light treated tumor (Figure 5g), and 4‐hydroxynonenal (4‐HNE) immunohistochemistry with diaminobenzidine (DAB) staining exhibited partial brown positive staining, which evidenced lipid peroxidation (Figure S45, Supporting Information), indicating sustained ROS generation.^[^ ^57^ ^]^ These consistent in vivo and ex vivo results collectively link photoacid‐driven intratumoral pH drop to enhanced Fenton reactivity under tumor‐specific H_2_O_2_ overexpression, confirming light‐triggered oxidative stress amplification in FeBPs‐treated tumors.

The paramagnetic properties of FeBPs enabled real‐time monitoring of ROS dynamics through valence‐dependent *T*_1_‐weighted MRI. Intravenous administration of FeBPs induced progressive tumor *T*_1_ signal enhancement, peaking at 20 min post‐injection (Figure 5h; Figure S46, Supporting Information). Notably, 630 nm light irradiation significantly amplified this effect, leading to a higher relative signal intensity compared to non‐irradiated controls (Figure 5i; Figure S47, Supporting Information). This enhancement can be attributed to the photoacid‐driven acceleration of ROS generation. In addition, the main metabolic organs, including kidneys and liver, displayed strong enhanced *T*_1_‐weighted MRI after intravenous injection, and rapid signal enhancement in kidneys and livers within 20 min post‐injection, followed by signal decay after 60 min, indicating concurrent renal filtration and hepatic metabolism of FeBPs (Figure S48, Supporting Information). These results collectively demonstrate that light‐controlled acidification effectively enhances ROS generation while ensuring favorable metabolic clearance.

## 7 Photoacidification Modulates ROS Amplifications for Effective Tumor Therapy

**Figure 6:**
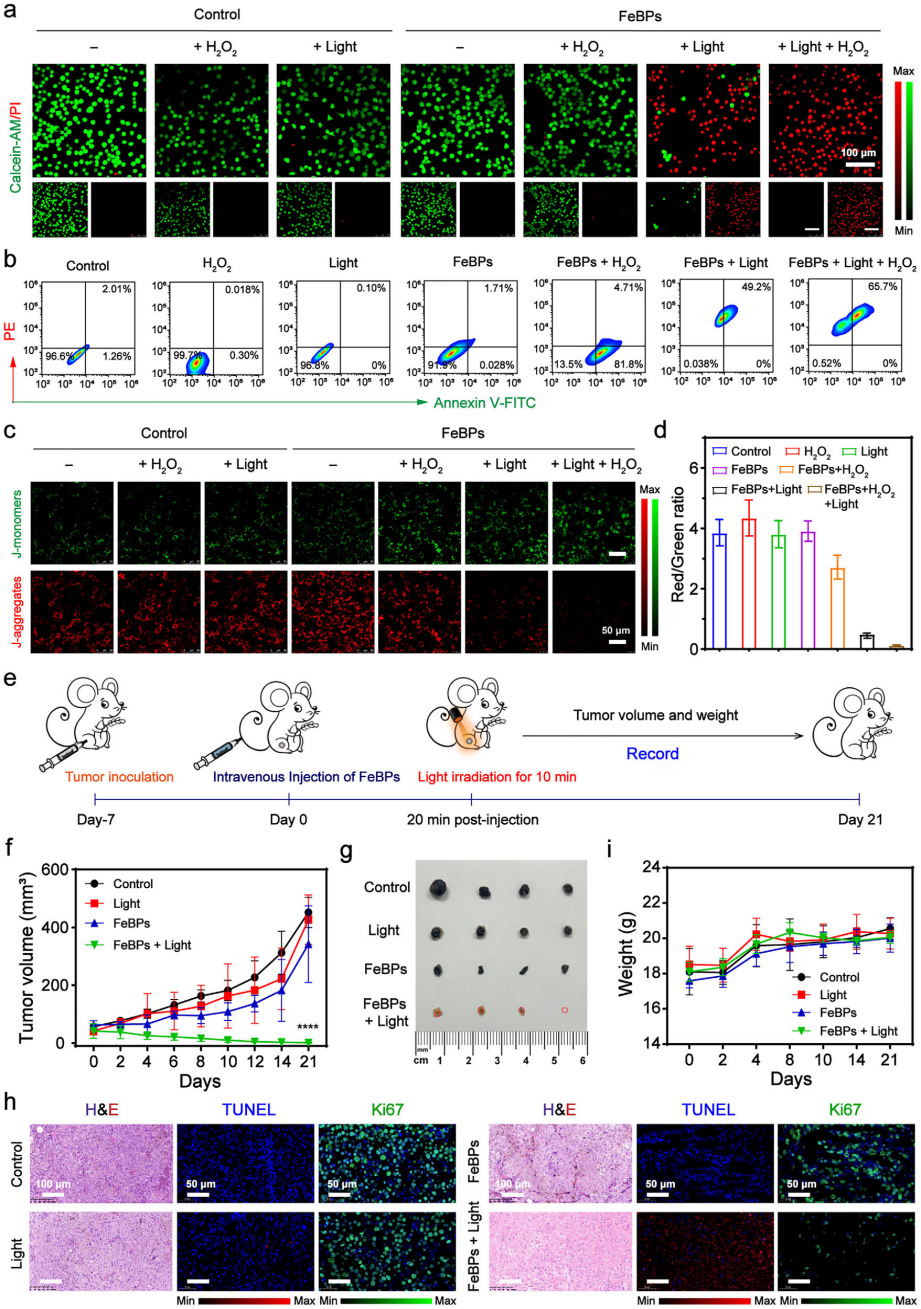
Photoacidification modulates ROS amplifications for tumor therapy. a) Confocal fluorescence images of B16‐F10 cells after different treatments and stained with Calcein‐AM (viable cells, green; λ_ex_/λ_em_ = 488/515 nm) and PI (dead cells, red; λ_ex_/λ_em_ = 535/617 nm). The concentration of H_2_O_2_ is 100 µm. Scale bar: 100 µm. b) Apoptosis profiles analyzed by Annexin V‐FITC/PI flow cytometry. c) JC‐1 fluorescence imaging showing mitochondrial aggregates (red; λ_ex_/λ_em_ = 585/590 nm) and monomers (green; λ_ex_/λ_em_ = 514/529 nm). Scale bar: 50 µm. d) Changes in cell mitochondrial membrane potential calculated by the ratio of JC‐1 aggregate‐to‐monomer fluorescence intensity (n = 3). e) Schematic of tumor implantation and treatment timeline. f) Tumor volume changes of mice over 21 days (n = 4). g) Typical tumor images at endpoint (Day 21). h) Typical staining of tumor sections with various treatments, including H&E, TUNEL, and Ki67. Scale bars: 100 µm (H&E), 50 µm (TUNEL and Ki67). i) Body weight variations of mice during treatment (n = 4). Data are means ± SD. Tumor volume statistical analysis was performed using two‐way analysis of variance. ^****^ *p* < 0.0001.

The synergistic effects of light‐triggered acidogenesis and ROS amplification on tumor cell apoptosis and mitochondrial dysfunction were systematically investigated. Live/dead dual staining by Calcein‐AM (green) and propidium iodide (PI, red) revealed photoacidification‐dependent cytotoxicity (**Figure** **6**a; Figure S49, Supporting Information). Control groups (untreated, light‐only, H_2_O_2_‐only, FeBPs‐only) maintained cells viability as evidenced by predominant green fluorescence, while FeBPs + H_2_O_2_ induced partial cell death with reduced green signal intensity due to ROS generation. Strikingly, 630 nm irradiation triggered drastic cytotoxicity in FeBPs + Light and FeBPs + H_2_O_2_ + Light groups. Flow cytometric quantification of Annexin V‐FITC/PI staining confirmed apoptosis induction, showing 65.7% apoptotic cells in FeBPs + H_2_O_2_ + Light group (Figure 6b). This implies that light‐triggered H^+^ release promotes intracellular acidification and enhances ROS generation efficacy, thereby inducing apoptosis. Mitochondrial destabilization under light activation was further demonstrated using the JC‐1 membrane potential probe. Following 630 nm irradiation, JC‐1 aggregates (red) decreased while monomers (green) increased (Figure 6c). The aggregate‐to‐monomer fluorescence ratio exhibited a significant reduction compared to controls, indicating ΔΨ_m_ collapse–a hallmark of early apoptosis (Figure 6d). This phenomenon originates from triarylsulfonium‐based PAGs producing substantial H^+^, which disrupts mitochondrial acid‐base homeostasis and amplifies Fenton reactivity, ultimately driving sustained •OH production and tumor cell apoptosis.

Therapeutic efficacy was further validated in B16‐F10 tumor‐bearing mice randomized into four core groups (Control, Light, FeBPs, FeBPs + Light), with an additional FeP group (Fe‐based alone) to exclude photoacid contributions (Figure 6e). The FeBPs + Light group achieved complete tumor suppression with no regrowth over 21 days, while FeBPs alone exhibited limited tumor inhibition (Figure 6f,g; Figure S50, Supporting Information), likely due to poor intrinsic Fenton activity. Similarly, the FeP group (with an identical Fe content to FeBPs) also showed weak anti‐tumor effects over 14 days of monitoring (Figure S51, Supporting Information). This observation confirms that Fe‐based materials alone cannot drive effective suppression, as they lack photoacid‐triggered H^+^ generation—a key step for boosting Fenton reactivity, which further highlights the indispensable role of photoacid components in FeBPs. Histopathological analysis corroborated treatment specificity. H&E staining revealed extensive tumor cell damage, and terminal deoxynucleotidyl transferase uridine triphosphate nick end labeling (TUNEL) staining showed a significant increase in apoptotic cells in the FeBPs + Light group compared to controls (Figure 6h), confirming apoptosis as the primary cell death pathway. Additionally, Ki‐67 immunostaining demonstrated significantly reduced proliferative activity of tumor cells, and no significant glutathione peroxidase 4 (GPX4) expression differences across groups ruled out ferroptosis as a dominant mechanism (Figure S52, Supporting Information). In addition, systemic toxicity remained undetectable, as indicated by stable body weight (Figure 6i). These results conclusively establish that photoacid‐driven intratumoral acidification acts as a biochemical amplifier by coupling H^+^‐mediated Fenton potentiation with sustained ROS elevation, thereby achieving effective tumor eradication through oxidative stress amplification. Notably, compared to NIR‐II wavelengths, 630 nm light still has relatively limited tissue penetration depth, and further exploration is needed to expand its applicability, especially for deep tumors.

## 8 Conclusion

In summary, we have developed a light‐responsive FeBPs coordination polymer through self‐assembly of Fe‐polyphenol‐PEG via metal‐ligand coordination, and photoacid generators (Bodipy derivatives). Upon 630 nm irradiation, FeBPs exhibit spatiotemporally controlled H^+^ release, photoacidification‐triggered coordination transition facilitates the exposure of active center, while sustains Fenton catalytic efficiency under acidic conditions to boost •OH generation rate. This synergistic interplay between metal‐ligand coordination change and catalytic microenvironment optimization enables tumor‐specific ROS storms, which collapse mitochondrial membrane potential and activate cell apoptosis. In murine melanoma models, localized light‐triggered H^+^ generation and ROS dual attack achieve complete tumor regression with negligible systemic toxicity. The proposed coordination engineering strategy overcomes traditional limitations of iron‐based therapeutics, including insufficient active center exposure and pH‐restricted catalytic activity, establishing a paradigm for developing adaptive metal‐phenolic theranostics.

## Conflict of Interest

The authors declare no conflict of interest.

## Supporting information



Supporting Information

## Data Availability

The data that support the findings of this study are available from the corresponding author upon reasonable request.
