# Supplementary material for: Acidogenesis‐Propelled Coordination Transition in Light‐Triggered Fe‐Polyphenol Polymer for Reactive Oxygen Species‐Augmented Antitumor Therapy
Source: Adv Sci (Weinh). 2025 Sep 27;12(47):e13361. doi: 10.1002/advs.202513361 (PMC12713086; doi:10.1002/advs.202513361)
Supplement: Supplementary file 1 — Supporting Information [file ADVS-12-e13361-s001.docx]

Supporting Information

Acidogenesis-Propelled Coordination Transition in Light-Triggered Fe-Polyphenol Polymer for Reactive Oxygen Species-Augmented Antitumor Therapy

Ying Wan, Hui Liu, Lin Gao, Guanyu Tan, Kailin Li, Qiwei Tian^*^, Shiping Yang^*^, Lu An^*^

Y. Wan, H. Liu, L. Gao, G. Y. Tan, K. L. Li, S. P. Yang, L. An

The Education Ministry Key Lab of Resource Chemistry, Shanghai Municipal Education Committee Key Laboratory of Molecular Imaging Probes and Sensors, Shanghai Key Laboratory of Rare Earth Functional Materials, Shanghai Normal University, Shanghai 200234, China

E-mail: [anlu1987@shnu.edu.cn,](mailto:anlu1987@shnu.edu.cn,) shipingy@shnu.edu.cn

Y. Wan, Q.W. Tian

Shanghai Key Laboratory of Molecular Imaging, Jiading District Central Hospital Affiliated Shanghai University of Medicine and Health Sciences, Shanghai University of Medicine and Health Sciences, Shanghai 201318, China

E-mail: tianqw@sumhs.edu.cn

**Table of Contents**

[1. Chemicals and materials 3](#_Toc24639)

[2. Characterization 3](#_Toc31822)

[3. Synthesis routes of desired materials 4](#_Toc26045)

[4. Photophysical properties of PAGs-Bodipy 9](#_Toc4039)

[5. H](#_Toc16663)^[+](#_Toc16663)^ [generation](#_Toc16663) *[in vitro](#_Toc16663)* [10](#_Toc16663)

[6. •OH production](#_Toc14221) *[in vitro](#_Toc14221)* [10](#_Toc14221)

[7. Oxidation reduction potential 11](#_Toc10988)

[8. Valence changes of Fe 11](#_Toc10938)

[9. XAFS analysis 11](#_Toc17152)

[10. DFT calculation 12](#_Toc717)

[11. MRI performance](#_Toc31353) *[in vitro](#_Toc31353)* [12](#_Toc31353)

[12. Cell culture and cell viability assay 13](#_Toc15217)

[13. Cellular uptake and colocalization 13](#_Toc22790)

[14. 630 nm light-triggered intracellular acidification and ROS generation 14](#_Toc5501)

[15. Mitochondrial membrane potential 14](#_Toc15461)

[16. Cell apoptosis 14](#_Toc7926)

[17. Biosafety of FeBPs](#_Toc27866) *[in vivo](#_Toc27866)* [15](#_Toc27866)

[18. Acidification in tumors 15](#_Toc28695)

[19. ROS generation in tumors 15](#_Toc30356)

[20.](#_Toc28080) *[T](#_Toc28080)*_[1](#_Toc28080)_ [MRI](#_Toc28080) *[in vivo](#_Toc28080)* [16](#_Toc28080)

[21. Inhibition of tumor growth 16](#_Toc30489)

[22. Data analysis 16](#_Toc9043)

[23. Statistical analysis 17](#_Toc18222)

[24. Supplementary Tables and Figures 18](#_Toc7220)

[25. References 74](#_Toc27857)

**1. Chemicals and materials**

Gallic acid (GA, 3,4,5-trihydroxybenzoic acid), tert-butyldimethylchlorosilane (TBSCl), N,N-diisopropylethylamine (DIEA), acetic acid (AcOH), ethyl acetate (EA), sodium bicarbonate (NaHCO_3_), sodium chloride (NaCl), sodium sulfate (Na_2_SO_4_), tetrahydrofuran (THF), 4-dimethylaminopyridine (DMAP), methanol (CH_3_OH), dimethylformamide (DMF), p-toluenesulfonic acid monohydrate (TsOH), hydroquinone, dichloromethane (CH_2_Cl_2_), diisopropylimidazole (DIPC), petroleum ether (PE), pyridine (Py), pyridine hydrofluoric acid (HF·py), copper sulfate pentahydrate (CuSO_4_·5H_2_O), anhydrous magnesium sulfate (MgSO_4_), potassium carbonate (K_2_CO_3_), copper(I) bromide (CuBr), pentamethyldiethylenetriamine (PMDETA), ferric chloride hexahydrate (FeCl_3_·6H_2_O), diphenyl iodonium hexafluorophosphate, copper acetate, chlorobenzene, piperidine, acetonitrile, and 4 Å molecular sieves, all of which were provided by Admas Reagent Co., Ltd. 1,3,5,7-tetramethyl-8-phenyl-4,4-difluoro-1,4-diazabicyclo[2.2.2]octane provided by Aladdin Reagent Co., Ltd., 4-(phenylthio)benzaldehyde provided by Shanghai Bide Pharmatech Co., Ltd., and N_3_-PEG (M_w_ = 2000) was provided by Sigma-Aldrich Reagent Co., Ltd. All the reagents mentioned above were used directly without further purification.

SNARF^TM^-1 pH fluorescence probe provided by Shanghai Merie Biochemical Technology Co., Ltd., BCECF-AM pH fluorescence probe provided by Tongren Chemical Co., and DCFH-DA reactive oxygen species detection kit, Mito-Tracker Green (Mitochondria-specific green fluorescent probes), mitochondrial membrane potential detection kit (JC-1), Calcein-AM/PI cell viability and cytotoxicity detection kit, Annexin V-FITC Apoptosis Detection Kit, all of which were provided by Shanghai Beyotime Biotechnology Co., Ltd.

**2. Characterization**

^1^H NMR spectra were recorded using a 400 MHz NMR spectrometer, with ^1^H shifts for CDCl_3_ and DMSO-*d*_6_ observed at 7.26 ppm and 2.50 ppm, respectively. ^13^C NMR spectra were recorded using a 101 MHz NMR spectrometer, with ^13^C shifts for CDCl_3_ and *d*_6_-DMSO observed at 77.1 ppm and 39.6 ppm, respectively. Fluorescence spectra were collected using the fluorescence spectrometer (FLS 980, EDNIBURCH INSTRUMENT). Transmission electron microscopy (JEM-2010, JEOL) was used to determine the morphology and elements mapping of FeBPs assembly. The structure of FeBPs were determined by Fourier-transform infrared (FT-IR) spectroscopy (Nicolet Avatar 370, Thermo), Raman spectroscopy (Jobin Yvon confocal laser Raman system, SuperLabRam II), and Shimadzu UV-1900i spectrophotometer. In situ Raman studies were performed by a HORIBA HR Evolution Raman spectrometer equipped with a 532 nm YAG solid-state laser with a 100 µm slit, 5 s exposure time and 3-5 cycles. In-situ FTIR spectroscopic studies were performed using a NICOLET iS50 FT-IR spectrometer equipped with an MCT/A detector. The g values of the materials were determined by electron paramagnetic resonance (EPR, Bruker EMXPLUS) spectroscopy. Hydrodynamic diameter and zeta potential were performed by laser particle size analyzer (Malvern Nano ZS, UK). Iron-ion concentrations were measured by high-dispersion inductively coupled plasma atomic emission spectroscopy (ICP-OES, VISTAMPXICP Varian).

**3. Synthesis routes of desired materials**

**3.1 Synthesis of PAGs-Bodipy**


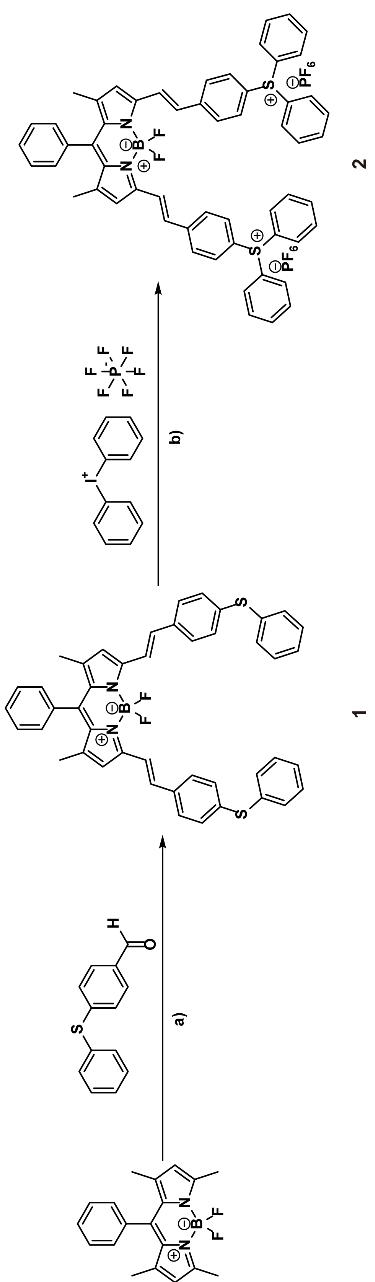


**Scheme S1.** Preparation of PAGs-Bodipy. a) 4 Å molecular sieves, glacial acetic acid, piperidine, dry acetonitrile, N_2_, 85 ^o^C, 48 h. b) pyridine, THF, pyridine hydrofluoride, r.t., 2 h.

Synthesis of **compound 1**: This compound was synthesized according to a modified literature method^[1]^. 3,5,7-Tetramethyl-8-phenyl-4,4-difluoroboradiazaindacene (Bodipy dye, 0.5 mmol) was reacted with 4-(phenylthio)benzaldehyde (2.5 mmol) in a solvent mixture comprising dry acetonitrile (20 mL), piperidine (1.0 mL), glacial acetic acid (0.6 mL), and 4Å molecular sieves (500 mg) under a nitrogen atmosphere (N_2_). The reaction was conducted under reflux at 85 ^o^C for a period of 48 hours. Post-reaction, the solvent was removed under reduced pressure. The resulting crude product was subjected to purification via column chromatography on silica gel, utilizing a dichloromethane (DCM)/hexanes solvent system as the eluent. The purified product was subsequently recrystallized from a DCM/hexanes mixture to yield compound **1** as a blue-black solid.

|  | **1** Yield = 65.6%; ^1^H NMR (400 MHz, Chloroform-*d*) δ 7.69 (d, *J* = 16.3 Hz, 2H), 7.57 – 7.46 (m, 7H), 7.45 – 7.39 (m, 4H), 7.38 – 7.27 (m, 12H), 7.21 (s, 1H), 7.17 (s, 1H), 6.63 (s, 2H), 1.43 (s, 6H). FT-IR (KBr): 3428.14 cm^-1^, 3061.19 cm^-1^, 1492.88 cm^-1^, 1494.14 cm^-1^, 1164.19 cm^-1^, 991.15 cm^-1^, 731.45 cm^-1^, 691.08 cm^-1^, 537.28 cm^-1^. |
| --- | --- |

Synthesis of **PAGs-Bodipy**: This compound was synthesized according to a modified literature method^[2]^. A mixture of diphenyliodonium hexafluorophosphate (93.7 mg, 0.22 mmol), compound **1** (71.7 mg, 1 mmol) and copper acetate (1.8 mg, 0.01 mmol), dissolved in 3 mL chlorobenzene was heated to reflux at 125 ^o^C for 24 h. The reaction was monitored by thin-layer chromatography (TLC) and mass spectrometry. Upon completion, the solvent was evaporated under reduced pressure to give a dark-blue solid. The desired product was obtained as a dark-blue solid by silica column chromatography with dichloromethane and methanol as the eluent.

|  | **PAGs-Bodipy** Yield = 38.8%;^1^H NMR (400 MHz, Chloroform-*d*) δ 7.94 – 7.66 (m, 29H), 7.52 (s, 4H), 7.22 (s, 1H), 6.70 (s, 2H), 1.45 (s, 6H). FT-IR (KBr): 3359.98 cm^-1^, 2922.54 cm^-1^, 2852.01 cm^-1^, 1494.14 cm^-1^, 1166.27 cm^-1^, 994.48 cm^-1^, 733.74 cm^-1^, 684.21 cm^-1^, 532.05 cm^-1^. |
| --- | --- |

**3.2 Synthesis of Polyphenol-PEG**


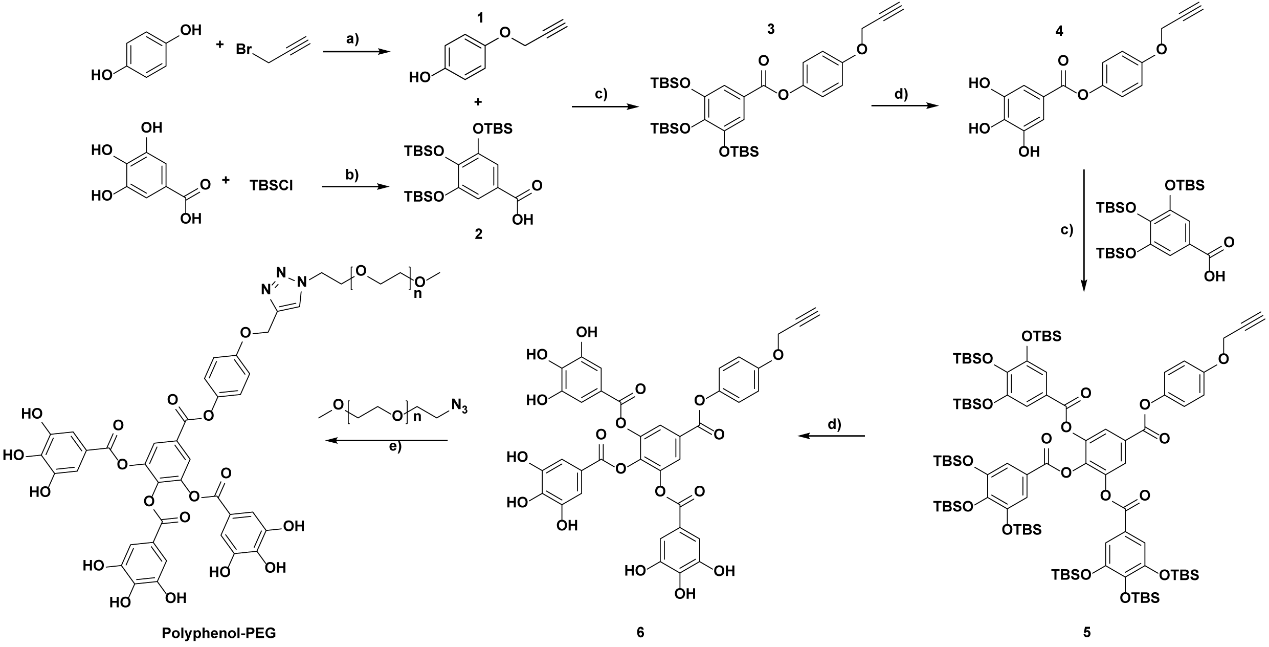


**Scheme S2.** Synthesis of Polyphenol-PEG. a) K_2_CO_3_, DMF, 60 ^o^C,4 h. b) DIEA, dry DMF, room temperature (r.t.), 2 h. c) DPTS, DIPC, CH_2_Cl_2_, r.t., 3 h. d) pyridine, THF, pyridine hydrofluoride, r.t., 2 h. e) PMDETA, CuBr, dry DMF, 60 ^o^C, 24 h.

Synthesis of **4-propargyloxyphenol** (**1**): This compound was synthesized according to a modified literature method^[3]^. To a stirred mixture of hydroquinone (0.220 g, 2.0 mmol) and potassium carbonate (0.138 g, 1.0 mmol) in dimethylformamide (5 mL) at 60 ^o^C was added propargyl bromide (0.117 g, 1.0 mmol). After four hours, dichloromethane (50 mL) was added, the organic layer was washed with 10 % HCl (30 mL), dried with magnesium sulfate, filtered and evaporated. Purification by column chromatography afforded yellow oil **1**.

|  | Yield: 50.3 %. ^1^H NMR (400 MHz, Chloroform-*d*): δ 7.94 – 7.66 (m, 29H), 7.52 (s, 4H), 7.22 (s, 1H), 6.70 (s, 2H), 1.45 (s, 6H). |
| --- | --- |

Synthesis of **3,4,5-tris((tert-butyldimethylsilyl)oxy)benzoic acid** (**2**): The detailed procedures are outlined in our previous literature^[4]^.

|  | Yield: 76 %. ^1^H NMR (500 MHz, ^1^H NMR (500 MHz, DMSO-*d*_6_): δ 7.12 (s, 2H), 0.96 (s, 9H), 0.89 (s, 18H), 0.21 (s, 12H), 0.11 (s, 6H). |
| --- | --- |

Synthesis of **4-(prop-2-yn-1-yloxy)phenyl 3,4,5-tris((tert-butyldimethylsilyl)oxy)- benzoate** (**3**): This compound was synthesized according to a modified literature method^[4]^. Compound **1** (1.59 g, 10.7 mmol), Compound **2** (6.00 g, 11.8 mmol) and DPTS (5.00 g, 17.1 mmol) were dissolved in CH_2_Cl_2_ and the mixture was allowed to stir for 5 min before DIPC (4.2 mL, 26.8 mmol) were added dropwise, with the reaction monitored by TLC. After 3 h, the reaction mixture was evaporated and the product was isolated by column chromatography, eluting with a mixture of petroleum ether and CH_2_Cl_2_ to give the product as a colorless solid.

|  | Yield: 41.1 %. ^1^H NMR (400 MHz, Chloroform-*d*): δ 9.40(s, 2H), 9.13(s, 1H), 7.14(d, J = 9.0 Hz, 2H), 7.09(s, 2H), 7.03(d, J = 9.0 Hz, 2H), 4.81(d, J = 2.3 Hz, 2H), 3.59(s, 1H). |
| --- | --- |

Synthesis of **4-(prop-2-yn-1-yloxy)phenyl 3,4,5-trihydroxybenzoate** (**4**): According to the procedure described in the literature^[4]^, to a solution of the tertbutyldimethylsilyl derivatives Compound **3** in a mixture of pyridine (0.96 mL mmol^−1^ of TBS groups) and THF (2 mL mmol^−1^ of TBS groups), HF·pyridine complex (0.96 mL mmol^-1^ of TBS group) was added and the reaction mixture was stirred at room temperature. After the complete disappearance of the starting material in thin layer chromatography (TLC), the reaction mixture was diluted with water (50 mL mmol^−1^) and the crude was extracted with ethyl acetate. The organic layer was washed with a saturated aqueous solution of CuSO_4_ and dried over MgSO_4_. The solvent was evaporated under reduced pressure and the resulting off-white solid was recrystallized from DCM/MeOH to afford the pure **4**.

|  | Yield: 61.2 %. ^1^H NMR (400 MHz, DMSO-*d*_6_): ^1^H NMR (400 MHz, DMSO-*d*_6_):δ 9.40(s, 2H), 9.13 (s, 1H), 7.14 (d, *J* = 9.0 Hz, 2H), 7.09 (s, 2H), 7.03 (d, *J* = 9.0 Hz, 2H), 4.81 (d, *J* = 2.3 Hz, 2H), 3.59 (s, 1H). |
| --- | --- |

Synthesis of **5-((4-(prop-2-yn-1-yloxy)phenoxy)carbonyl)benzene-1,2,3-triyl tris(3,4,5-tris((tert-butyldimethylsilyl)oxy)benzoate)** (**5**): Compound **2** (1.693 g, 3.3 mmol), Compound **4** (0.3003 g, 1 mmol) and DPTS (1.413 g, 4.8 mmol) were dissolved in CH_2_Cl_2_ and the mixture was allowed to stir for 5 min before of DIPC (1.2 mL, 7.5 mmol) were added dropwise, with the reaction monitored by TLC. After 3 h, the reaction mixture was evaporated and the product was isolated by column chromatography, eluting with a mixture of petroleum ether and CH_2_Cl_2_ to give the product as a colorless solid.

|  | Yield: 86.7%. ^1^H NMR (400 MHz, Chloroform-*d*): δ 8.08 (s, 2H), 7.31 (d, *J* = 2.6 Hz, 6H), 7.18 (d, *J* = 9.1 Hz, 2H), 7.06 (d, *J* = 9.1 Hz, 2H), 4.74 (d, *J* = 2.4 Hz, 2H), 2.58 (t, *J* = 2.4 Hz, 1H), 1.01 (d, *J* = 5.8 Hz, 27H), 0.95 (s, 36H), 0.90 (s, 18H), 0.22 (s, 24H), 0.14 (s, 24H), 0.11 (s, 6H). |
| --- | --- |

Synthesis of **5-((4-(prop-2-yn-1-yloxy)phenoxy)carbonyl)benzene-1,2,3-triyl tris(3,4,5-trihydroxybenzoate)** (**6**): To a solution of the tertbutyldimethylsilyl derivatives Compound **5** in a mixture of pyridine (0.96 mL mmol^−1^ of TBS groups) and THF (2 mL mmol^−1^ of TBS groups), HF·pyridine complex (0.96 mL mmol^-1^ of TBS group) was added and the reaction mixture was stirred at room temperature. After the complete disappearance of the starting material in thin layer chromatography (TLC), the reaction mixture was diluted with water (50 mL mmol^−1^) and the crude was extracted with ethyl acetate. The organic layer was washed with a saturated aqueous solution of CuSO_4_ and dried over MgSO_4_. The solvent was evaporated under reduced pressure and the resulting off-white solid was recrystallized from DCM/MeOH to afford the pure **6**.

|  | Yield: 78.7%. ^1^H NMR (400 MHz, DMSO-*d*_6_): δ 9.38 (s, 4H), 9.32 (s, 2H), 9.19 (d, *J* = 7.6 Hz, 3H), 8.06 (s, 2H), 7.29 (d, *J* = 9.0 Hz, 2H), 7.08 (d, *J* = 9.1 Hz, 2H), 7.00 (s, 4H), 6.90 (s, 2H), 4.83 (d, *J* = 2.3 Hz, 2H), 3.59 (t, *J* = 2.3 Hz, 1H). |
| --- | --- |

Synthesis of **Polyphenol-PEG**: According to the procedure described in the literature^[5, 6]^, about poly(ethylene glycol)-diazide (0.2000 g, 0.1 mmol, Mw = 2000), tetrakis(2-propynyloxymethyl)methane (0.0757 g, 0.1 mmol), PMDETA (0.020 mL, 0.1 mmol), and DMF (1 mL) were introduced into a small vial. After the mixture turned clear, the vial was degassed with argon for 20 min, and CuBr (0.0144 g, 0.1 mmol) was quickly added under ultrasonic agitation. The gelation point was reached in 1 min, and the reaction was allowed to continue for another 24 h at 60 ^o^C. A uniform hydrogel was obtained upon removal from the vial. The gel was transferred to EDTA (5%) solution to remove the copper ions and DMF. Finally, the gel was immersed into a large volume of pure deionized water to allow the water absorption.

|  | ^1^H NMR (400 MHz, Chloroform-*d*) δ 8.01 (s, 1H), 7.85 (s, 1H), 7.79 (s, 1H), 7.61 (s, 1H), 7.54 (s, 1H), 7.37 (s, 1H), 7.07 (s, 3H), 7.00 (s, 2H), 6.82 (d, *J* = 8.7 Hz, 1H), 6.77 (d, *J* = 9.0 Hz, 1H), 5.23 (s, 1H), 4.56 – 4.51 (m, 2H). |
| --- | --- |

**3.3 Synthesis of FeBPs assembly**

First, FeCl_3_·6H_2_O was mixed with an ethanol solution of PAGs-Bodipy in a 1:1 molar ratio. Subsequently, polyphenol-PEG ethanol solution was added dropwise to the above mixture and stirred overnight at room temperature. At the end of the reaction, the product was dissolved and dispersed in deionized water by evaporating the solvent. Finally, unreacted Fe^3+^ ions were removed by ultracentrifugation and the target products (FeBPs) were centrifuged using 10 kDa ultrafiltration centrifuge tubes at 5500 rpm for 30 min.

**4. Photophysical properties of PAGs-Bodipy**

**Photolysis of PAGs-Bodipy detected by HPLC:** PAGs-Bodipy solutions were prepared in CH_2_Cl_2_ and light (630 nm, 0.33 W/cm^2^) for 0, 5, 10, 30, 60, 90 and 120 min. The photolysis process was monitored using high performance liquid chromatography (HPLC). The mobile phase A was H_2_O and the mobile phase B was MeOH (v:v = 90:10). A gradient elution was performed using a mixture of CH_3_OH and H_2_O at a flow rate of 1 mL/min, and linear changes were observed according to the change of illumination time at 630 nm.

**Photoacid quantum yield:** Photoacid quantum yield was measured by using rhodamine B base as a sensor for photoacid^[2]^. The sample solutions were irradiated by an LED lamp with a wavelength of 630 nm, and the UV-Vis absorbance of rhodamine B at 555 nm was recorded every 5 minutes. The photoacid quantum yield of the tested sample is calculated according to the following equation:

$$\emptyset_{H^{+}}=\frac{{\Delta OD}_{555}\cdot N_{A}}{{10}^{3}\cdot\varepsilon_{555}^{RhB}\cdot I_{0}\cdot\left[ 1-{10}^{-\bar{D}} \right]\cdot\Delta t}$$

Where ${\Delta OD}_{555}$ is the change in the optical density measured at 555 nm during the production of rhodamine B, $N_{A}$ is the Avogadro number, $\varepsilon_{555}^{RhB}$ is the extinction coefficient of rhodamine B at 555 nm, $\bar{D}$ is the average absorbance, $I_{0}$ is the light intensity in photon/(cm^2^·s), Δt is the irradiation time in s.

**H^+^ production in PAGs-Bodipy**: Rhodamine B was employed as a fluorescent indicator for hydrogen ions (H^+^). The UV-Vis spectra of the interactions between rhodamine B and p-toluenesulfonic acid (TsOH) at different molar concentrations were systematically analyzed in acetonitrile solution. Through detailed spectral analysis, a quantitative relationship was established between the absorbance at 555 nm and the concentration of hydrogen ions (H^+^) in the solution, and a standard calibration curve was constructed accordingly. Subsequently, the concentration of H^+^ generated by PAGs-Bodipy after irradiation at 630 nm light was tested and calculated.

**5. H^+^ generation *in vitro***

Standard solutions with different pH values (from 5.4 to 8.8) were mixed with SNARF^TM^-1 in a volume ratio of 1:100 before measuring the pH change in solution. The fluorescence intensity of the different standard solutions at 580 nm and 640 nm was then determined by fluorescence spectroscopy. A calibration curve was established. The FeBPs solution was mixed 1:100 (v/v) with SNARF^TM^-1 before exposure to light at 630 nm. To study the pH change in the buffer environment, an aqueous solution of dispersed FeBPs was added to a PBS solution and mixed with SNARF^TM^-1. The mixed solution was then irradiated continuously for 10 minutes (630 nm, 0.33 W/cm^2^). The fluorescence intensity of the FeBPs solution was monitored by fluorescence spectroscopy at 580 nm and 640 nm every 1 minute at room temperature. The pH values for different exposure times were then calculated from the calibration curve.

**6. •OH production *in vitro***

The qualitative analysis of •OH production was performed by using the classic fluorescence method based on degradation of coumarin after trapping •OH selectivity. In brief, the fluorescence intensity at 452 nm of coumarin solution under 630 light irradiation (0.33 W/cm^2^) were measured after centrifugal and removal of the potential interference of FeBPs on the fluorescence signal.

Another qualitative analysis of •OH production was performed by using electron spin resonance spectroscopy to further conform the •OH generation with 5-dimethyl-1-pyrroline N-oxide (DMPO) as a spin trap. The subsequent reaction groups respectively contained the following additions: H_2_O_2_, FeBPs, FeBPs + H_2_O_2_ without 630 light, FeBPs + H_2_O_2_ with 630 light, FeP, FeP + H_2_O_2_ without 630 nm light. The concentration of H_2_O_2_ is 100 μM.

**7. Oxidation reduction potential**

Cyclic voltammetry was conducted using a solution containing 0.1 mM FeBPs and 1 M KCl as the supporting electrolyte. The standard three-electrode cell consisted of a glassy carbon working electrode, an Ag/AgCl reference electrode, and a platinum wire counter electrode. Before each measurement, the solution was purged with N_2_ to remove dissolved oxygen. The standard used was 0.1 mM potassium ferrocyanide and 0.1 mM potassium ferricyanide. The current was measured from -1.4 V to 0 V at a scan rate of 50 mV/s under continuous illumination at 630 nm light (0.33 W/cm^2^).

**8. Valence changes of Fe**

The concentration of Fe^2+^ and Fe^3+^ ions in the FeBPs during 630 nm light irradiation (0.33 W/cm^2^) was determined by a standard iron detection method. Fe^2+^ can form stable complexes with 1,10-phenanthroline with a maximum absorbance of 510 nm. The absorbance of the complexes was monitored using a UV-Vis spectrophotometer.

**9. XAFS analysis**

XAFS data handling, including reduction, analysis, and EXAFS fitting, was carried out with the Athena and Artemis modules from the Demeter software package^[7]^, which integrates the FEFF6 program^[8]^ for EXAFS spectral fitting. Sample energy scales were calibrated using a standard Fe foil, measured in parallel as a reference. Pre-edge treatment involved subtracting a linear baseline, followed by edge-jump normalization *via* Athena. The χ(k) oscillatory component was isolated by removing a smooth third-order polynomial that approximated the atomic absorption background. Fourier transformation of the k^2^-weighted χ(k) data was performed with a Hanning window (Δk = 1.0). For EXAFS structural refinement, nonlinear least-squares fitting in R-space was executed in Artemis to obtain global parameters: coordination number (CN), bond length (R), Debye-Waller factor (σ^2^), and energy offset (ΔE_0_). The amplitude reduction factor (S_0_^2^) required for coordination number determination was derived from fitting the Fe foil EXAFS and fixed throughout sample analysis.

Wavelet transform analysis was implemented in the WTEXAFS code using χ(k) data exported from Athena. The analysis parameters were set as follows: R range 0–4 Å, k range 0–12 Å^-1^, k weight = 2, and a Morlet wavelet (κ = 10, σ = 1) as the mother function to visualize the spatial distribution of scattering paths.

**10. DFT calculation**

To thoroughly investigate the acid-induced coordination transition mechanism of FeBPs and its promotion of Fe^2+^ regeneration and ROS generation, we performed density functional theory (DFT) calculations based on the previously reported polyphenol complex structures^[4]^. We simulated the structures of iron-polyphenol complexes with different coordination states and calculated the activation energies of H_2_O_2_ under these different coordination states. All theoretical calculations were performed using density functional theory (DFT) as implemented in the Vienna Ab initio Simulation Package (VASP)^[9]^. The frozen-core all-electron projector-augmented-wave (PAW) method was employed to accurately describe the ion-electron interactions^[10]^. The Perdew-Burke-Ernzerhof (PBE) generalized gradient approximation (GGA) was used for the exchange-correlation functional^[10, 11]^. The plane wave basis was truncated at an energy cutoff of 400 eV, and the geometry relaxation was conducted with a force convergence criterion of less than 0.02 eV/Å for each atom. The Brillouin zone integration was carried out using a 3×3×3 k-point sampling scheme. Self-consistent field calculations were performed with a convergence energy threshold of 10^-5^ eV. To account for van der Waals interactions, the DFT-D3 method was incorporated^[12]^.

**11. MRI performance** ***in vitro***

MRI of the FeBPs and FePs solutions with various Fe concentrations was performed using a small animal MRI (1.0 T) scanner with associated software (NMG1-Analyst, Niumag) for three replicates. The longitudinal (*T*_1_) and transverse (*T*_2_) relaxation times and the corresponding MR images were measured on a 1.0 T MRI system (NMG1-Analyst, Niumag).

**12. Cell culture and cell viability assay**

The B16-F10, A549 and HUVEC cell lines were cultivated in DMEM medium (BasaMedia), with the addition of 10% foetal bovine serum (FBS) and 1 % penicillin/streptomycin (PS). The maintenance of the cells was conducted at a temperature of 37 ^o^C within an atmosphere containing 5 % CO_2_ and 95 % relative humidity.

An assessment of the in vitro cellular toxicity of FeBPs was conducted by means of a Cell Counting Kit-8 (CCK-8, Beyotime) assay with different cells. In summary, cells were seeded in 96-well cell culture plates at a density of 15,000 cells per well for a 24-hour period to allow attachment. Subsequently, a series of concentrations of FeBPs dispersed in DMEM (0, 0.0375, 0.0750, 0.150, 0.300, and 0.600 mM) were added separately. Following a 24-hour dark incubation period, the medium was removed. The cells were then rinsed twice with Phosphate Buffered Saline (PBS), after which 10 µL of CCK-8 was added and the cells were incubated at 37 ^o^C for 30 minutes. Cell viability was calculated by measuring the optical density at a wavelength of 490 nm, using a microplate reader (Varioskan Flash, Thermo Fisher Scientific).

**13. Cellular uptake and colocalization**

**Cellular uptake:** B16-F10 and A549 cells were co-incubated with FeBPs. First, B16-F10 and A549 cells were seeded into glass-bottom culture dishes containing 1 mL of culture medium at a density of 1 × 10^5^ cells per dish. After a 24-hour adhesion period, FeBPs were added to 1 mL of cell culture medium to achieve a final concentration of 0.2 mM, and this medium mixture was added to the culture dishes. Following different incubation times (0, 2, 4, and 8 hours), the cells were washed with cold phosphate-buffered saline (PBS) and then fixed with 4 % paraformaldehyde for 20 minutes. Fluorescence images were obtained using confocal laser scanning microscopy (CLSM, TCS SP5, Leica). The excitation wavelength of FeBPs is 633 nm.

**Colocalization:** B16-F10 or A549 cells cultured in glass-bottom dishes containing 1 mL of culture medium were incubated with 0.2 mM FeBPs at 37 ^o^C for 4 h. The cells were then co-incubated with Mito-Tracker Green (MTG, a mitochondria-specific green fluorescent probe) for an additional 0.5 h. After washing three times with PBS, the cells were immediately imaged using a laser confocal microscope. The excitation wavelengths for FeBPs and MTG were 633 nm and 488 nm, respectively. Fluorescence images were obtained using CLSM with associated software (LAS X, Leica Microsystems CMS GmbH, version 4.6.0.27096)

**14. 630 nm light-triggered intracellular acidification and ROS generation**

B16-F10 and A549 cells were cultured as described above and treated with various formulations (Control, H_2_O_2_, Light, FeBPs, FeBPs + H_2_O_2_, FeBPs + Light, FeBPs + H_2_O_2_ + Light, equal concentration of FeBPs (0.2 mM) and H_2_O_2_ (100 μM)) for 4 hours. ROS were reacted with DCFH-DA for 30 min at 37 ^o^C before irradiation at 630 nm (0.33 W/cm^2^, 10 min). The resulting cellular ROS oxidizes DCF, serving as an indicator for ROS production, with excitation at 488 nm. Intracellular ROS levels were evaluated using confocal laser scanning microscopy (CLSM) and flow cytometry (Agilent NovoCyte 2000, USA) after irradiation without subsequent incubation. At the same time, SNARF^TM^-1 was used as a pH indicator, excited at 488 nm. Fluorescent images were captured using CLSM, and the fluorescence ratio of intensities at 580 nm and 640 nm was calculated using a calibration curve.

**15. Mitochondrial membrane potential**

The B16-F10 cells or A549 cells were cultivated as outlined above. Subsequently, the wells were treated with various formulations (PBS, H_2_O_2_, Light, FeBPs, FeBPs + H_2_O_2_, FeBPs + Light, FeBPs + H_2_O_2_ + Light, equal concentration of FeBPs 0.2 mM) and then incubated for 4 h. The concentration of H_2_O_2_ is 100 μM. Following 630 nm light irradiation (0.33 W/cm^2^, 10 min), the probe (JC-1) for measuring mitochondrial membrane potential was incubated with cells for 10 min at 37 ^o^C. Subsequently, the cells were subjected to three washes with cold phosphate-buffered saline (PBS) prior to cell collection. The mitochondrial membrane potential was determined on CLSM. The excitation wavelengths of the JC-1 monomer and the JC-1 aggregate are 488 nm and 535 nm, respectively. The data were obtained from 15,000 cells for each sample.

**16. Cell apoptosis**

The B16-F10 cells or A549 cells were cultured as described above, treated with different formulations (PBS, H_2_O_2_, Light, FeBPs, FeBPs + H_2_O_2_, FeBPs + Light, FeBPs + H_2_O_2_ + Light, equal concentration of FeBPs 0.2 mM) and then incubated for 4 h. The concentration of H_2_O_2_ is 100 μM. The cells were stained by Calcein-AM/PI staining working solution for 30 min at 37 ^o^C. Viable cells can be stained by Calcein AM (excitation wavelength = 488 nm), while dead cells can be stained by PI (excitation wavelength = 535 nm). Additionally, cellular apoptosis was also assessed with an Annexin V-FITC Apoptosis Detection Kit.

**17. Biosafety of FeBPs *in vivo***

All animal experiments were performed in strict accordance with the requirements of Shanghai Normal University (Shanghai, China). Female BALB/c mice and nude mice (five weeks old) were obtained from Shanghai Legen Biotechnology Co., Ltd. Normal mice were randomly divided into two groups (n = 3): control group and FeBPs group. All biocompatibility assessments were performed after a single intravenous injection (0.2 mg [Fe] kg^-1^ in 200 µL saline) via the tail vein, with blood and tissue samples collected 24 h post-injection for routine blood analyses, biochemical analyses, and H&E section staining.

**18. Acidification in tumors**

To establish a calibration for pH measurement in tumor sites, standard solutions with pH values of 5.0, 5.5, 6.0, 6.5, 7.0, and 7.5 were mixed with SNARF^TM^-1 at a volume ratio of 1:100. The fluorescence intensities at 580 nm and 640 nm for these solutions were measured using a small animal live imaging optical 3D imaging system with associated software (PerkinElmer, USA), yielding a calibration curve.

Given that the melanin in the B16-F10 mouse model absorbs light and interferes with fluorescence imaging, we selected the more commonly used 4T1 mouse model as a fluorescence imaging model to monitor the acidity changes in the tumor region under 630 nm light irradiation (0.33 W/cm^2^, 10 min). Mice subjected to various treatments (Control, Light, FeBPs, FeBPs + Light, with FeBPs at 0.2 mM) were intratumorally injected with the pH-sensitive fluorescent dye SNARF^TM^-1 30 min before imaging. Mice in the light-triggering groups were then irradiated with 630 nm light. The pH values in the tumor sites were subsequently determined based on the ratio of fluorescence intensities at 580 nm and 640 nm.

**19. ROS generation in tumors**

In order to investigate the generation of ROS in situ, DCFH-DA was intratumorally administered before imaging by an animal imaging system (PerkinElmer, USA). After this, the 4T1 mice were anaesthetized and the tumor sites were irradiated by 630 nm light (0.33 W/cm^2^, 10 min). Subsequently, the green ROS was detected and measured by means of an animal imaging system. This was followed by further quantitative analysis for different treatments (Control, Light, FeBPs, FeBPs + Light, with FeBPs at 0.2 mM).

**20. *T*_1_ MRI *in vivo***

Tumors models were established via subcutaneous injections of B16-F10 cells into the of the mice. To investigate the MRI contrast of FeBPs in tumors, BALB/c mice were anesthetized with 100 mL of chloral hydrate (10 wt%). Prior to MRI, the mice were intravenously injected with FeBPs at a dose of 0.2 mg [Fe] kg^-1^. MRI was then performed at predetermined time points post-injection. *T*_1_-weighted magnetic resonance images were acquired using a 1.0 T MRI scanner with the following parameters: repetition time, 500 ms; echo time, 20 ms; field of view, 80 × 80 mm^2^; matrix size, 256 × 192; and slice width, 2.0 mm. The relative MR signal value (Signal-to-Noise Ratio, SNR) was calculated according to the formula:

$$SNR={SI}_{mean}/{SD}_{noise}$$

$$Relative MR signal value={SNR}_{post}/{SNR}_{pre}\times100\%$$

**21. Inhibition of tumor growth**

B16-F10 tumor cells were established in 6-week-old female BALB/c mice (≈ 20 g) by subcutaneously injecting B16-F10 cells suspended in 100 μL PBS into the armpit. The tumor-bearing mice were then randomly divided into four groups (n = 5) for different treatments (Control, Light, FeBPs, FeBPs + Light). When the tumor volume reached approximately 80 mm^3^, mice were intratumorally injected with 100 μL PBS or FeBPs (0.2 mg/kg). At 20 min post-injection, the light-treated groups were exposed to 630 nm light for 10 min at 0.33 W/cm^2^. After treatment, one mouse from each group was sacrificed for H&E, Ki67, and TUNEL staining of paraffin-embedded and sectioned tumor tissues. The body weights and tumor sizes of the mice were recorded every two days from day 0 to day 21 in the remaining groups.

**22. Data analysis**

Magnetic resonance (MR) signal intensities were quantified through signal-to-noise ratio (SNR) analysis using the MRI scanner and its associated software. The positioning of tumors or liver tissues was guided by the MR images. Ratiometric MR images of the tumor or liver were generated using the Calculator Plus Plugin available in ImageJ software. Data are presented as mean ± standard deviation (s.d.), unless otherwise specified.

Fluorescence (FL) intensity was measured using region of interest (ROI) analysis on a small-animal in vivo imaging optical 3D imaging system (PerkinElmer, USA) equipped with relevant software. The position of the tumor in the fluorescence image was determined based on the fluorescence signal, and the tumor region was defined as the ROI. The ROIs in the acquired 580 nm and 640 nm FL images were automatically outlined at the tumor position, guided by the corresponding fluorescence image. To calculate the pH change, the FL_580_/FL_640_ ratio was first determined by dividing the fluorescence intensity at 580 nm by the fluorescence intensity at 640 nm for each FL image. The corresponding ratio for each FL image was then substituted into the standard calibration curve to derive the pH value of the tumor region.

**23. Statistical analysis**

When the two groups were compared, Student’s *t*-test was performed, whereas multiple comparisons were conducted using two-way analysis of variance (ANOVA), with analyses performed in GraphPad Prism 6 (GraphPad Software Inc., CA, USA).

**24. Supplementary Tables and Figures**

**Figure S1.** ^1^H NMR spectra of compound 1 in CDCl_3_.

**Figure S2.** ^1^H NMR spectra of PAGs-Bodipy in CDCl_3_.


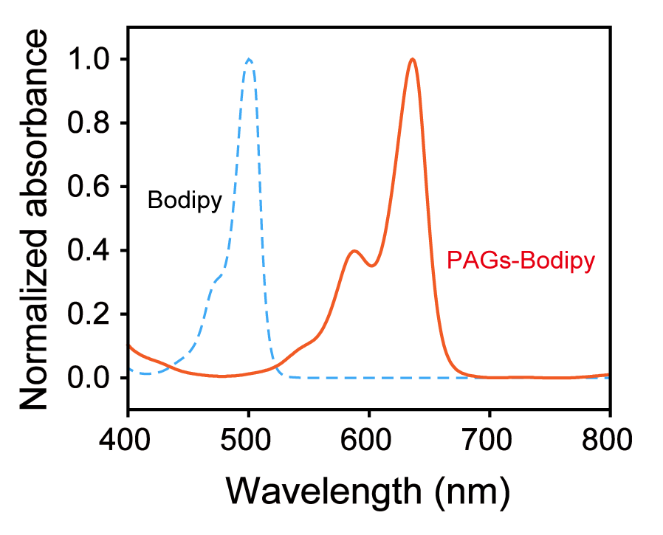


**Figure S3.** UV-vis absorption spectra of Bodipy (blue) and PAGs-Bodipy (red) in in CH_2_Cl_2_.


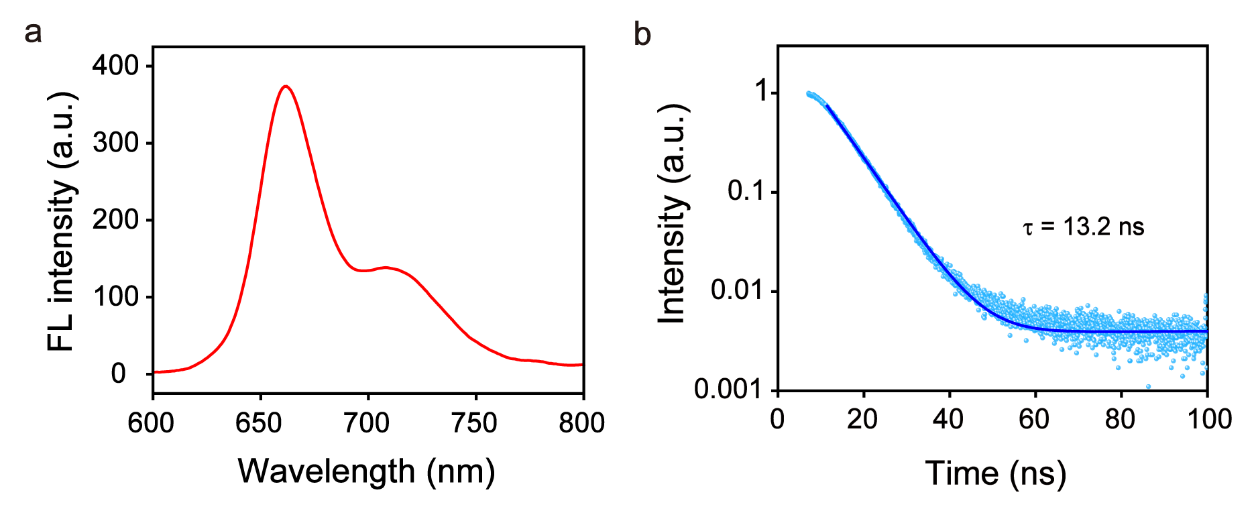


**Figure S4.** (a) Fluorescence emission spectrum of PAGs-Bodipy (λ_ex_ = 630 nm). (b) Fluorescence lifetime of PAGs-Bodipy in CH_2_Cl_2_.

**Table S1.** The photophysical data of PAGs-Bodipy.

|  | $\lambda_{max}^{abs}$ (nm) ^a^ | $\lambda_{max}^{em}$ (nm) ^b^ | τ (ns) ^c^ |
| --- | --- | --- | --- |
| **PAGs-Bodipy** | 636 | 662 | 13.2 |

^a^ Wavelength of maximum absorption.

^b^ Wavelength of maximum emission.

^c^ Fluorescence lifetime.

Photoluminescence (PL) spectra were performed on FLS980 fluorescence spectrophotometer (Edinburgh Instruments).

Fitted Time-Resolved PL Results. The average lifetime (τ_A_) of excited electrons in **PAGs-Bodipy** in CH_2_Cl_2_ can be calculated by the following equation S:

$$\bar{\tau}=\frac{\frac{B_{1}}{B_{1}+B_{2}}\tau_{1}^{2}+\frac{B_{2}}{B_{1}+B_{2}}\tau_{2}^{2}}{\frac{B_{1}}{B_{1}+B_{2}}\tau_{1}+\frac{B_{2}}{B_{1}+B_{2}}\tau_{2}}=13.2 ns$$

$$\tau_{1}=4.0119 ns$$

$$\tau_{2}=18.2716 ns$$

$$B_{1}=257.742$$

$$B_{2}=102.704$$

$$\chi^{2}=1.270$$

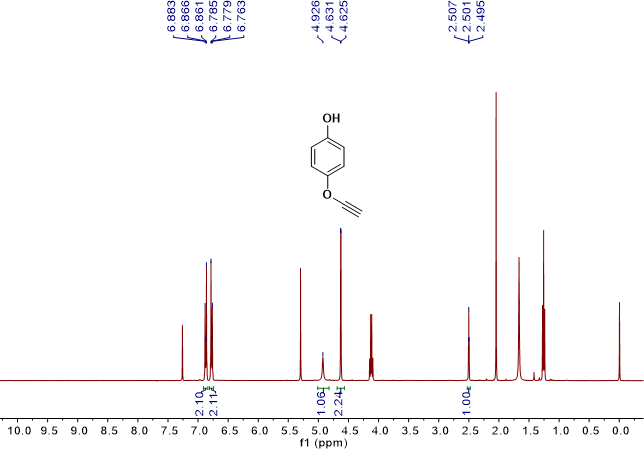


**Figure S5.** ^1^H NMR spectra of 4-(ethynyloxy)phenol (1) in CDCl_3_.

_
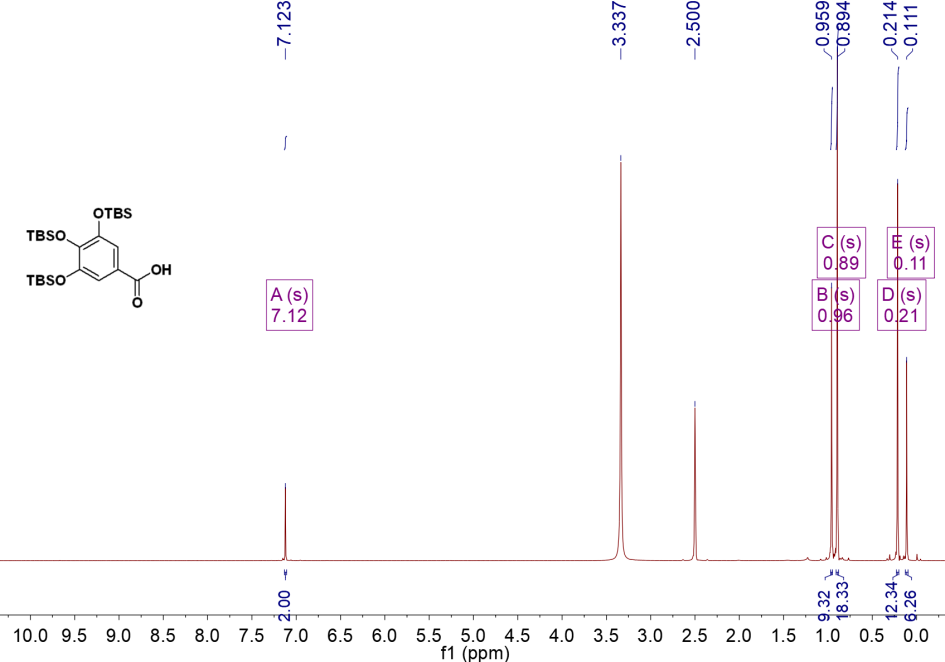
_

**Figure S6.** ^1^H NMR spectra of 3,4,5-tris((tert-butyldimethylsilyl)oxy)benzoic acid (2) in DMSO-*d*_6_.


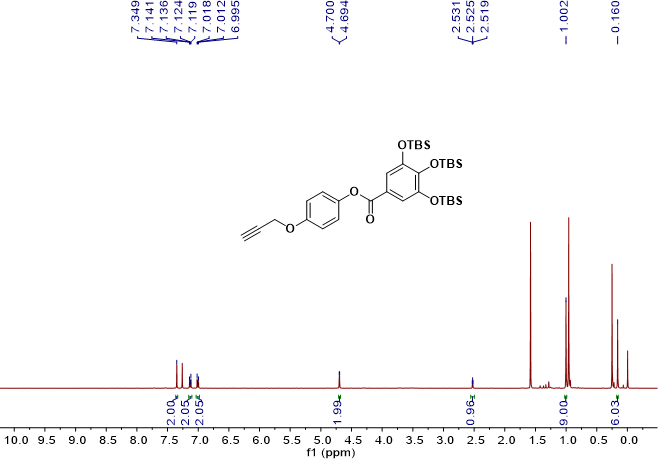


**Figure S7.** ^1^H NMR spectra of 4-(prop-2-yn-1-yloxy)phenyl 3,4,5-tris((tert- butyldimethylsilyl)oxy)benzoate (3) in CDCl_3_.


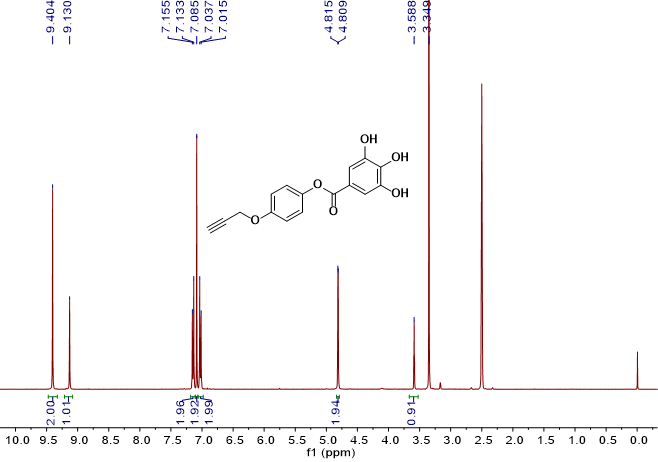


**Figure S8.** ^1^H NMR spectra of 4-(prop-2-yn-1-yloxy)phenyl 3,4,5-trihydroxybenzoate (4) in DMSO-*d*_6_.


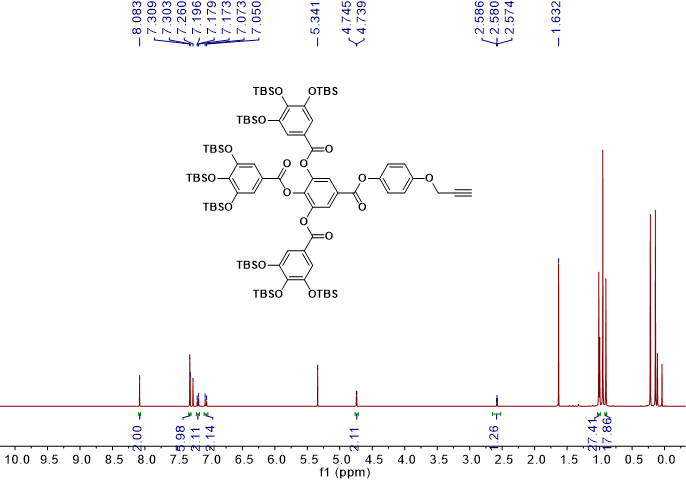


**Figure S9.** ^1^H NMR spectra of 5-((4-(prop-2-yn-1-yloxy)phenoxy)carbonyl) benzene-1,2,3-triyl tris(3,4,5-tris((tert-butyldimethylsilyl)oxy)benzoate) (5) in CDCl_3_.


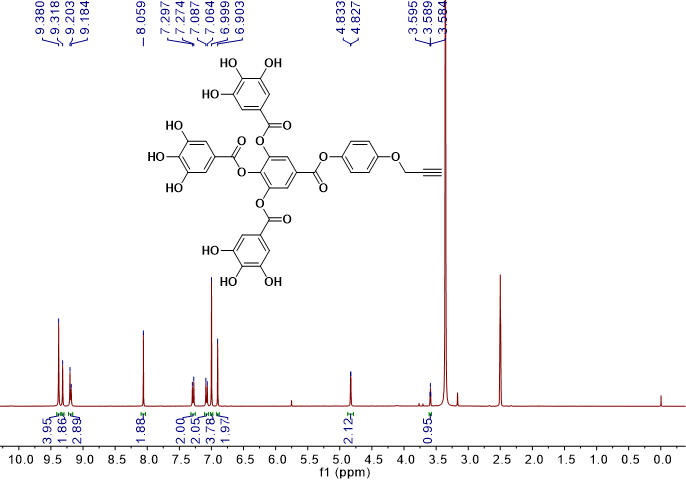


**Figure S10.** ^1^H NMR spectra of 5-((4-(prop-2-yn-1-yloxy)phenoxy)carbonyl) benzene-1,2,3-triyl tris(3,4,5-trihydroxybenzoate) (6) in DMSO-*d*_6_.


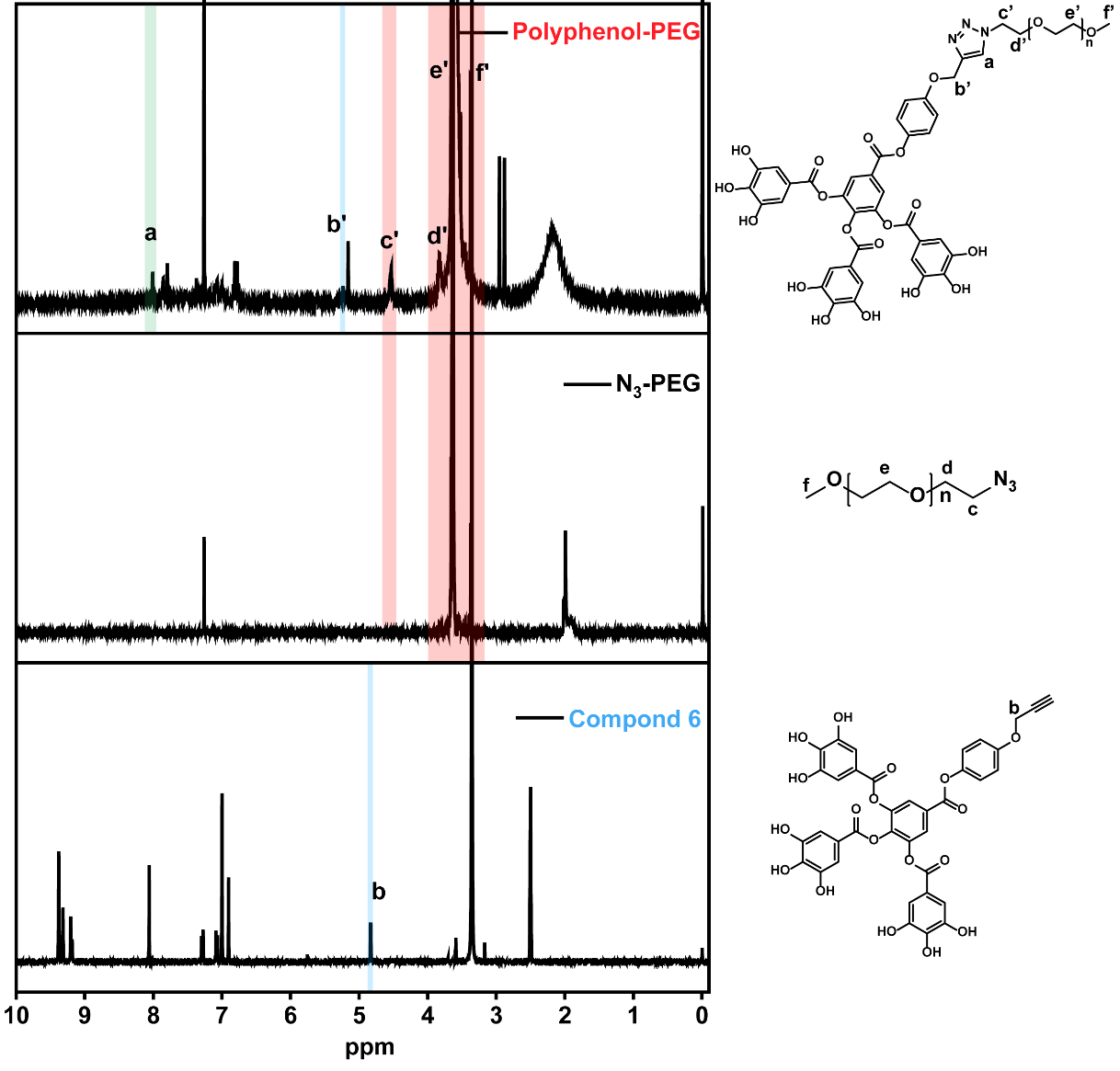


**Figure S11.** ^1^H NMR spectra of Polyphenol-PEG.


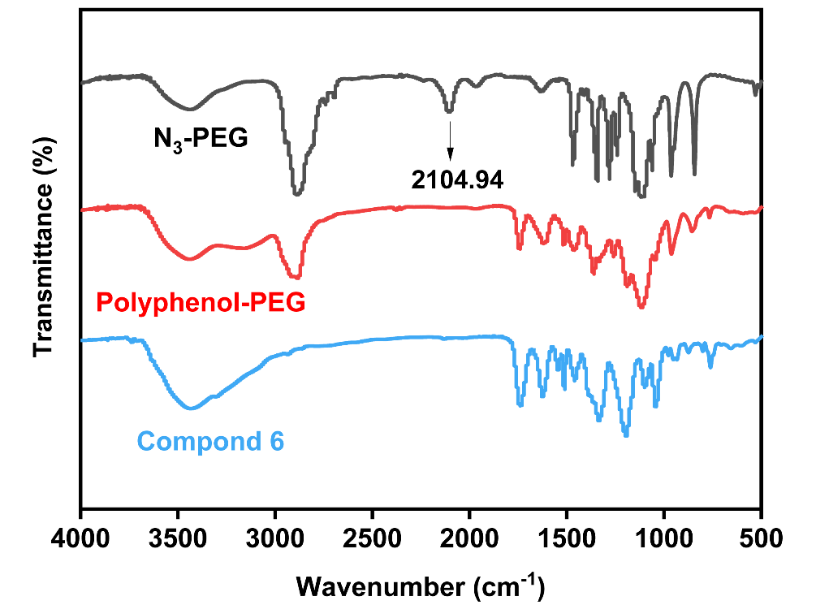


**Figure S12.** FT-IR spectra of Polyphenol-PEG.

**Table S2.** EXAFS fitting parameters at the Fe *K*-edge for various samples. (*Ѕ*_0_^2^=0.77)

| Sample | Shell | CN^a^ | R(Å)^b^ | σ^2^(Å^2^)^c^ | ΔE_0_(eV)^d^ | K-range/Å^-1^ | R-range/Å | R factor |
| --- | --- | --- | --- | --- | --- | --- | --- | --- |
| Fe foil | Fe-Fe | 8* | 2.47±0.01 | 0.0051 | 6.6±0.9 | 3.0-14.2 | 1.0-3.0 | 0.0029 |
|  | Fe-Fe | 6* | 2.85±0.01 | 0.0066 |  |  |  |  |
| FeBPs | Fe-O | 4.4±0.3 | 2.04±0.01 | 0.0118 | 1.2±1.7 | 3.0-10.1 | 1.0-2.5 | 0.0134 |
| FeO | Fe-O | 3.8±0.3 | 2.11±0.01 | 0.0100 | -2.8±0.7 | 3.0-11.1 | 1.0-3.0 | 0.0063 |
|  | Fe-Fe | 12.0±0.5 | 3.07±0.01 | 0.0142 |  |  |  |  |
| Fe_2_O_3_ | Fe-O | 6.3±0.4 | 2.11±0.01 | 0.0102 | -8.8±1.5 | 3.0-11.1 | 1.0-4.0 | 0.0073 |
|  | Fe-Fe | 3.0±0.3 | 3.07±0.01 | 0.0055 |  |  |  |  |
|  | Fe-Fe1 | 8.3±1.1 | 3.07±0.01 | 0.0093 |  |  |  |  |
|  | Fe-Fe2 | 8.0±1.1 | 3.07±0.01 | 0.0098 |  |  |  |  |

*^a^CN*, coordination number; *^b^R*, the distance to the neighboring atom; *^c^σ*^2^, Debye-Waller factor , the Mean Square Relative Displacement (MSRD); *^d^ΔE*_0_, inner potential correction; *R* factor indicates the goodness of the fit. *S*0^2^ was fixed according to the experimental EXAFS fit of Fe foil by fixing *CN* as the known crystallographic value. * This value was fixed during EXAFS fitting, based on the known structure of Fe. Error bounds that characterize the structural parameters obtained by EXAFS spectroscopy were estimated as CN ± 20%; R ± 1%; σ2 ± 20%; ΔE0 ± 20%. A reasonable range of EXAFS fitting parameters: 0.600 < *Ѕ*_0_^2^ < 1.000; *CN >* 0; *σ*^2^ > 0 Å^2^; |Δ*E*_0_| < 15 eV; *R* factor < 0.02, the Debye-Waller factor (σ^2^) was set.


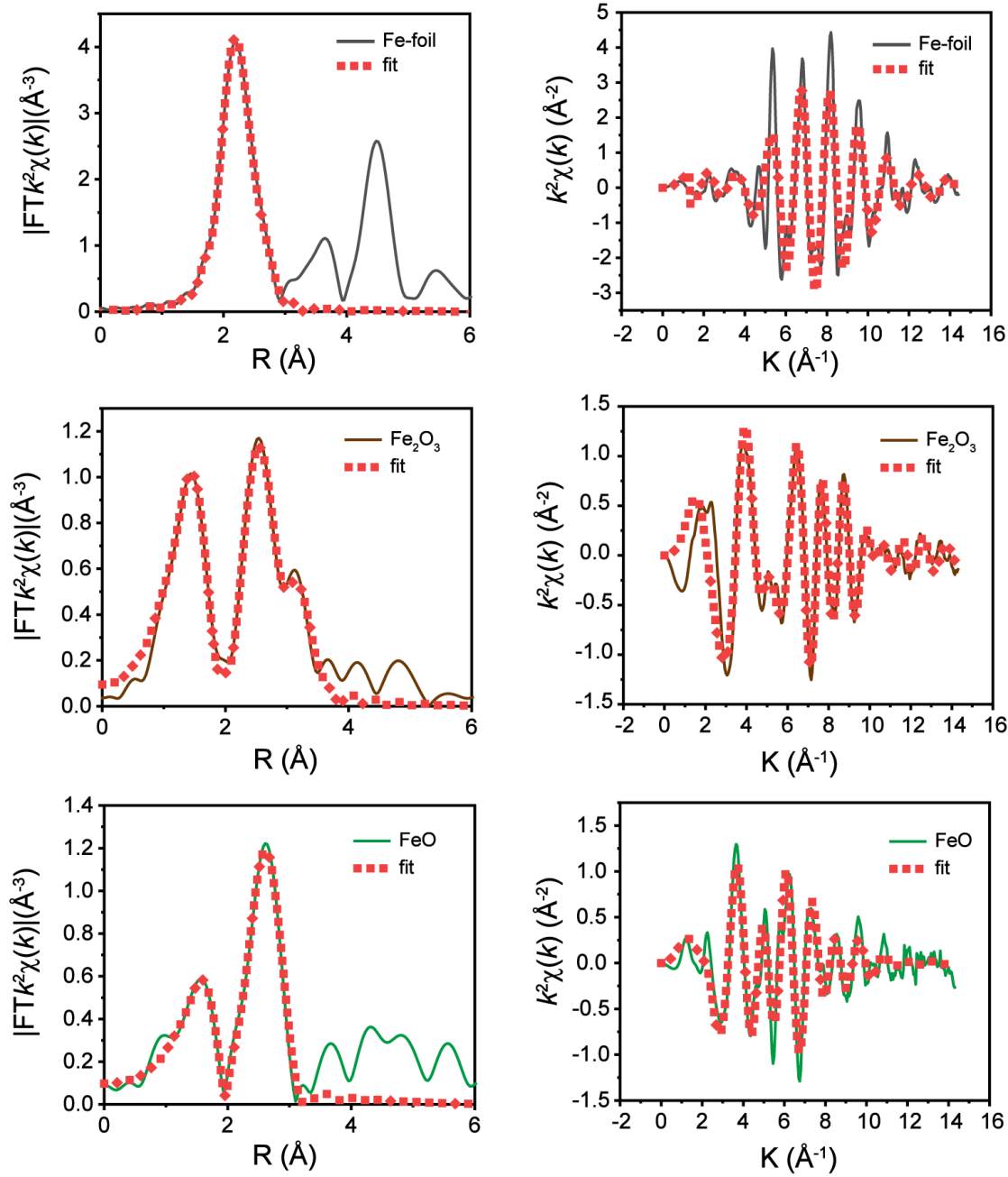


**Figure S13.** Nonlinear fitting of the EXAFS spectra for Fe foil, Fe_2_O_3_ and FeO showing R - space radial distributions (left column) and their k - space oscillations (right column).


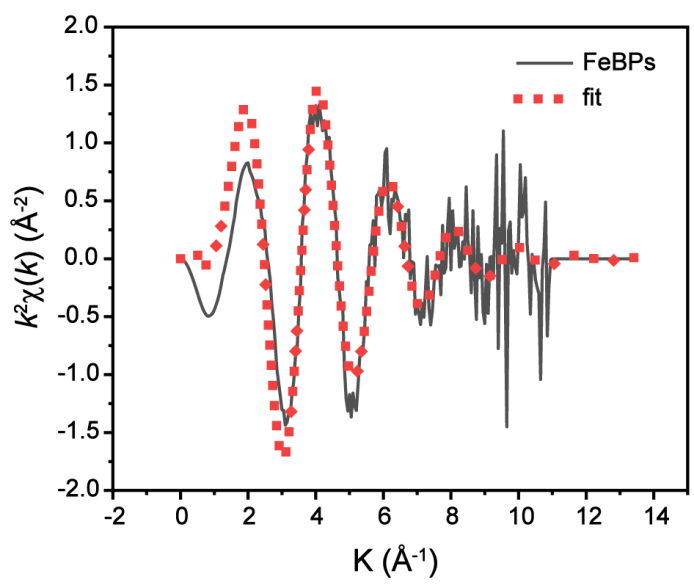


**Figure S14.** k - space EXAFS spectrum of the FeBPs.


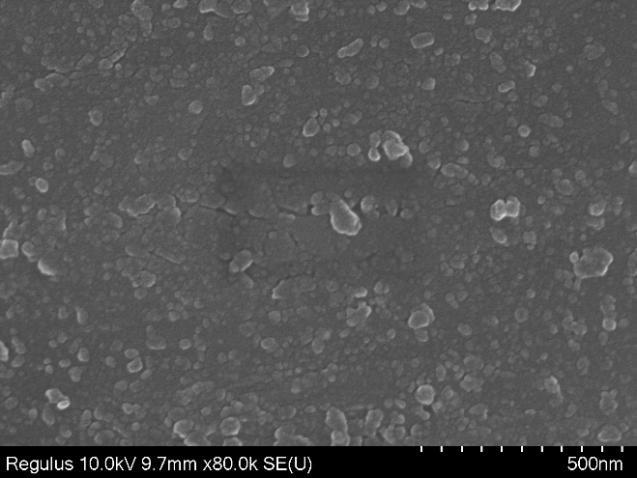


**Figure S15.** SEM of FeBPs. Scare bar = 500 nm.


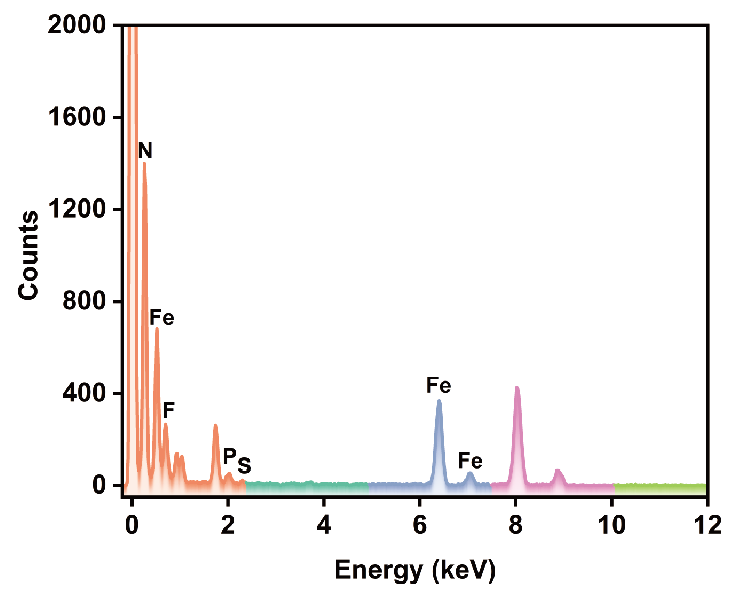


**Figure S16.** Energy dispersive X-ray spectroscopy of FeBPs.

**Table S3**. Elemental content analysis of Fe and B measured in FeBPs by ICP-MS.

| **Elements** | **Element concentration (μg/L) in digestion solution/original sample solution** |
| --- | --- |
| Fe | 38297 |
|  | 36319 |
|  | 34894 |
| B | 1170 |
|  | 1122 |
|  | 1204 |


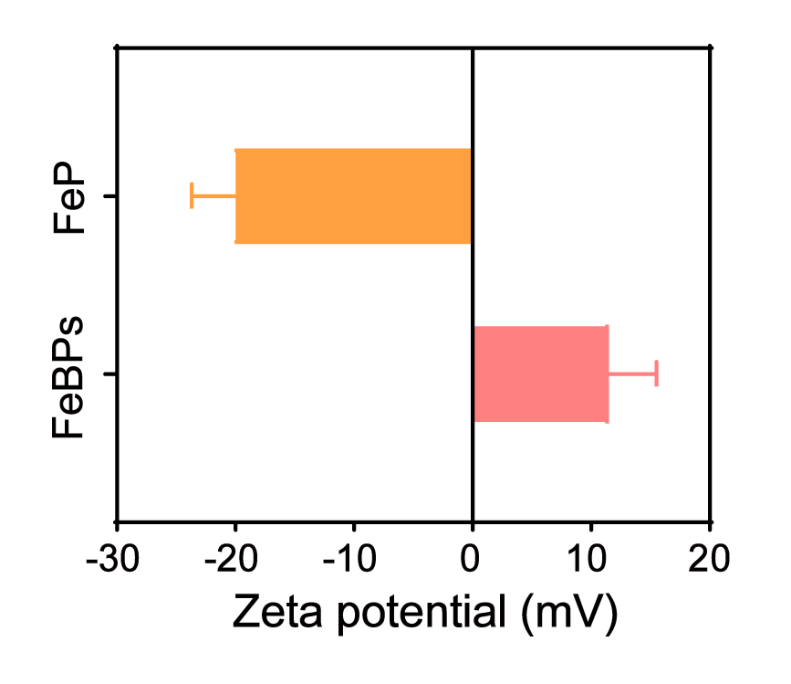


**Figure S17.** Zeta potential of FeBPs and FeP without Bodipy (n = 3).


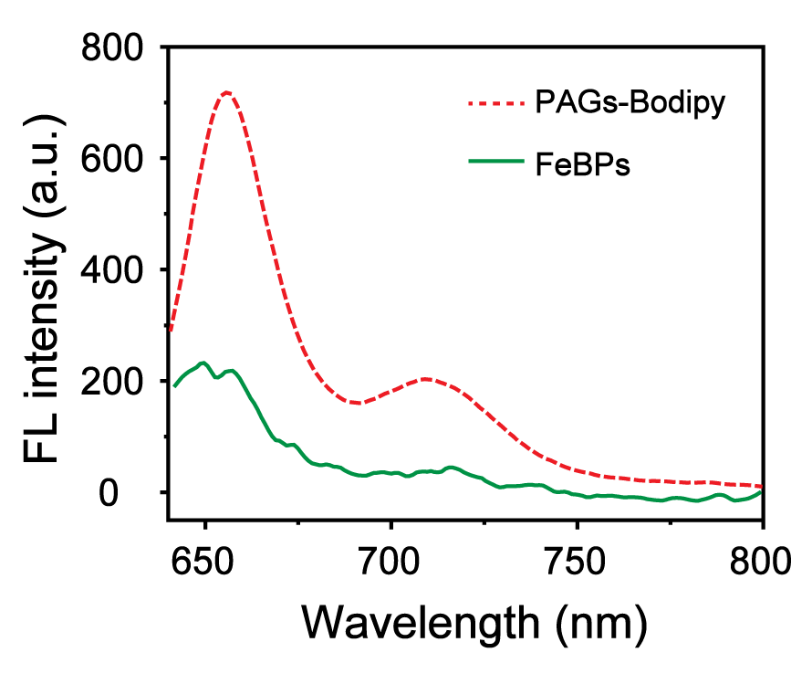


**Figure S18.** Fluorescence emission spectra of FeBPs and PAGs-Bodipy (λ_ex_ = 630 nm).

**
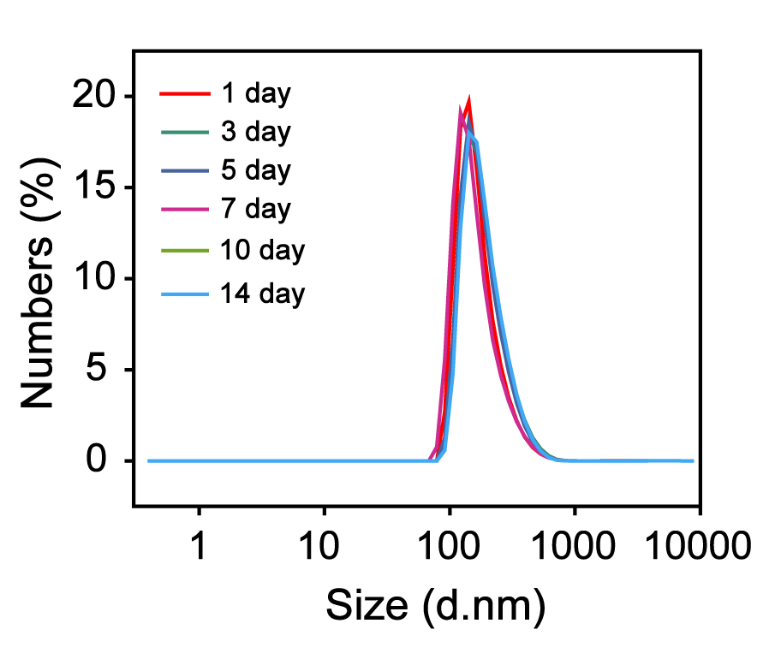
**

**Figure S19.** Hydrodynamic diameter of FeBPs in H_2_O monitored over 14 days.

**
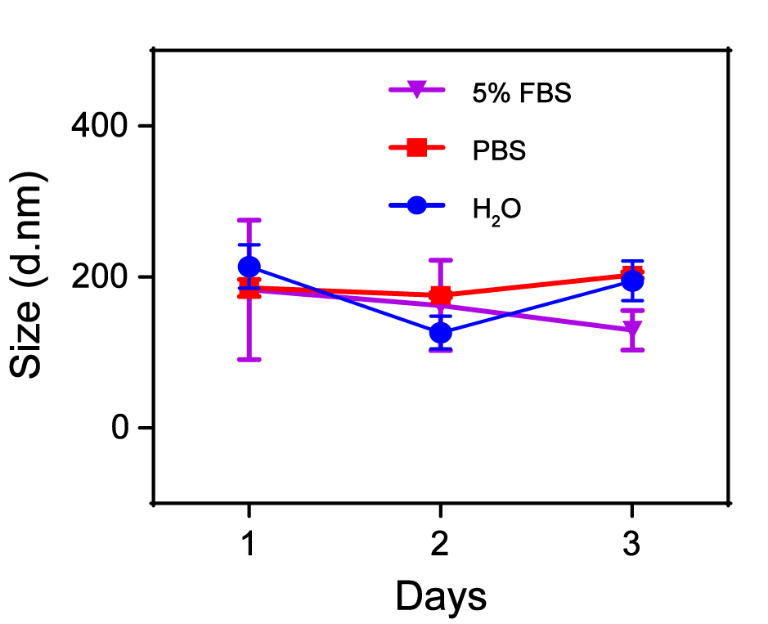
**

**Figure S20.** Size distribution of FeBPs in H_2_O, phosphate buffer solution (PBS) and 5% fetal bovine serum (FBS) over 72 h.


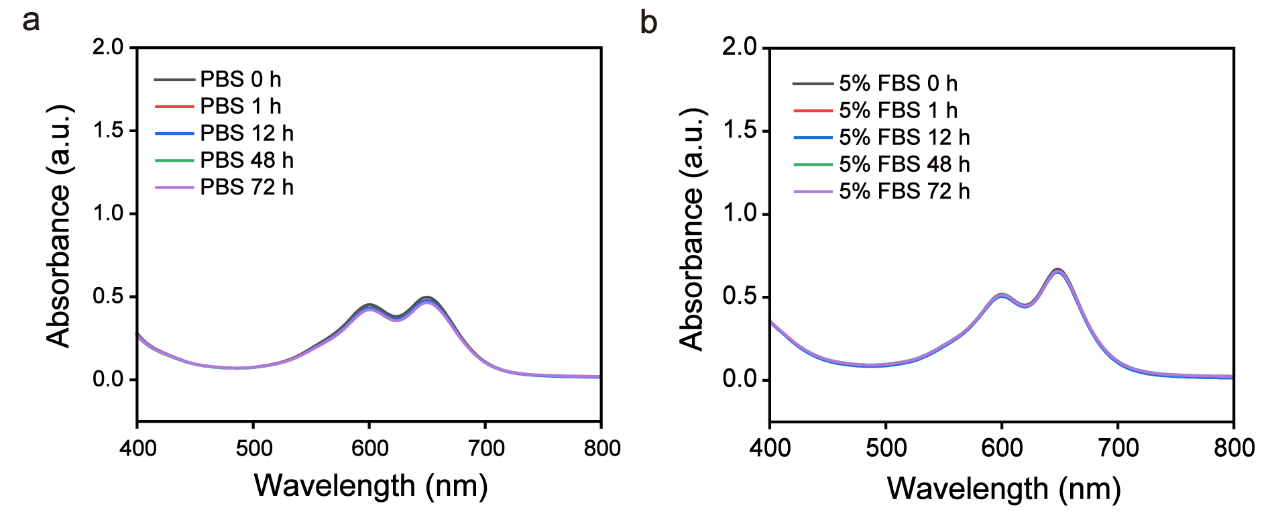


**Figure S21.** UV-vis spectra of FeBPs in (a) PBS and (b) 5% FBS over 72 h.

**
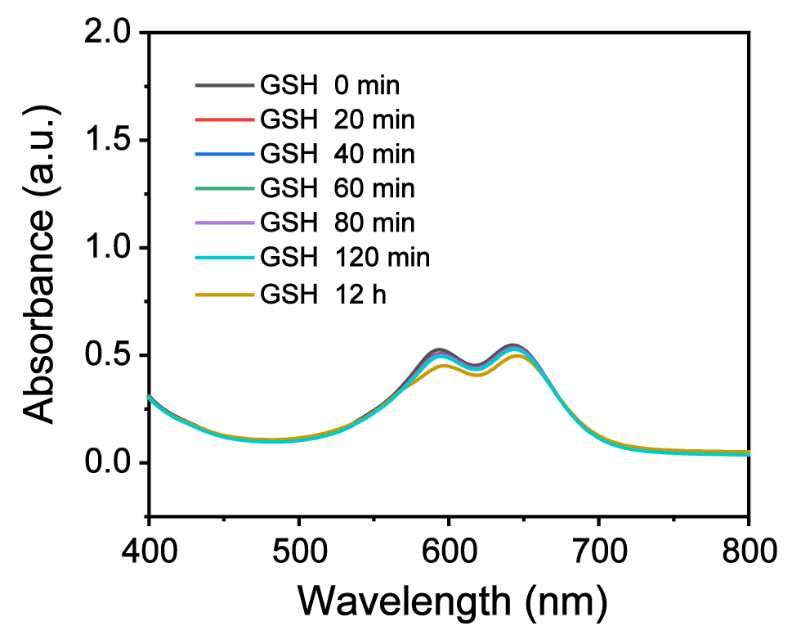
**

**Figure S22.** The absorption spectra of FeBPs with 5 mM glutathione (GSH) over various time periods.


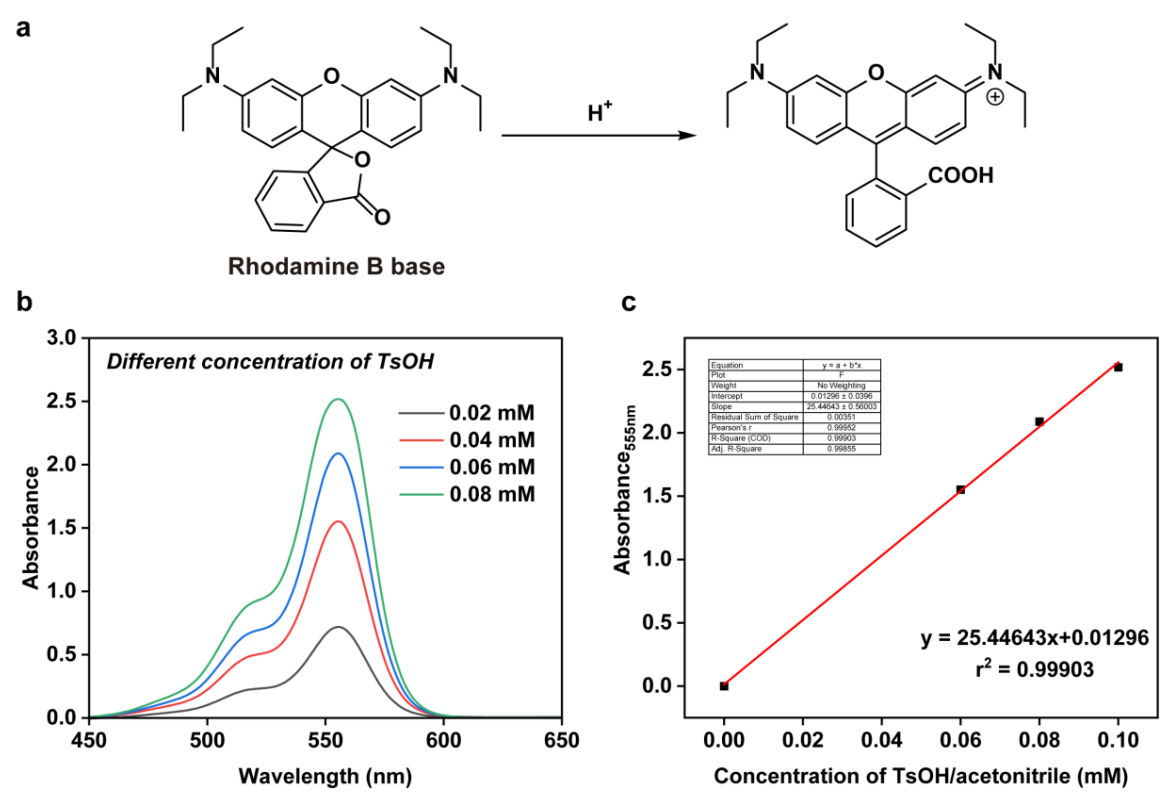


**Figure S23.** (a) Investigation of the protonation mechanism of Rhodamine B base acting as a H⁺ chromogenic indicator. (b) UV-Vis spectral analysis of varying concentrations of p-toluenesulfonic acid (TsOH), an organic acid, in an acetonitrile solution upon interaction with the Rhodamine B base. (c) Construction of a standard calibration curve depicting the relationship between absorbance values at 555 nm and H⁺ concentrations.


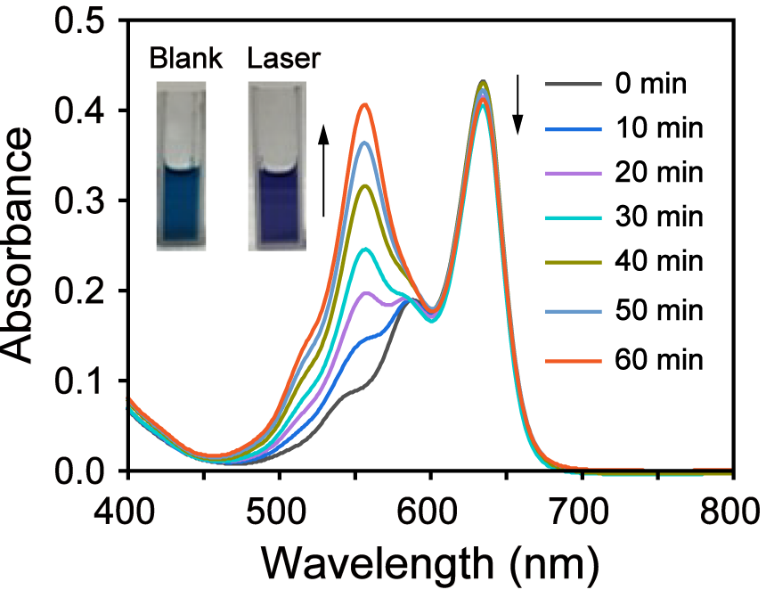


**Figure S24.** UV-vis absorption spectra of Rhodamine B base (50 μM) in CH_3_CN with PAGs-Bodipy (5 μM) under 630 nm light irradiation (0.33 W/cm^2^) at indicated time points. Insets: Photographs pre- and post-irradiation.


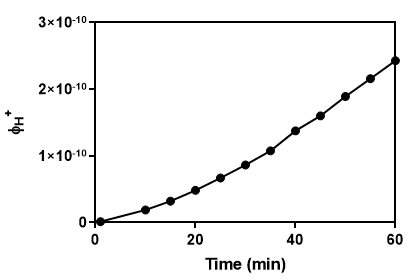


**Figure S25.** Time-dependent photoacid quantum yield.


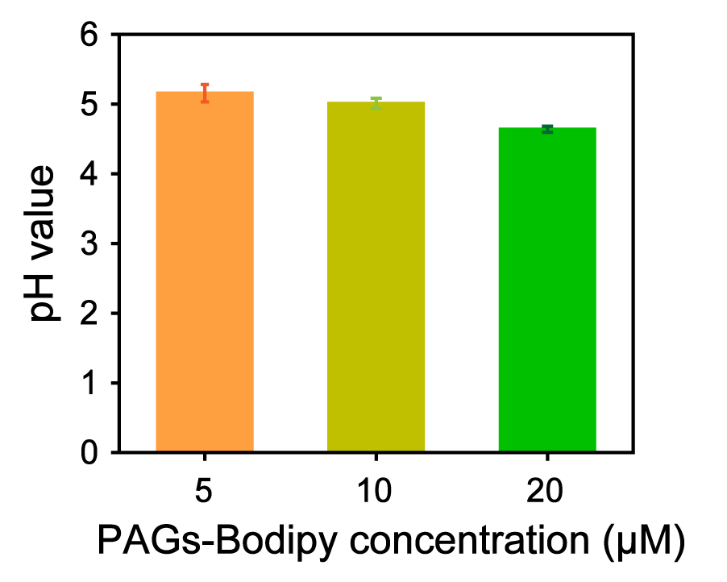


**Figure S26.** pH variation of PAGs-Bodipy solutions with different concentrations after 10 min of 630 nm irradiation.


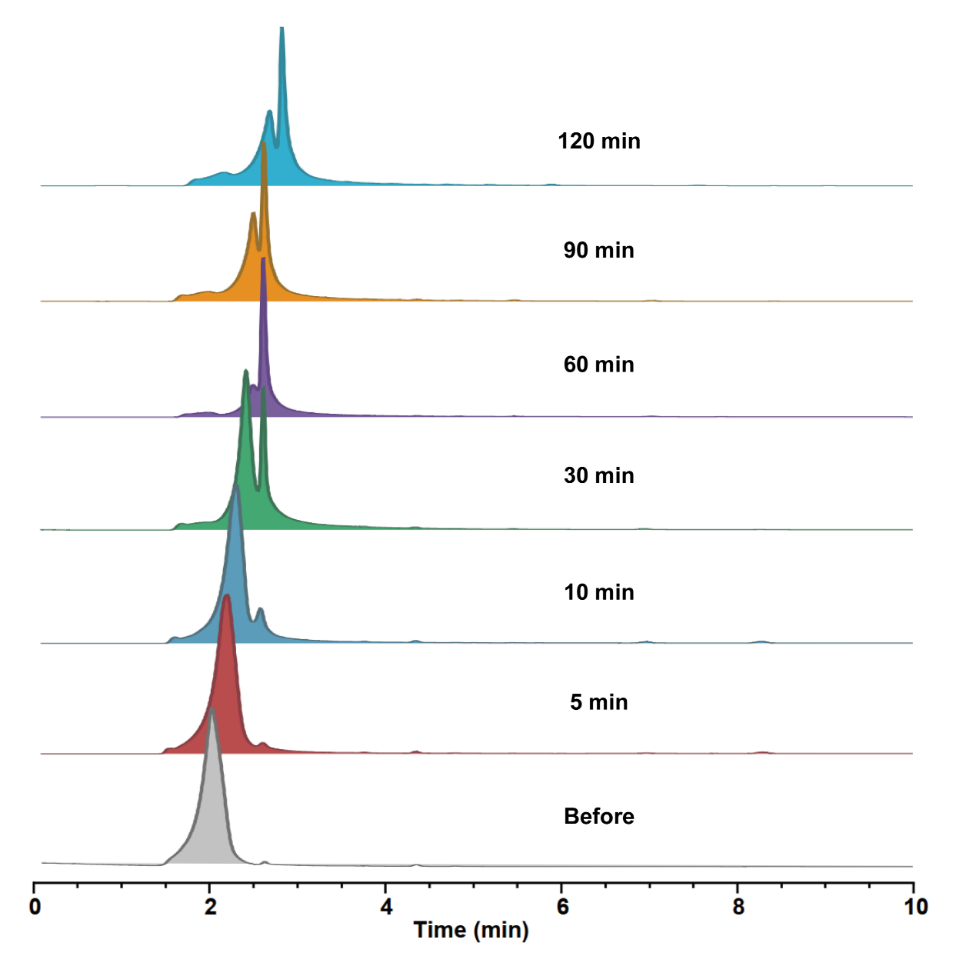


**Figure S27.** Photolysis of PAG-Bodipy (1 mg/mL, solution: CH_2_Cl_2_) before and after light irradiation (630 nm, 0.33 W/cm^2^) for different time intervals studied by HPLC (MeOH/H_2_O, 1:9, v/v).


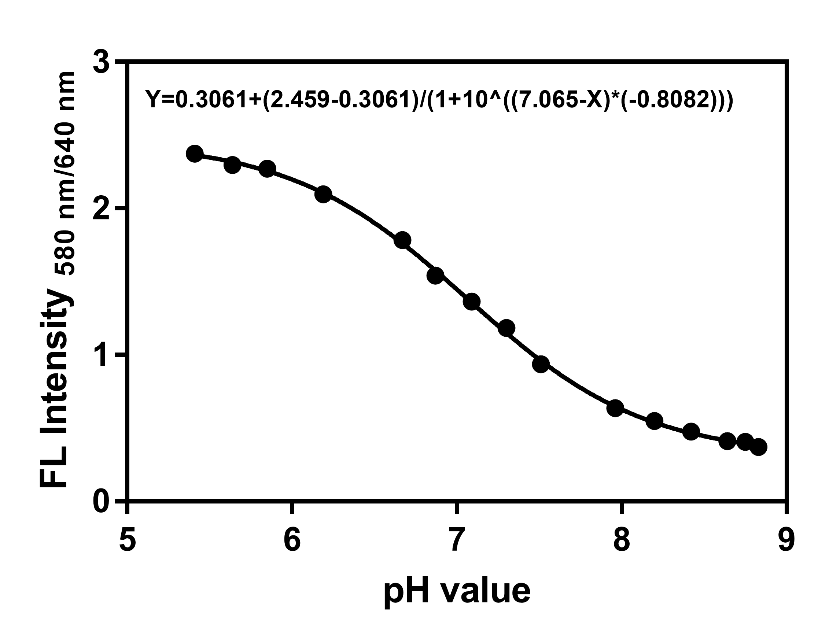


**Figure S28.** The fluorescent ratios of SNARF^TM^-1 buffer solutions at various pH levels measured at wavelengths of 580 nm and 640 nm, and the corresponding standard curves were plotted.

**Table S4.** EXAFS fitting parameters at the Fe *K*-edge for various samples. (*Ѕ*_0_^2^=0.77)

| Sample | Shell | CN^a^ | R(Å)^b^ | σ^2^(Å^2^)^c^ | ΔE_0_(eV)^d^ | K-range/Å^-1^ | R-range/Å | R factor |
| --- | --- | --- | --- | --- | --- | --- | --- | --- |
| Fe foil | Fe-Fe | 8* | 2.47±0.01 | 0.0051 | 6.6±0.9 | 3.0-14.2 | 1.0-3.0 | 0.0029 |
|  | Fe-Fe | 6* | 2.85±0.01 | 0.0066 |  |  |  |  |
| FeBPs following light irradiation | Fe-O | 3.0±0.2 | 2.10±0.01 | 0.0088 | 4.1±1.7 | 3.0-10.1 | 1.0-2.5 | 0.0155 |


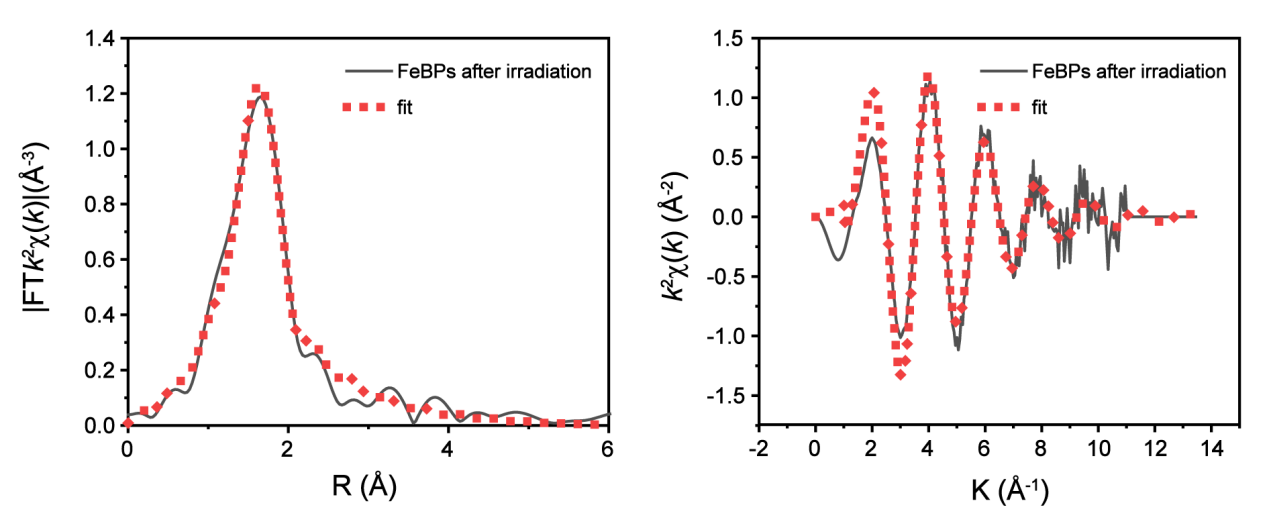


**Figure S29.** Nonlinear fitting of the EXAFS spectra for FeBPs after light exposure (R - space and k - space).


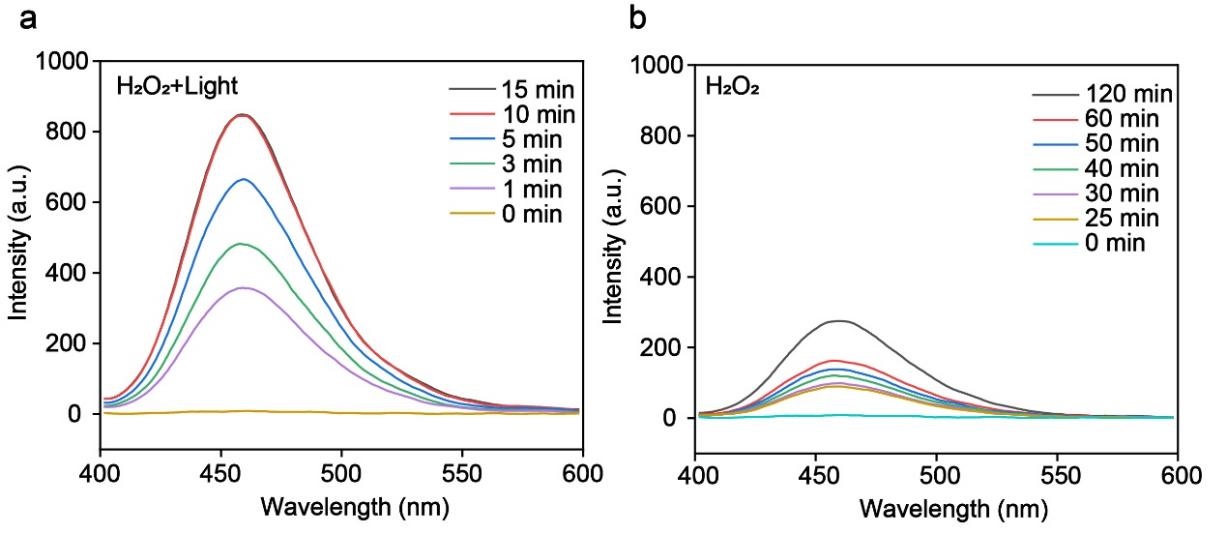


**Figure S30.** The fluorescent intensity of COU at various times when (a) FeBPs react with H_2_O_2_ under 630 nm light irradiation and (b) in the absence of light.


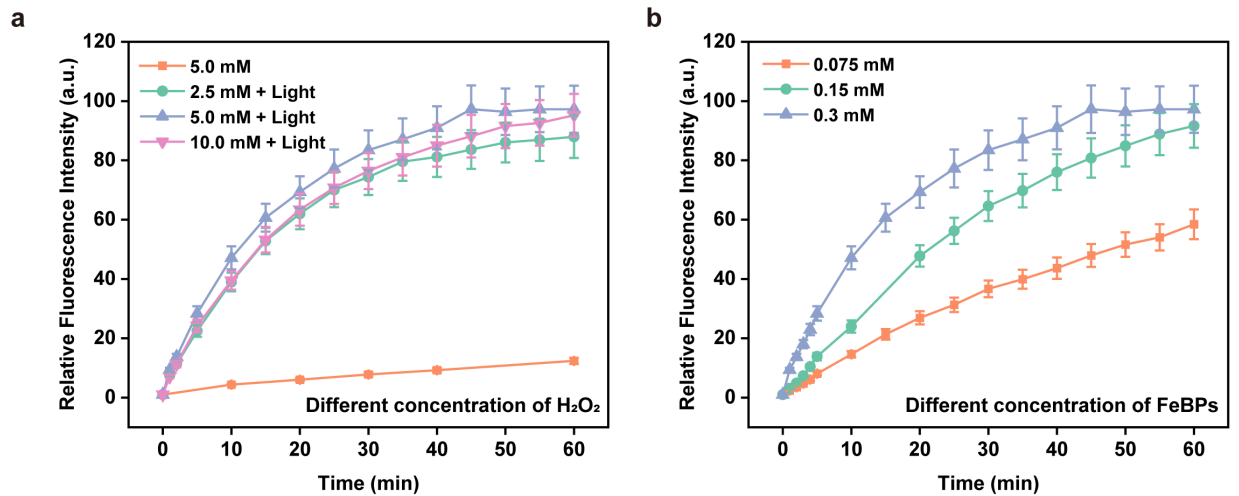


**Figure S31.** Fluorescence spectra of COU mixed with FeBPs and H_2_O_2_. (a) FeBPs (0.15 mM) reacted with different H_2_O_2_ concentrations (2.5, 5.0 and 10.0 mM) with or without light irradiation (630 nm, 0.33 W/cm^2^), and (b) different concentration of FeBPs (0.075 mM, 0.15 mM and 0.3 mM) reacted with same H_2_O_2_ concentrations (1.0 mM) with light irradiation (630 nm, 0.33 W/cm^2^).


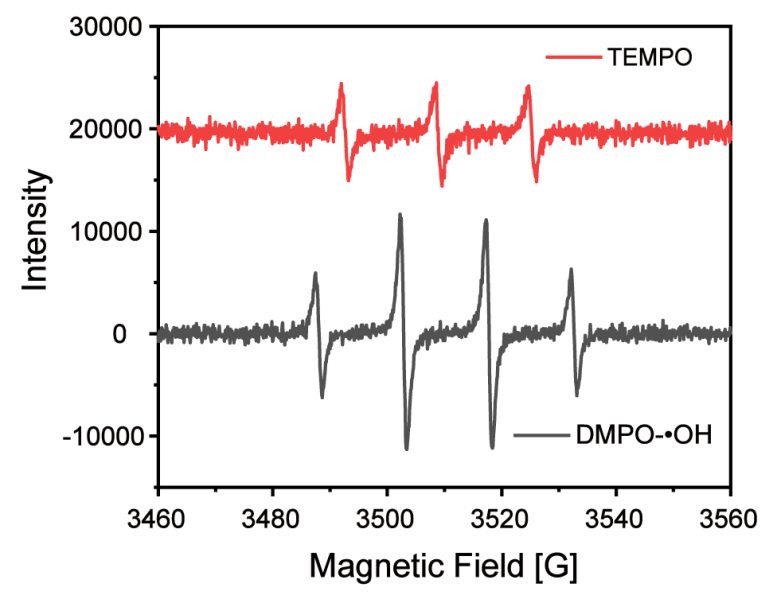


**Figure S32.** ESR spectra of the •OH probe (DMPO, 5,5-dimethyl-1-pyrroline N-oxide) and the ^1^O_2_ probe (TEMP, 2,2,6,6-tetramethylpiperidine) with same concentration of FeBPs under various treatments.


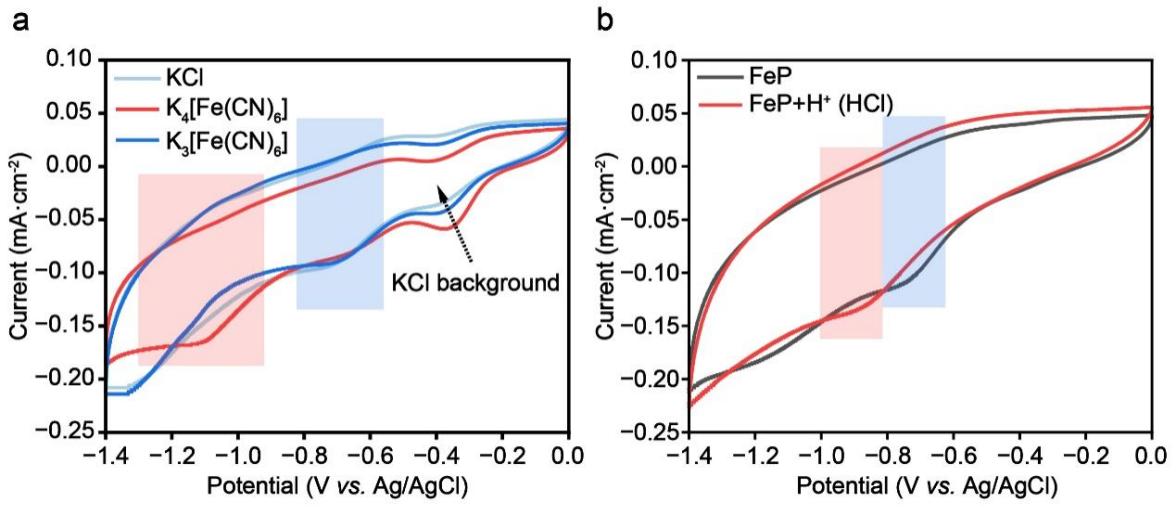


**Figure S33.** Cyclic voltammetry of (a) Fe^3+^ (K_3_[Fe(CN)_6_], 0.1 mM), Fe^2+^ (K_4_[Fe(CN)_6_], 0.1 mM) and KCl (1M), (b) FeP with and without H^+^ (C_H_^+^=0.01 mM).


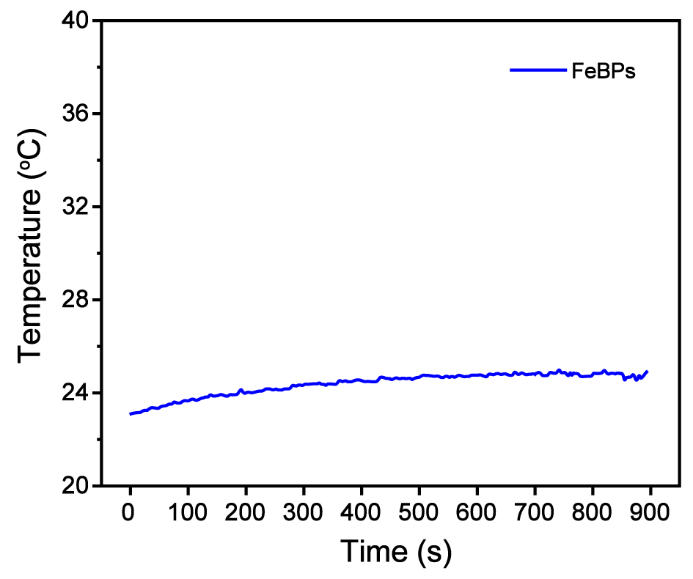


**Figure S34.** The heating curve of FeBPs irradiated with 630 nm light (0.33 W/cm^2^).


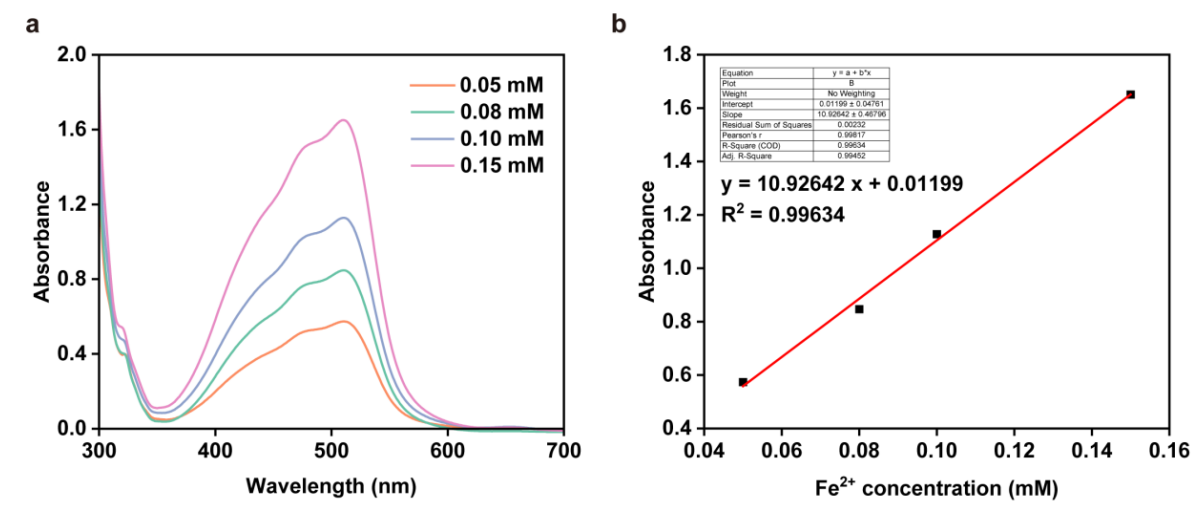


**Figure S35.** (a) UV-vis spectra of 1,10-phenanthroline in the presence of different concentration of Fe^2+^. (b) The corresponding standard curves with the absorption peak at 511 nm.


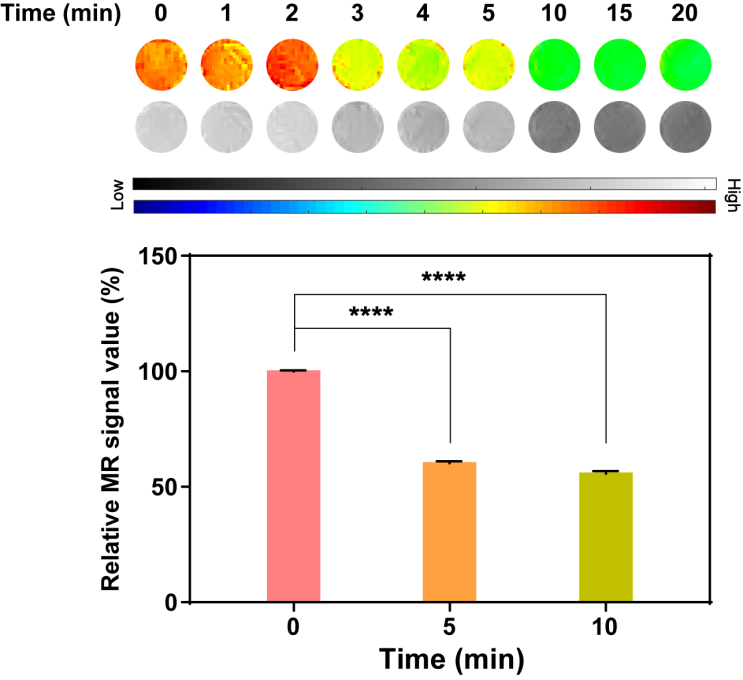


**Figure S36.** MR images of FeBPs with different 630 nm laser irradiation time (0.33 W/cm^2^) at 1.0 T.


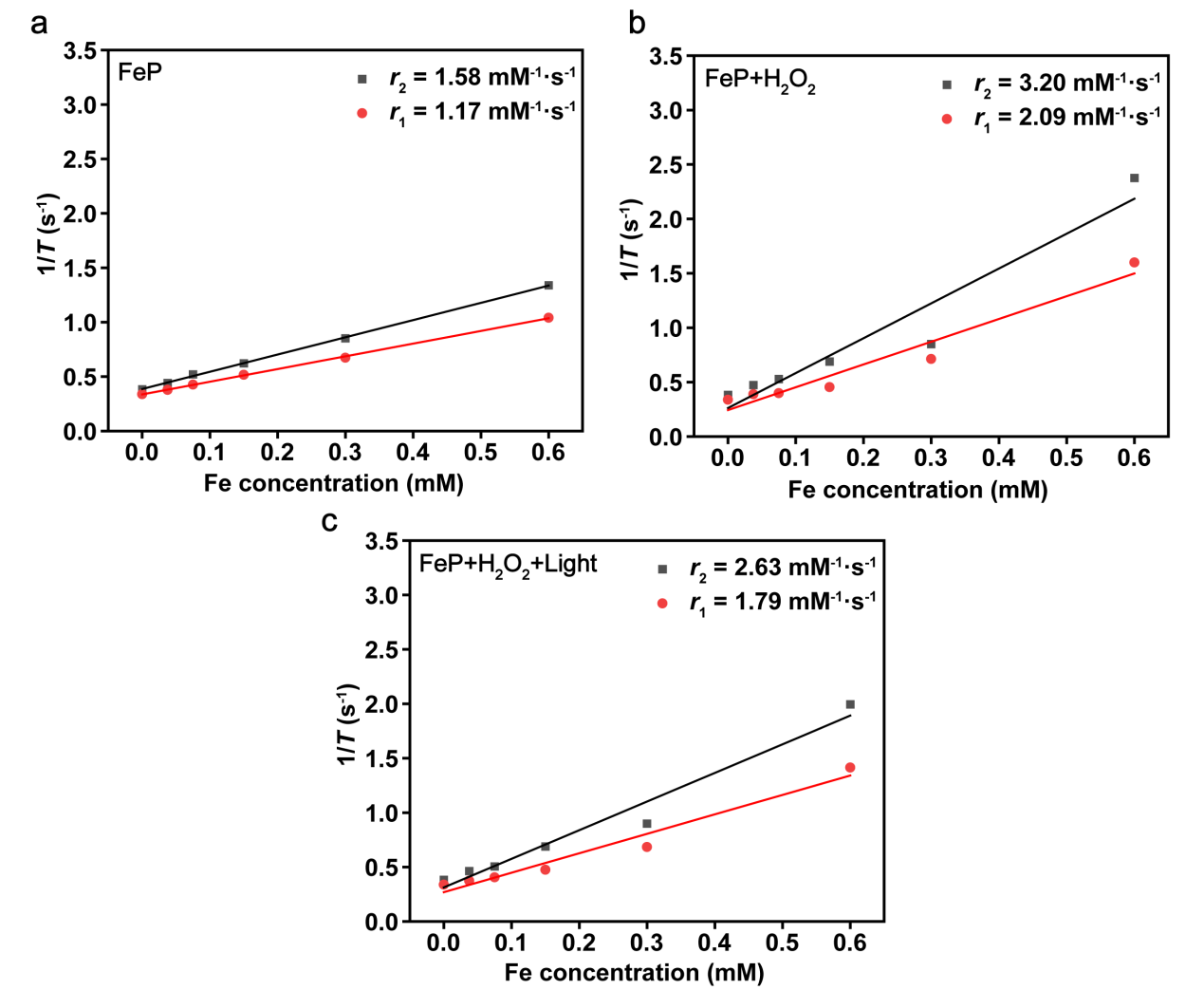


**Figure S37.** *r*_1_ relaxivity of FeP in different condition.


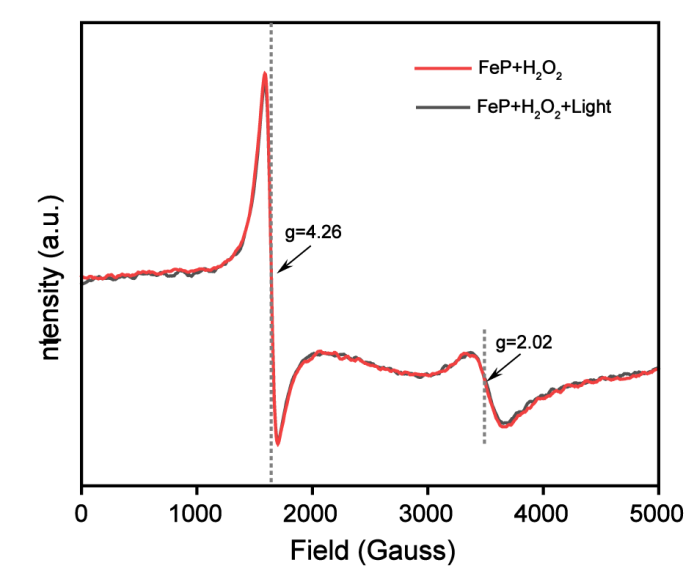


**Figure S38.** ESR spectra of FeP react with H_2_O_2_ in different conditions.


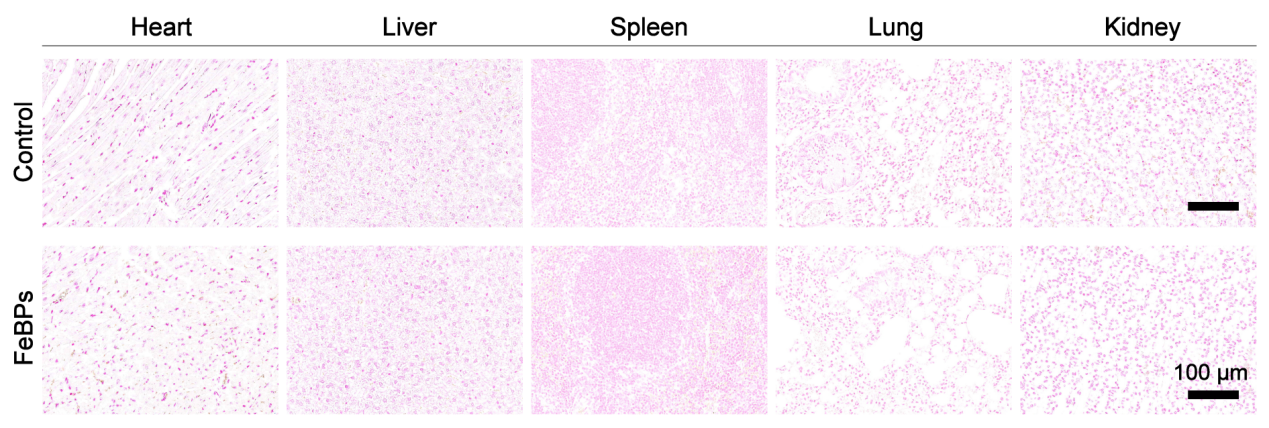


**Figure S39**. Prussian blue staining of main organs 24 hours after intravenously injection of FeBPs. The nucleus was stained with Hematoxylin. Scale bar: 100 μm.


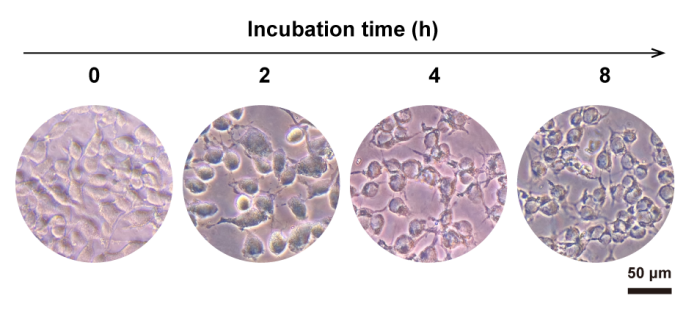


**Figure S40**. Prussian blue staining of B16-F10 cells incubated with FeBPs for different time.


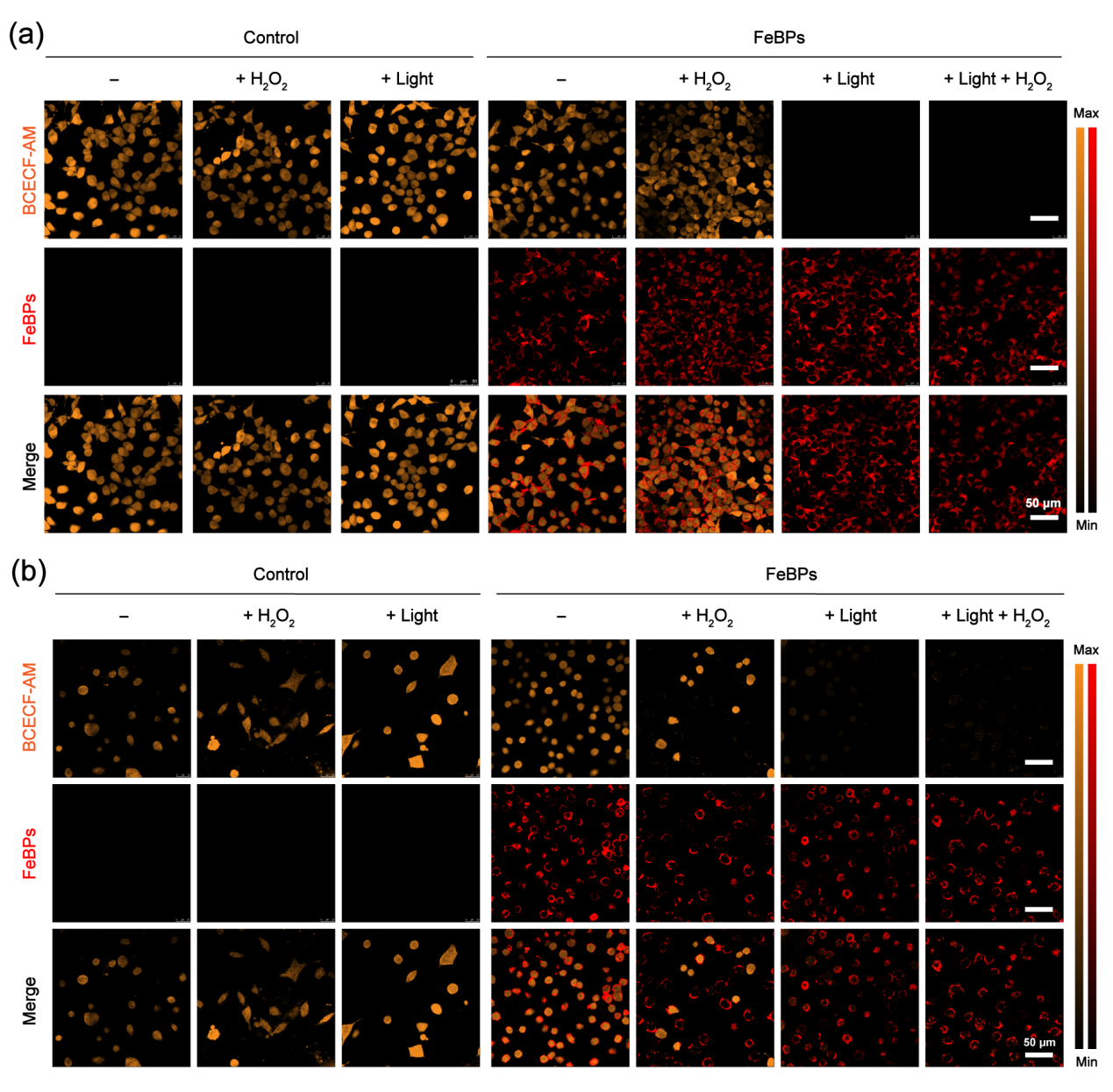


**Figure S41**. (a) B16-F10 cells and (b) A549 cells were treated with or without FeBPs under various conditions and stained with BCECF-AM to monitor intracellular acidification. BCECF-AM exhibits decreased fluorescence intensity with decreasing intracellular pH, allowing for the assessment of cytosolic acidification. Scale bar: 50 μm. λ_ex_/λ_em_ = 488/535 nm.


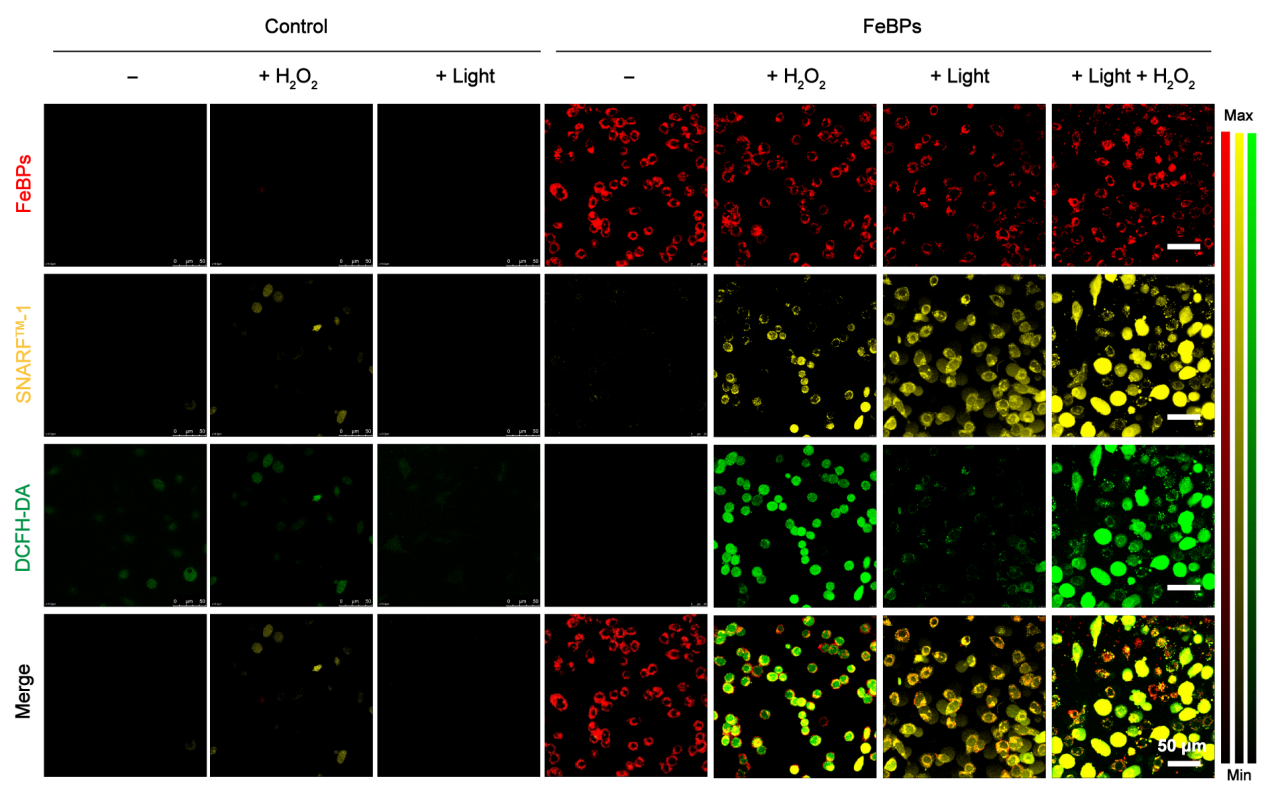


**Figure S42**. A549 cells were treated with or without FeBPs under various conditions and subsequently stained with SNARF^TM^-1 to monitor intracellular acidification and with DCFH-DA to oxidative stress. SNARF^TM^-1 exhibits increased fluorescence intensity with decreasing intracellular pH. DCFH-DA is a widely used fluorescent probe that detects intracellular reactive ROS by generating fluorescence upon oxidation to 2’,7’-dichlorofluorescein (DCF). The fluorescence intensity of DCF is proportional to the level of ROS in the cells. SNARF^TM^-1 (λ_ex_/λ_em_: 488 nm/580 nm), DCFH-DA (λ_ex_/λ_em_: 488/525 nm). Scale bar: 50 μm.


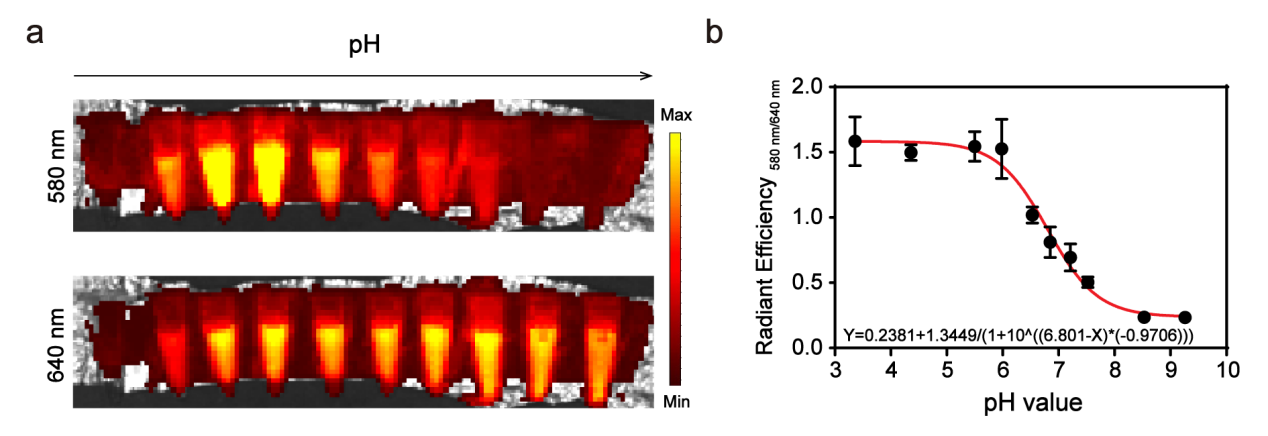


**Figure S43.** (a) Fluorescence images of various pH FeBPs and SNARF^TM^-1 probe mixtures at 580 nm and 640 nm wavelengths. (b) pH standard curve fitted based on dual-wavelength fluorescence emission efficiency.


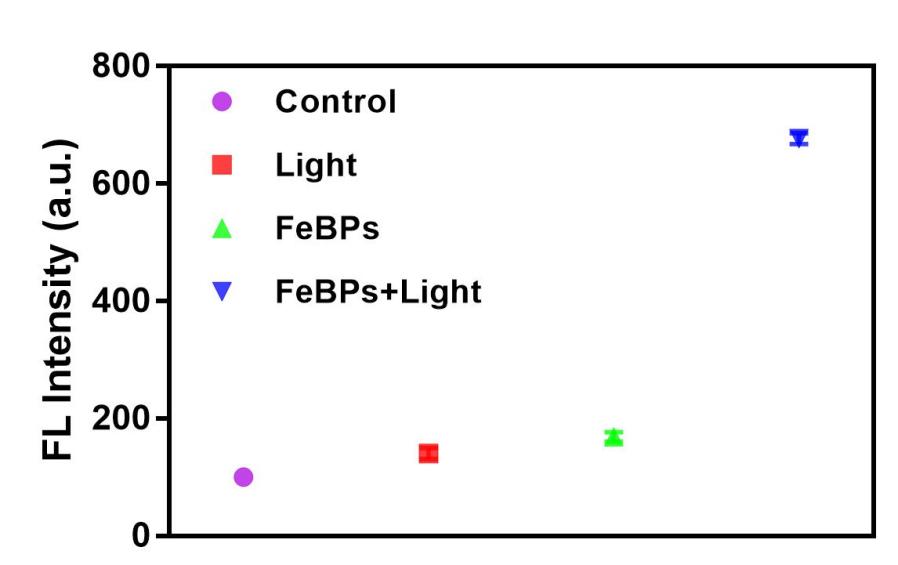


**Figure S44.** ROS levels analyzed by fluorescence intensity of tumor regions in **Figure 5f**.


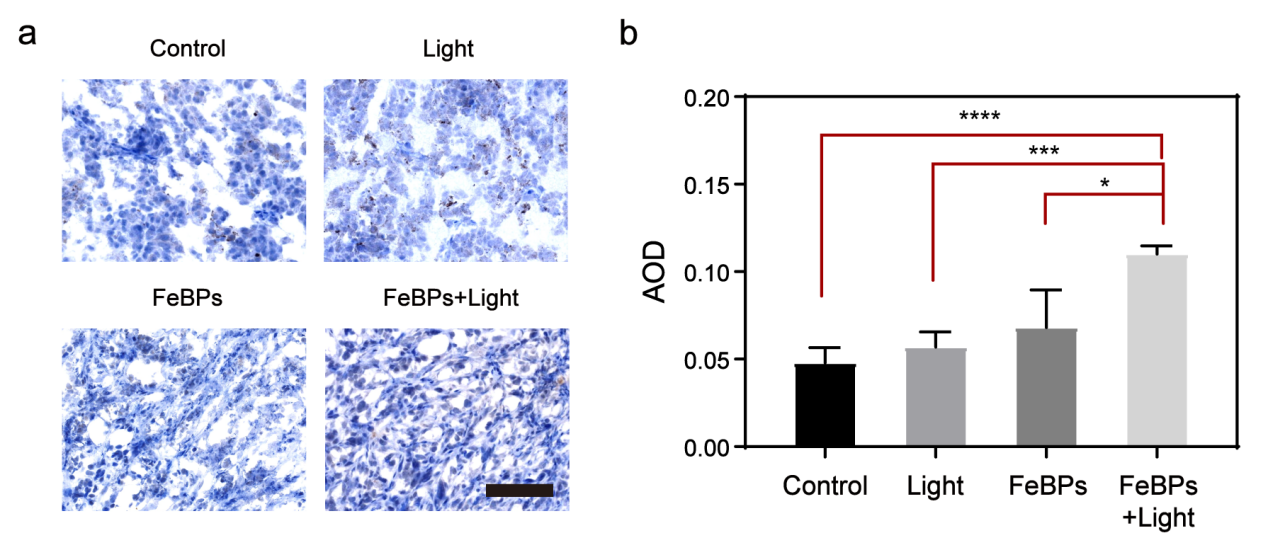


**Figure S45.** Immunohistochemical (IHC) staining of ex vivo tumor sections for 4-hydroxynonenal (4-HNE) with diaminobenzidine (DAB) staining. (a) Representative images of 4-HNE staining; brown signals indicate 4-HNE-positive areas (lipid peroxidation). Scale bar: 100 μm. (b) Quantitative analysis of 4-HNE levels across groups using Absorbance Optical Density (AOD, integrating positive area and intensity). Data are presented as mean ± SD (n = 3), with significant differences marked (*p < 0.05, ***p < 0.001, ****p < 0.0001).


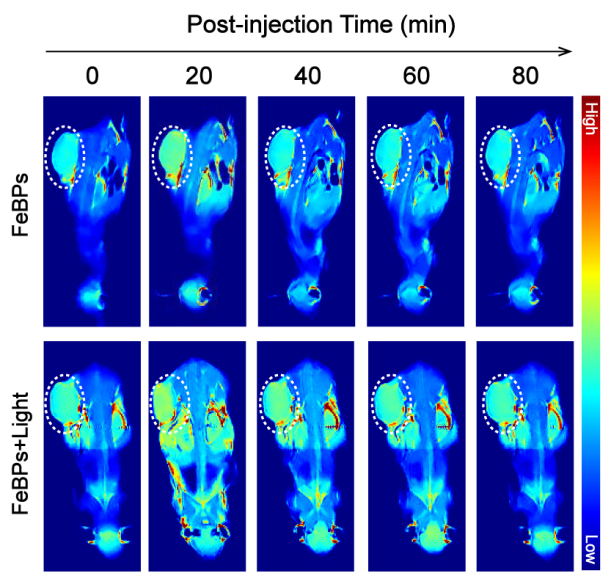


**Figure S46.** MRI pseudo-color images after intravenous injection of FeBPs with and without light irradiation. The white dashed box indicates the tumor area (n = 3).


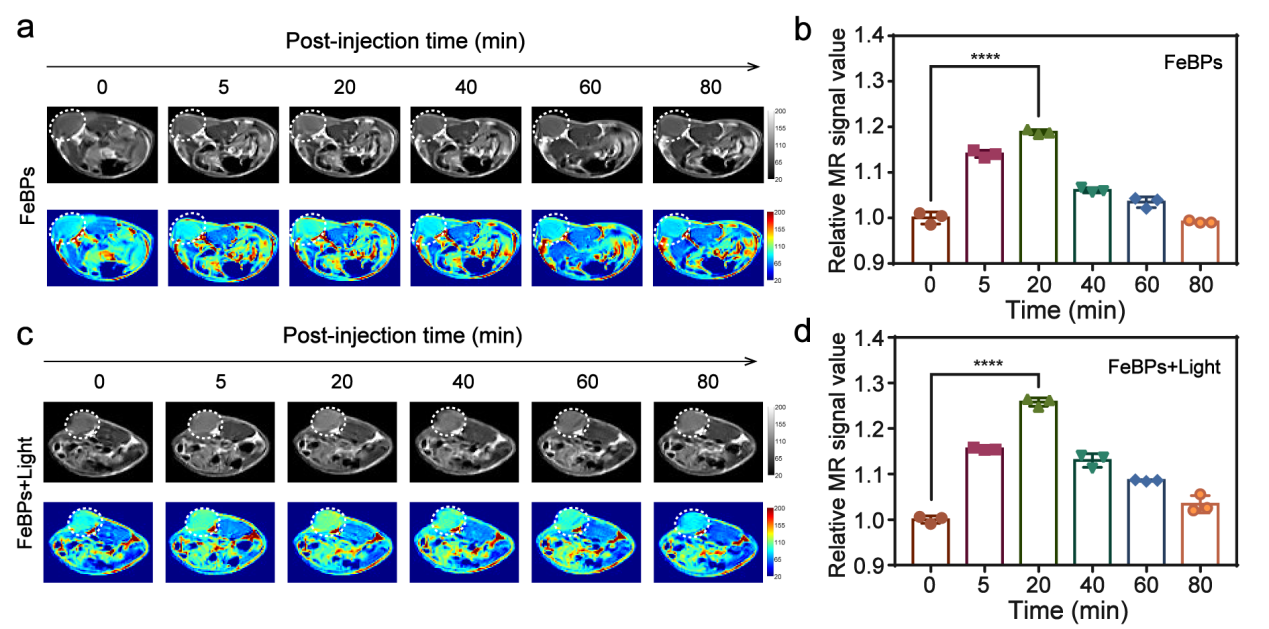


**Figure S47.** (a) Cross-sectional MRI images of the tumor after intravenous injection of FeBPs. (b) Corresponding signal intensity of (a). (c) Cross-sectional MRI imaging of the tumor after injection of FeBPs and irradiated with 630 nm light. (d) Corresponding signal intensity of (c). The white dashed box indicates the tumor area. Data were expressed as means ± SD (*n* = 3). Data were analyzed by *t*-test, ****p *<* 0.0001.


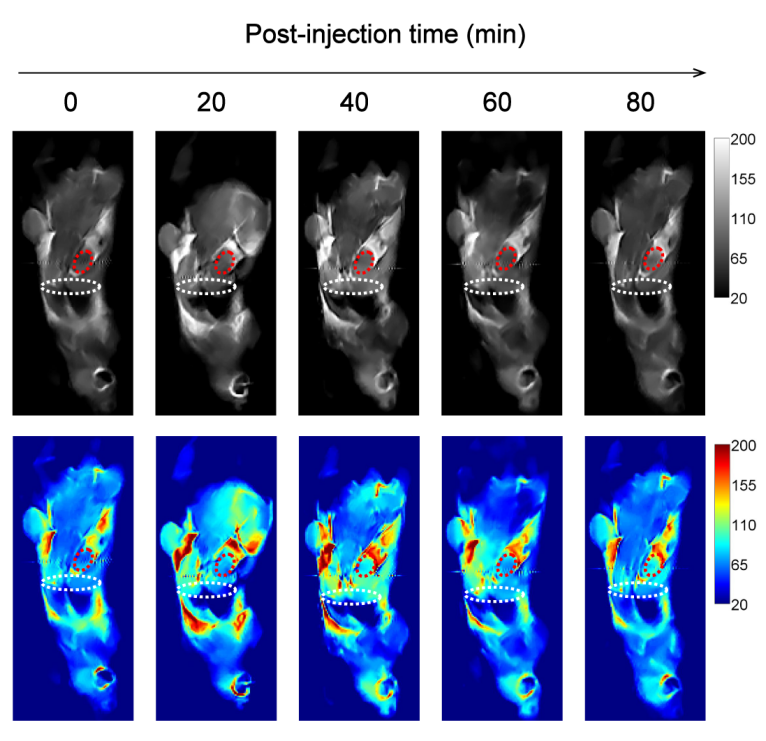


**Figure S48.** MRI images of the liver and kidney following the intravenous injection of FeBPs. The white dashed box indicates the liver of the mice, and the red dashed box indicates the kidney of the mice (n = 3).


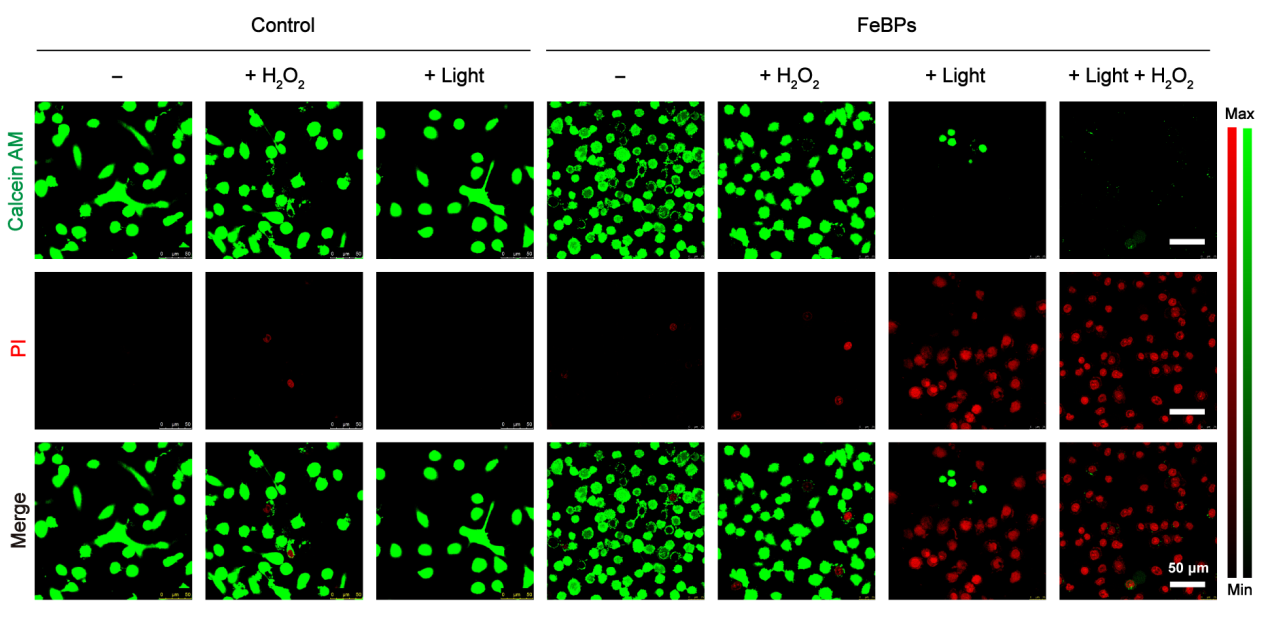


**Figure S49.** Confocal fluorescence images of A549 cells after different treatments and stained with live/dead cell staining kit. Calcein-AM (viable cells, green; λ_ex_/λ_em_ = 488/515 nm) and PI (dead cells, red; λ_ex_/λ_em_ = 535/617 nm). Scale bar: 50 μm.


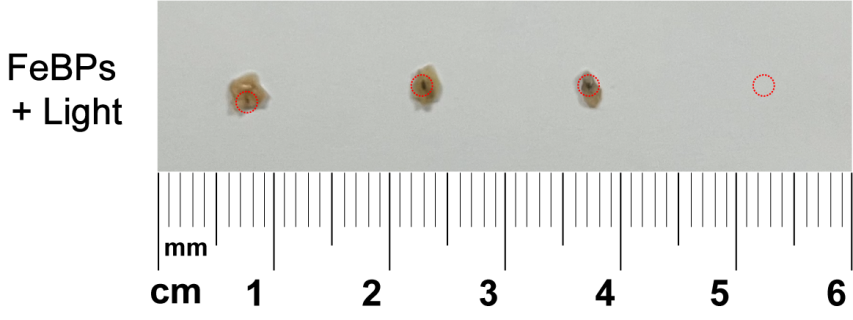


**Figure S50.** Magnified view of tumor tissue in the FeBPs + Light group of mice.


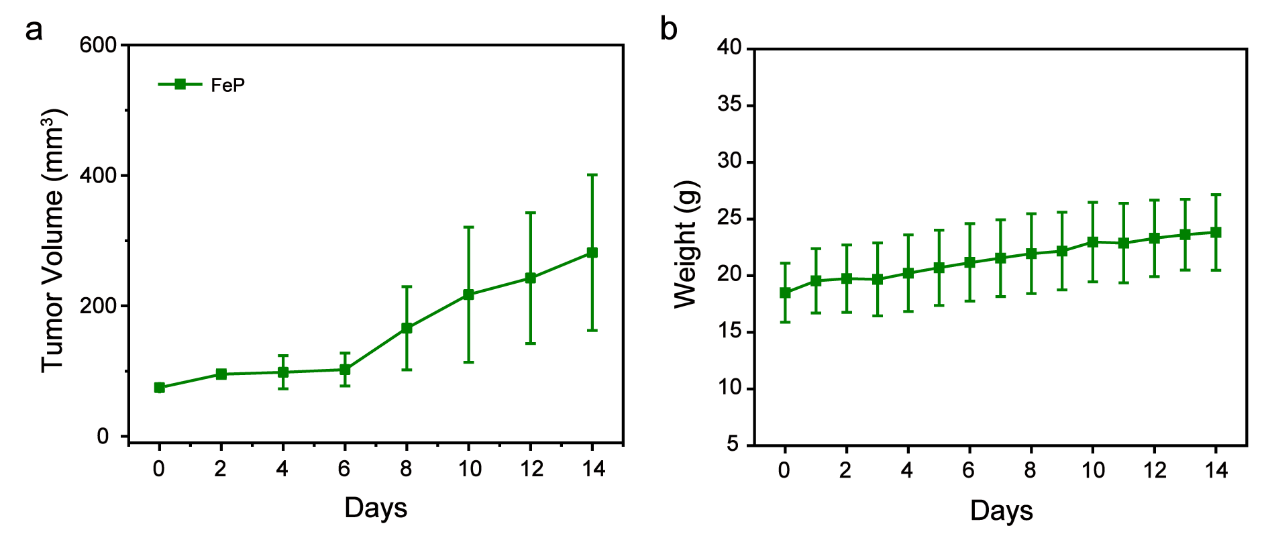


**Figure S51.** (a) Changes in tumor volume and (b) Variations in body weight of mice treated with FeP over a 14-day period.

**
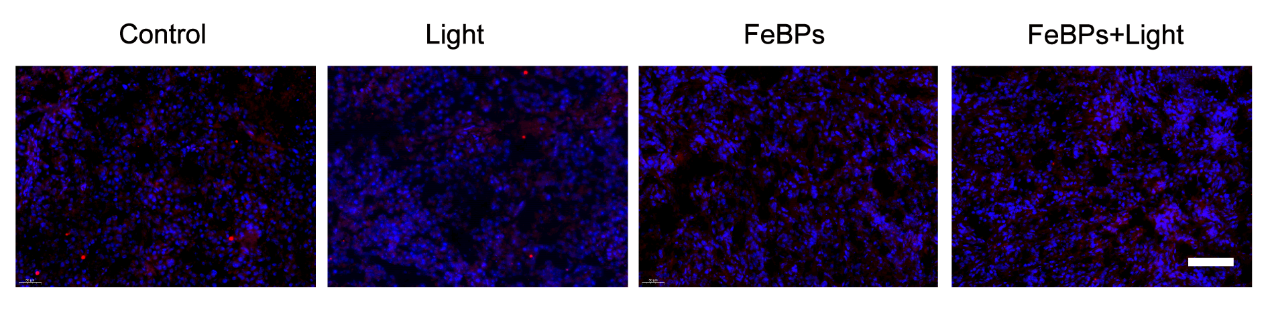
**

**Figure S52.** Typical staining of tumor sections for GPX4. Scale bar: 100 μm.

**25. References**

[1] K. Sambath, Z. X. Wan, Q. Wang, H. Chen, Y. W. Zhang, BODIPY-Based Photoacid Generators for Light-Induced Cationic Polymerization, *Org. Lett.* **2020,** *22*, 1208.

[2] L. He, M. F. Zhang, Z. Y. Pan, K. N. Wang, Z. J. Zhao, Y. Li, Z. W. Mao, A Mitochondria-Targeted Iridium(III)-Based Photoacid Generator Induces Dual-Mode Photodynamic Damage within Cancer Cells, *Chem. Commun.* **2019,** *55*, 10472.

[3] F. P. V. Paoloni, S. Kelling, J. Huang, S. R. Elliott, Sensor Array Composed of “Clicked” Individual Microcantilever Chips, *Adv. Funct. Mater.* **2011,** *21*, 372.

[4] Y. Wan, L. An, Z. X. Zhu, Q. W. Tian, J. M. Lin, S. P. Yang, Iron-Polyphenol Dendritic Complexes for Regulating Amplification of Phenolic Hydroxyl Groups to Improve Magnetic Resonance Imaging, *Chem. Eng. J.* **2023,** *458*, 141322.

[5] L. Q. Xu, F. Yao, G. D. Fu, E. T. Kang, Interpenetrating Network Hydrogels via Simultaneous “Click Chemistry” and Atom Transfer Radical Polymerization, *Biomacromolecules* **2010,** *11*, 1810.

[6] C. J. Yang, J. P. Flynn, J. Niu, Facile Synthesis of Sequence-Regulated Synthetic Polymers Using Orthogonal SuFEx and CuAAC Click Reactions, *Angew. Chem. Int. Ed.* **2018,** *57*, 16194.

[7] B. Ravel, M. Newville, ATHENA, ARTEMIS, HEPHAESTUS: Data Analysis for X-ray Absorption Spectroscopy using IFEFFIT, *J. Synchrotron Radiat.* **2005,** *12*, 537.

[8] S. I. Zabinsky, J. J. Rehr, A. Ankudinov, R. C. Albers, M. J. Eller, Multiple-Scattering Calculations of x-ray-absorption Spectra, *Phys. Rev. B* **1995,** *52*, 2995.

[9] J. P. Perdew, J. A. Chevary, S. H. Vosko, K. A. Jackson, M. R. Pederson, D. J. Singh, C. Fiolhais, Atoms, Molecules, Solids, and Surfaces: Applications of the Generalized Gradient Approximation for Exchange and Correlation, *Phys. Rev. B* **1992,** *46*, 6671.

[10] P. E. Blöchl, Projector Augmented-Wave Method, *Phys. Rev. B* **1994,** *50*, 17953.

[11] J. P. Perdew, Y. Wang, Accurate and Simple Analytic Representation of the Electron-Gas Correlation Energy, *Phys. Rev. B* **2018,** *98*, 079904.

[12] S. Grimme, Semiempirical GGA-Type Density Functional Constructed with a Long-Range Dispersion Correction, *J. Comput. Chem.* **2006,** *27*, 1787.
